# Chiral Polythiophenes: Part II: Recent Advances in Synthesis, Structural Determinations and Applications

**DOI:** 10.3390/ijms27219706

**Published:** 2026-10-29

**Authors:** Dorota Krasowska, Rafał Karpowicz, Józef Drabowicz

**Affiliations:** 1 Division of Organic Chemistry, Centre of Molecular and Macromolecular Studies, Polish Academy of Sciences, Sienkiewicza 112, 90-363 Lodz, Poland; jozef.drabowicz@cbmm.lodz.pl; 2 Division of Structural Chemistry, Centre of Molecular and Macromolecular Studies, Polish Academy of Sciences, Sienkiewicza 112, 90-363 Lodz, Poland; 3 Department of Organic Chemistry, Faculty of Chemistry, University of Łódź, Tamka 12, 91-403 Lodz, Poland; rafal.karpowicz@chemia.uni.lodz.pl; 4 Department of Chemistry, Jan Dlugosz University, Armii Krajowej 13/15, 42-200 Czestochowa, Poland

**Keywords:** π-conjugated polymers, chiral polythiophenes, stereogenicity, supramolecular helical chirality, chiroptical methods, electropolymerization, chemical polymerization, electrochromism, redox switching, chiral sensing, enantiodifferentiation

## Abstract

This review aims to: (a) highlight key strategies for preparing non-racemic polythiophenes as single chemical entities or supramolecular thin-film architectures, (b) discuss their essential structural features and optical stability, and (c) overview their applications as chiral sensors, chiral catalysts, and optical materials. Special emphasis is placed on recent advances in the synthesis of chiral polythiophene, higher-order molecular organization driven by point, through-space, planar or axial stereogenicity, structure–property relationships, and the intended applications of these materials.

## 1. Introduction

Polythiophenes (PThs) represent versatile classes of π-conjugated polymers that have been widely investigated because they combine the electronic and optical properties of conjugated backbones with a well-defined structural architecture and/or chiral supramolecular assemblies [1,2,3]. The structural versatility of the thiophene ring enables precise molecular tailoring, giving rise to various subfamilies. In terms of structural rigidity, polythiophenes can be divided into unsubstituted polythiophenes and those substituted with side chains; the latter offer better solubility and processability. Based on regiochemistry, 3-substituted PThs can be split into regioregular polymers which have head-to-tail (HT) coupling at the α,α′-positions of the adjacent thiophene rings, and regiorandom polymers in which the substitution pattern alternates irregularly between the 3 and 4 positions along the polymer backbone. This report also covers donor–acceptor (D–A) thiophene-based copolymers.

Combining electron delocalization with molecular and supramolecular chirality yields advanced chiroptical phenomena, including intense circular dichroism (CD), circularly polarized luminescence (CPL), and chirality-induced spin selectivity (CISS) [4]. These properties make chiral polythiophenes promising for organic spintronics, molecular electronics, smart displays [5,6] enantioselective biosensors [7], asymmetric catalysis [8], and bioactive materials [9].

Molecular chirality originates from the presence of stereogenic atoms, chiral axes, or chiral planes (elements of stereogenity) within a single molecule. Molecular chirality, introduced via stereogenic elements or external chiral environments (chiral solvents, liquid crystals, chiral nanostructures, metal–organic frameworks (MOFs), and circularly polarized light (CPL) [10], transfers to the polymer backbone to form higher-order helical assemblies. This supramolecular chirality is governed by π → π* interactions, solvent-induced aggregation (generating bisignate Cotton effects), side-chain architecture, and thermal/processing kinetics. The reviewed studies highlight that optical activity in π-conjugated systems is governed more by supramolecular organization rather than molecular chirality alone. 

Over the past decades, numerous synthetic strategies have been developed to prepare chiral polythiophenes and related copolymers [11,12]. Methods such as Kumada catalyst-transfer polycondensation (KCTP), Suzuki coupling, Stille polycondensation, and electrochemical polymerization have enabled the synthesis of π-conjugated systems with controlled regioregularity, molecular weight, and end-group functionality. 

In recent years, several comprehensive or update reviews on chiral polythiophenes have been published. Ozturk et al. reviewed developments in the preparation and properties of regioregular thiophene-based π-conjugated polymers from 2012 to 2019 [13]. In 2021, Yin Xiao et al. published a review in Chirality [1] summarizing structural studies and applications over the preceding decade. M’hamed Chahma reviewed the applications of doped polythiophenes as biosensors in a 2021 report [7]. A chapter entitled “Chiral Conductive Polymers” was published in 2022 in a book series edited by Amiri and Hussain, and covers key aspects and potential applications of conductive polymers [14]. Similarly, a chapter titled “Application of Conductive Polymers in Electrochemistry” was published in the same series [9]. This review summarizes recent progress (2012–2025) in the synthesis, self-assembly, chiroptical behavior, and applications of chiral polythiophenes and related conjugated copolymers. It emphasizes advances in synthetic strategies, structural insights, and key phenomena such as supramolecular helicity, exciton coupling, and molecular recognition.

## 2. Phenomenon of Chirality of Polythiophenes

### 2.1. Origin and Generation of Chirality

Molecular chirality arises from covalent asymmetry within a single molecule and yields enantiomers that often have distinct physicochemical and biological properties. In polythiophenes, chirality transfer occurs when a stereogenic element (an asymmetric center, axis, or plane) in the side chains guides the polymer backbone to twist and pack into preferred left-handed (M) or right-handed (P) helices, as seen in helicenes and DNA. Therefore, chirality extends to macromolecular and supramolecular levels, producing helical structures that arise from non-covalent interchain assemblies. Beyond side-chain-directed transfer, optical activity can also be imparted externally through induced chirality driven by external environments. In this way, fully achiral polymers can still exhibit induced supramolecular chirality organized by a chiral template, driven by chiral solvents, liquid-crystalline media, metal-organic frameworks (MOFs), or circularly polarized light (CPL). In summary, strategies for generating chirality include direct covalent mechanisms that depend on the presence of a stereogenic element within structures and non-covalent routes that induce chirality through external environmental factors. 

### 2.2. Chiroptical Methods 

Optical activity is a physical property of a compound that rotates the plane of polarization of linearly polarized light. Optically active structures exist as two mirror images (enantiomers) that differ in the sign of their optical rotation. Optical activity was historically measured by optical rotatory dispersion (ORD) but is now primarily probed using circular dichroism (CD). Circular dichroism (CD) measures the difference in absorbance (Δε) between left- and right-handed circularly polarized light in the UV-VIS region [15]. CD spectroscopy provides deeper insight into chromophore organization and exciton coupling within aggregated structures, as well as monitoring conformational changes within the polythiophene backbone.

A monosignate Cotton effect occurs when an electronic transition in a chromophore generates a single band (peak or valley) in the CD spectrum. The bisignate Cotton effect is a specific CD outcome that occurs only when interacting chromophores aggregate in a chiral orientation and both split transitions remain optically active. This mechanism is called a Davydov splitting. In other words, the transition dipole moments of the adjacent chromophores undergo exciton coupling. These electrostatic dipole–dipole interactions split into two distinct collective exciton states (E_+_ and E_−_). In a chiral (helical or twisted) arrangement, Davydov splitting causes the two split states to absorb left- and right-circularly polarized light, producing two adjacent bands with opposite signs of the bisignate Cotton effect in the CD spectrum. The sign sequence of these split bands (positive-then-negative vs. negative-then-positive as wavelength decreases) directly indicates whether the supramolecular twist is right-handed (P-helix) or left-handed (M-helix). Davydov splitting is the spectroscopic footprint of supramolecular organization in the field of chiroptical profile of chiral self-assembly in π-conjugated materials, directly reflecting aggregate packing in the CD spectrum. A parameter used in chiroptics to quantify the ability of a chiral molecule to selectively absorb circularly polarized light is the absorption dissymmetry factor *g*_abs_. Its magnitude quantifies the difference between the molar absorption coefficients for left- and right-circularly polarized light divided by their average or sum (Equation (1)) to yield a value between −2 and +2, whose sign informs about the absolute configuration or handedness (P/M or d/L) of the chiral system.

(1) $$g_{abs}=\frac{\varepsilon_{L}-\varepsilon_{R}}{\frac{1}{2}(\varepsilon_{L}+\varepsilon_{R})}$$

Furthermore, many conjugated polymers exhibit circularly polarized luminescence (CPL). CPL spectroscopy measures the differential emission of left- and right-circularly polarized light (ΔI = I_L_ − I_R_). A positive value (+) indicates a predominance of left-circularly polarized light emission (L-CPL), while a negative value (−) indicates a predominance of right-circularly polarized light emission (R-CPL), which helps determine the stereochemistry and conformation of chiral molecules in the excited state (CPL) (or in the ground state if Δε from CD spectrum). However, the CD and CPL pathways differ in a few ways. CPL emission behaves differently due to Kasha’s rule, i.e., expressing distinct information in a non-radiative relaxation, exciton based on the absorption of a photon and transition to a higher energy level, monosignate dominance in CPL, which is due to emission originating predominantly from E_−_, producing a monosignate CPL spectrum rather than a bisignate one. The normalized parameter calculated from excited-state emission to quantify chiroptical strength in CPL spectroscopy is the luminescence dissymmetry factor, glum (Equation (2)).

(2) $$g_{lum}=\frac{I_{L}-I_{R}}{\frac{1}{2}(I_{L}+I_{R})}$$

CD and CPL are essential for tracking from the molecular scale to large supramolecular aggregates, with responses highly sensitive to molecular packing, aggregate morphology, film organization, and external factors such as redox switching or thermal annealing. 

#### 2.2.1. CD Spectroscopy

The self-assembly organization of poly{3,4-bis[(*S*)-2-methylbutoxy]thiophene} (PDMBT) was investigated as early as 1996 Langeveld-Voss et al. [16] using CD and CPL. Cooling a solution of PDMBT in dichloromethane to −30 °C induced ordered aggregation, as evidenced by a significant red-shift in the onset of absorption in the CD spectrum and emission in the CPL spectrum. 

**Figure 1:**
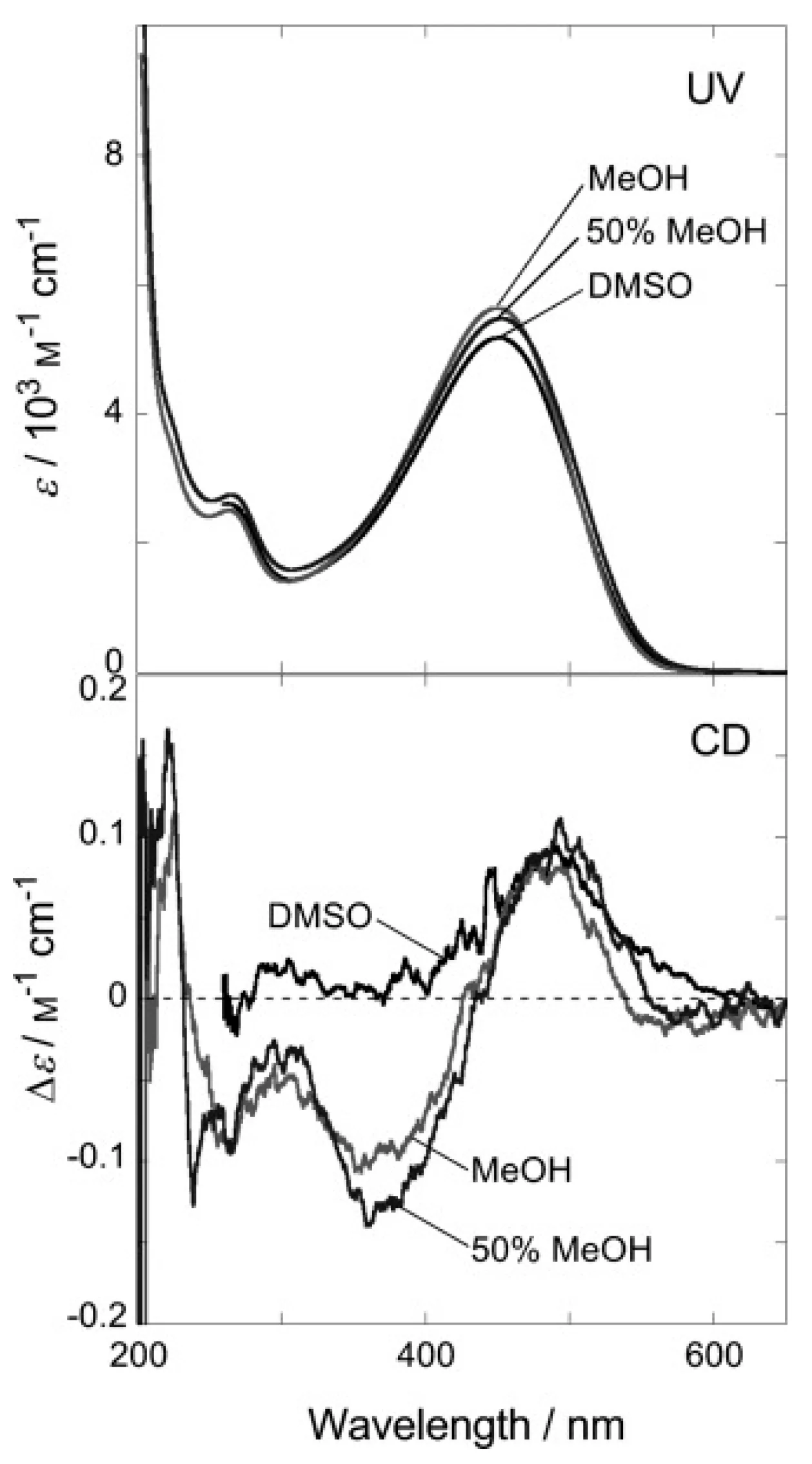
UV/Vis and CD spectra in DMSO (0.406 mM), methanol (0.138 mM), and 50% aqueous methanol (0.129 mM) (all concentrations in the monomer unit). Reprinted with permission from Fukuhara, G. and Inoue, Y. (2012), Peptide Chirality Sensing by a Cyclodextrin–Polythiophene Conjugate. Chem. Eur. J.; 18: 11459–11464. https://doi.org/10.1002/chem.201201294 [17]. Published by John Wiley and Sons. Copyright © 2012 WILEY-VCH Verlag GmbH & Co. KGaA, Weinheim.

Another example involves a cyclodextrin-functionalized polythiophene whose chiroptical properties were investigated in dimethyl sulfoxide (DMSO), methanol, and 50% aqueous methanol using UV-Vis and circular dichroism (CD) spectroscopies [17] (Figure 1). The UV-Vis absorption spectra in all solvent systems exhibited a characteristic π → π* electronic transition centered at 450 nm. In the CD spectra, weak signals arising from induced chirality were observed within this π → π* transition region. Pure or aqueous methanol promotes a bisignate CD effect featuring a positive couplet, indicative of a right-handed helix, whereas DMSO yields a positive monosignate signal. These distinct chiroptical profiles demonstrate that the induced chiral conformation of the polymer backbone is heavily dependent on the solvation environment.

**Figure 2:**
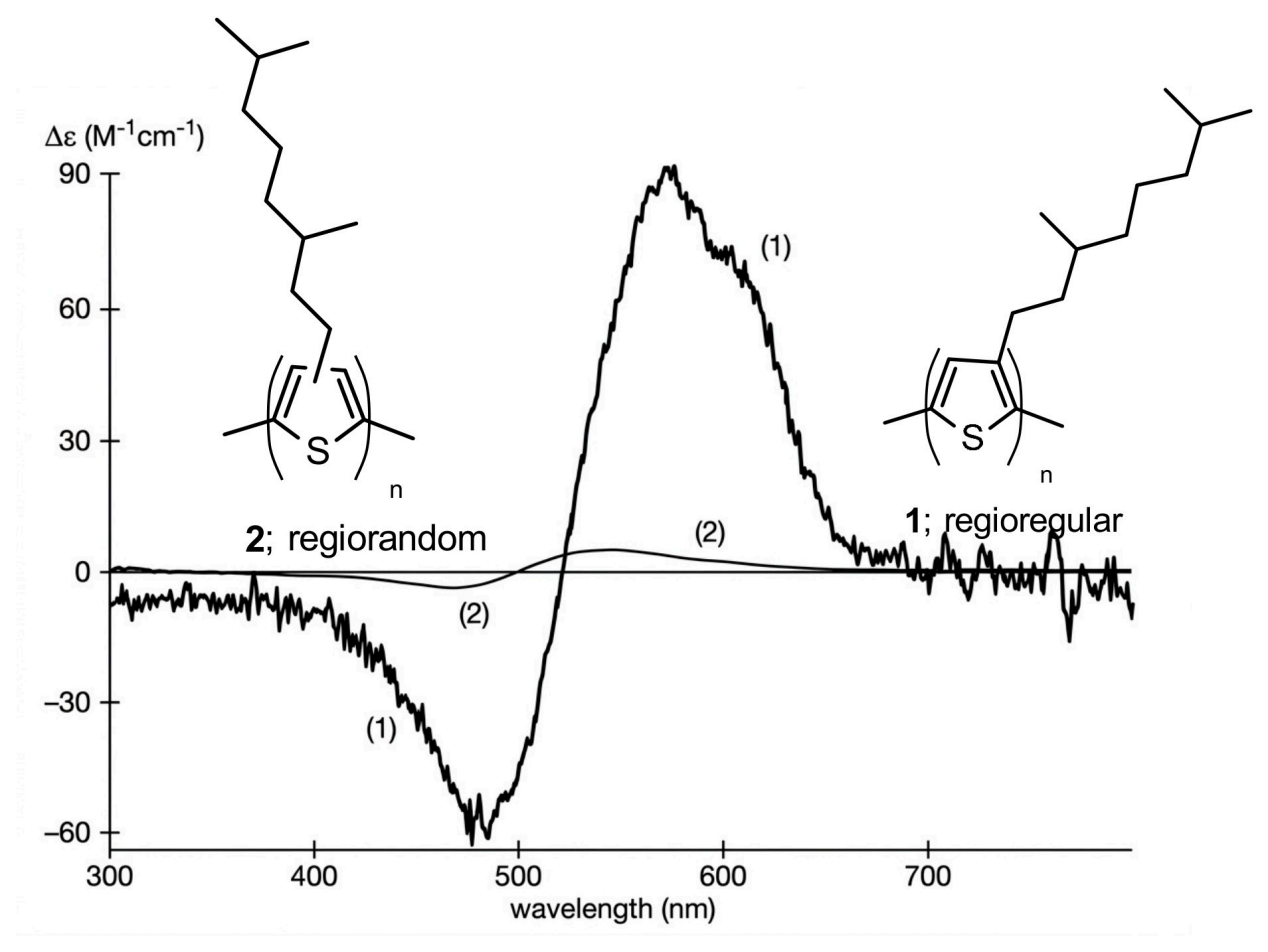
CD spectra of **1** and **2** differing in regioregularity in the solid state. Reprinted with permission from Bidan, G.; Guillerez, S. and Sorokin, V. (1996), Chirality in regioregular and soluble polythiophene: An internal probe of conformational changes induced by minute solvation variation ^†^. Adv. Mater., 8: 157–160. https://doi.org/10.1002/adma.19960080213 [18]. Published by John Wiley and Sons. Copyright © 1996 Verlag GmbH & Co. KGaA, Weinheim.

Chirality in regioregular **1** and regiorandom **2** poly [3-(*S*)-3′,7′-dimethyloctyl)thiophenes] was demonstrated and compared via circular dichroism (CD) [18] (Figure 2). CD enabled tracking of main-chain structural changes during micro-aggregation, distinguishing the ordered aggregated state from the random-coil structure in a good solvent. Furthermore, the bisignate pattern of the Cotton effect reveals the supramolecular helical handedness of both polymers.

**Figure 3:**
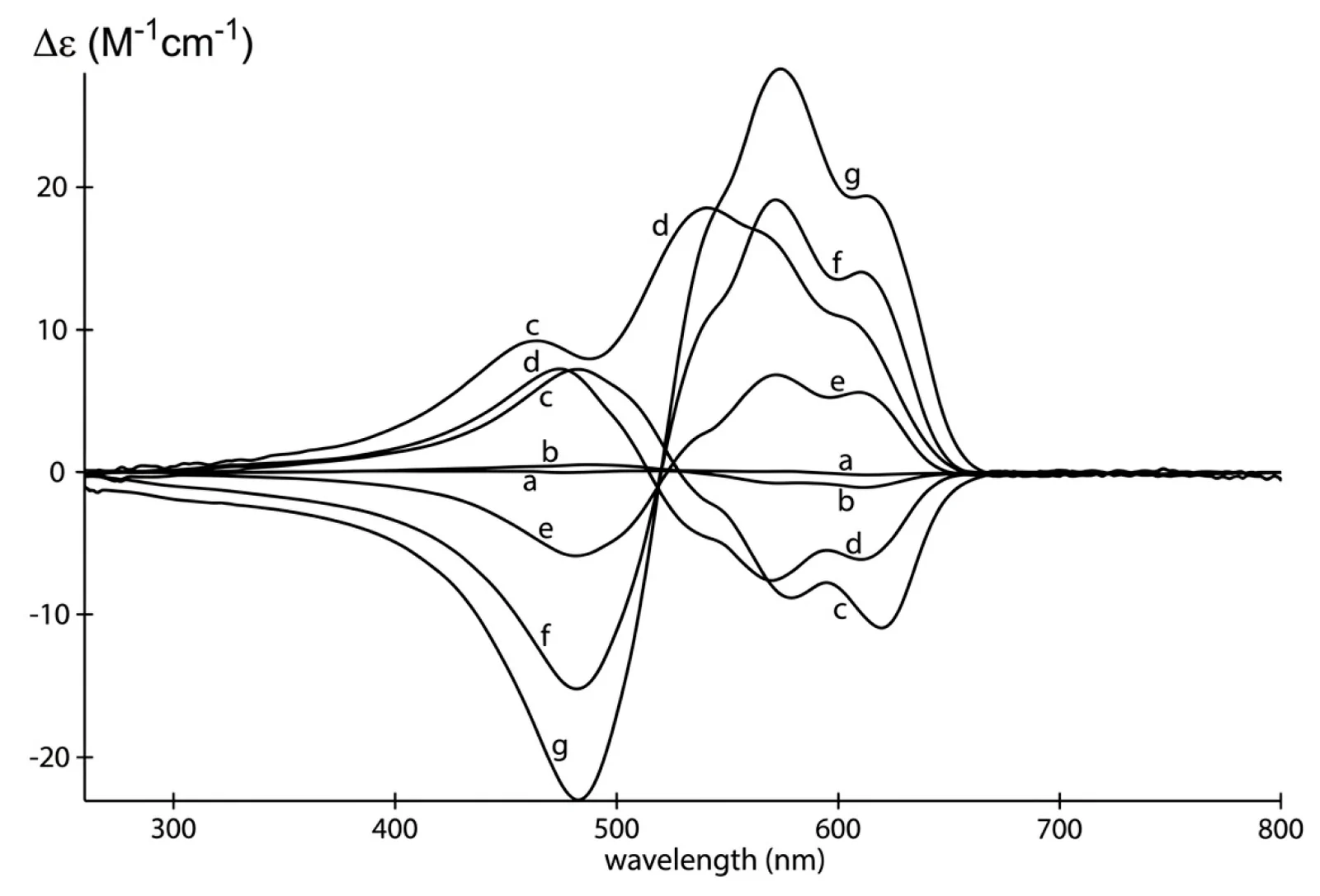
CD spectra of **1** in chloroform/methanol binary solvent system at ratios (*v*/*v*) (a) 100/0, (b) 65/35, (c) 62.5/37.5, (d) 60/40, (e) 57.5/42.5, (f) 55/45, (g) 50/50. Reprinted with permission from Bidan, G.; Guillerez, S. and Sorokin, V. (1996), Chirality in regioregular and soluble polythiophene: An internal probe of conformational changes induced by minute solvation variation ^†^. Adv. Mater., 8: 157–160. https://doi.org/10.1002/adma.19960080213 [18]. Published by John Wiley and Sons Copyright © 1996 Verlag GmbH & Co. KGaA, Weinheim.

In binary solvent systems, the supramolecular architecture was examined via CD spectra of regioregular polythiophene **1**. The results showed a two-step aggregation through two ordered conformations (intermediate state unassigned), accompanied by a reversal of the CD spectrum across a narrow non-solvent window (40–42.5% MeOH, Figure 3, spectra d and e).

Ikai et al. [19] reported that D-glucose-bearing polymer **3** adopts a stable, single-handed macromolecular helix in solution rather than undergoing intermolecular aggregation. This intramolecular origin is evidenced by concentration-independent CD spectra (C = 0.01–1.0 mM in chloroform). Furthermore, neither the absorbance nor the CD signal intensity changed after dense membrane filtration. 

**Figure 4:**
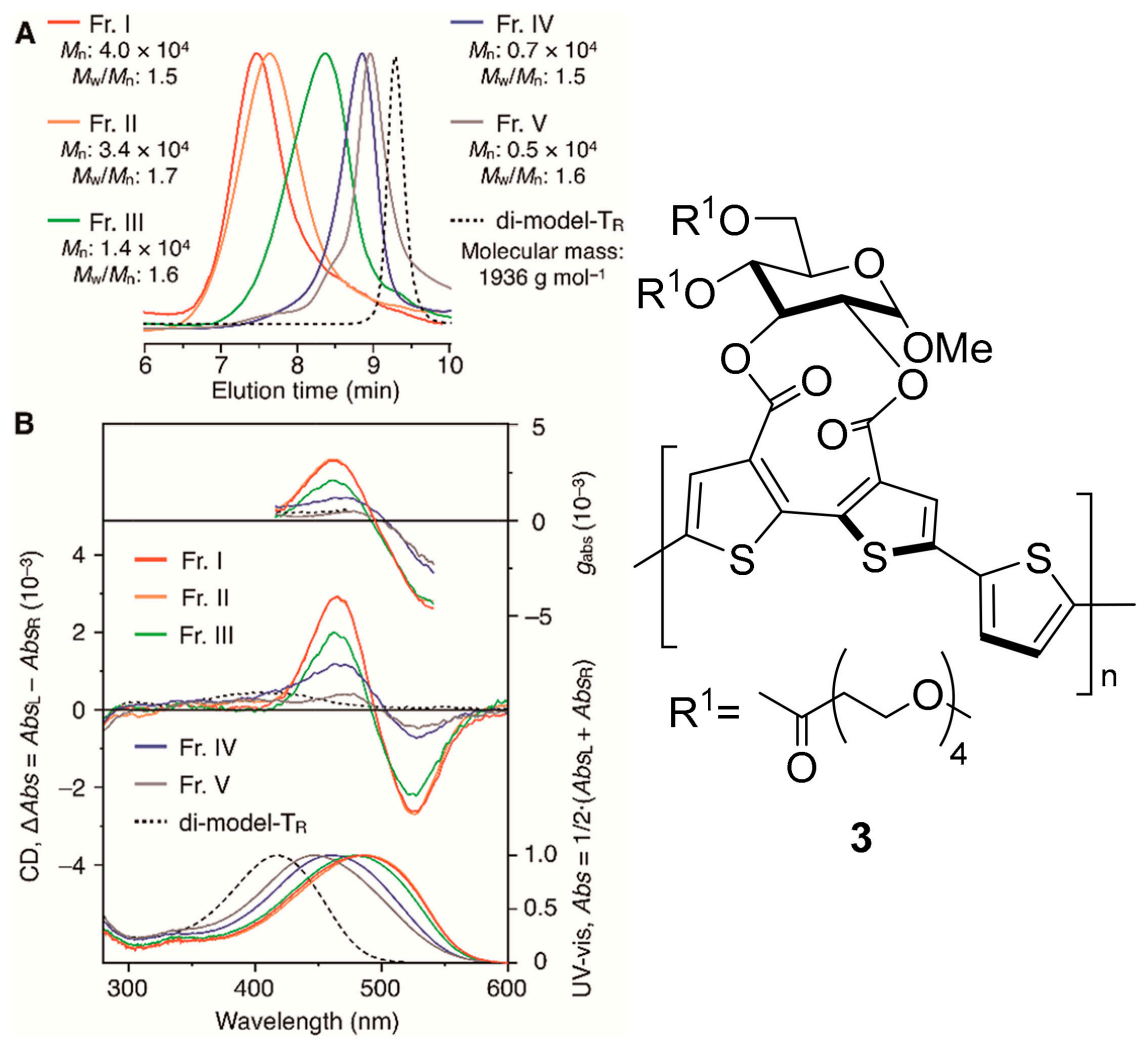
(**A**) SEC traces of fractionated components of **3** with different molecular masses (eluent, chloroform; polystyrene standards; di-model-T_R_ states for dibromoquinquethiophene decorated with two sugar moieties). (**B**) Molecular mass dependence of the CD and absorption spectra of **3** in chloroform at 25 °C. Used with permission under a Creative Commons Attribution 3.0 Unported Licence from: Tomoyuki Ikai, Kokoro Takayama, Yuya Wada, Serena Minami, Chanokporn Apiboon, Ken-ichi Shinohara; Synthesis of a one-handed helical polythiophene: a new approach using an axially chiral bithiophene with a fixed *syn*-conformation. *Chem. Sci.* 2019; 10 (18): 4890–4895. https://doi.org/10.1039/c9sc00342h [19]. Copyright© 2019 The Author(s). Published by the Royal Society of Chemistry.

The other dependence on CD response was observed as CD intensity increased with molecular weight, plateauing at Mn < 3.4 × 104 g/mol (Figure 4). This molecular-weight dependence of the chiroptical properties is characteristic of a single-handed macromolecular helical conformation. These findings provide further evidence that the **3** backbone adopts a stable secondary structure in solution. Strong CD signals originating from the helical chirality of **3** were observed across good solvents such as THF and chlorobenzene.

#### 2.2.2. Circularly Polarized Luminescence

Polymer **3** exhibited orange photoluminescence (PL) in both solution and solid states, originating from its conjugated backbone with a fluorescence quantum yield of 5% in CHCl_3_. [19]. Its macromolecular helical chirality induced right-handed circularly polarized luminescence (CPL), yielding a maximum luminescence dissymmetry factor |*g*_lum_| of 1.6 × 10^−3^ in chloroform and a significantly higher maximum luminescence dissymmetry factor *g*_lum_ = 6.9 × 10^−3^ in the drop-cast thin film. 

Furthermore, the CPL signal intensity of **3** was superior to that of d-glucose-linked quaterthiophene, thus demonstrating that the helical conformation of the polymer backbone is of key importance for achieving enhanced CPL activity.

**Figure 5:**
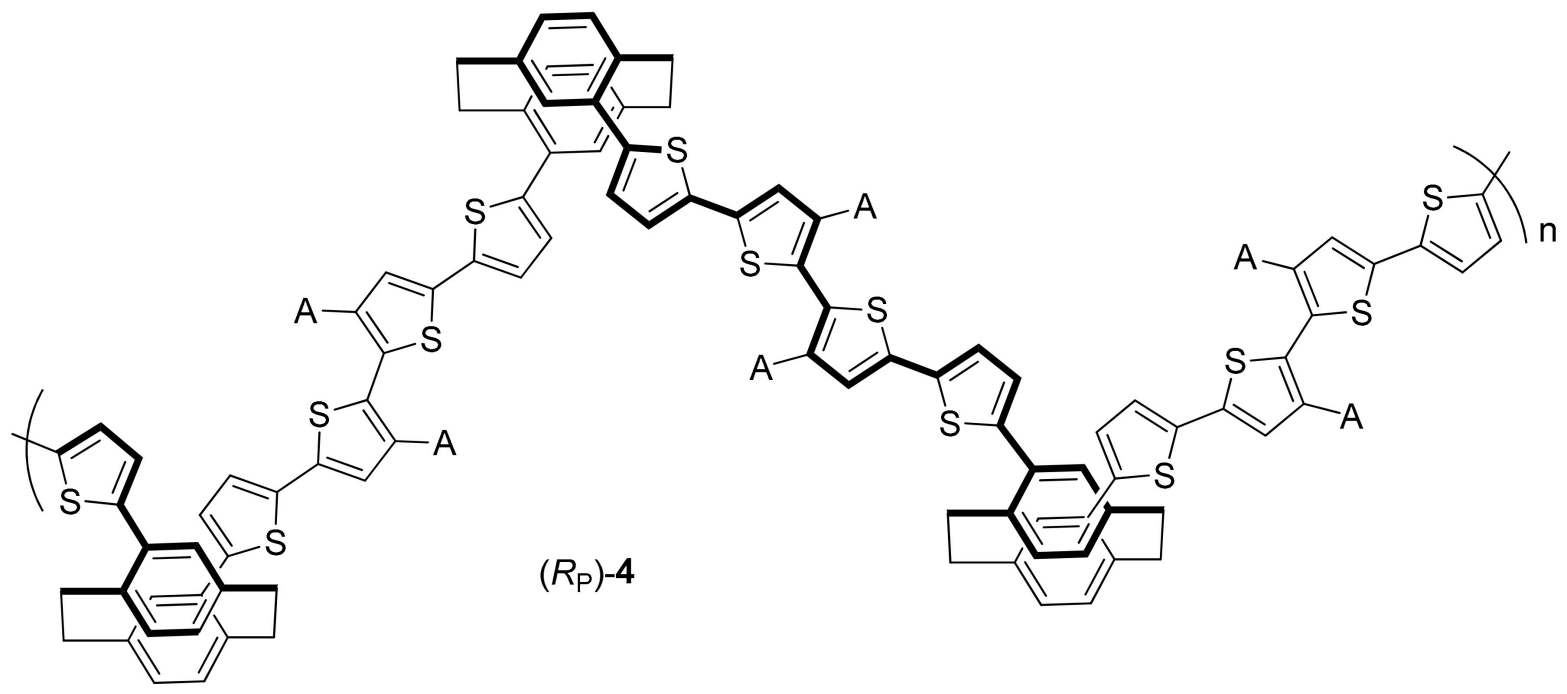
[2.2]Paracyclophane–based conjugated polymer.

The optically active, through-space-conjugated polymers (*R*p)-**4** (Figure 5) and its (*S*p) enantiomer emitted green photoluminescence in CHCl_3_ solution and exhibited mirror-image signals in their CPL spectra [20]. The authors noted that because CPL signals from optically active polymers typically appear only in films or aggregates, observing a clear CPL signal for a monodisperse polymer in solution is rare. Furthermore, they concluded that the arrangement of the [2.2]paracyclophane–based conjugated polymer is driven by the stacked π-electron network, where each stacked 2,2′′′-dixylyl-2,2′:5′,2′′:5′′,2′′′-quaterthiophene unit adopts planar/quinoid conformations in the excited state.

#### 2.2.3. Other Structure Characterization Methods 

Beyond chiroptical methods, techniques such as NMR spectroscopy, light scattering, X-ray diffraction (SAXS, GIWAXS), and microscopy (AFM, FE-SEM, POM) characterize the structure, stereochemistry, and morphology of complex supramolecular assemblies. Integrating these tools helps establish key structure–property relationships. Despite remarkable advances in synthesis and characterization, the relationship between molecular design and supramolecular organization, such as the principles of aggregation processes or chirality transfer, remains only partially understood.

### 2.3. Methods/Strategies for Introducing Chirality into Polythiophenes

Chirality is introduced into polythiophenes through several synthetic and physical strategies. These include covalent modification via the attachment of chiral side chains prior to polymerization (a topic detailed in Section 3, “Synthesis and Structural Determination of Non-Racemic Polythiophenes”), as well as supramolecular assembly routes. Supramolecular chirality-based methods cover the non-covalent transfer of chiral information, external field induction using chiral solvents, liquid-crystalline media, or chiral dopants, and physical stimuli such as circularly polarized light (CPL) (Examples of chirality transfer as an alternative approach for chirality induction are completed in Section 3.4). Furthermore, asymmetric electropolymerization in chiral templates such as cholesteric (chiral nematic, N*-LC) reaction media provides a direct pathway to chiral materials, as briefly discussed below within the scope of this review.

### 2.4. Induction of Chirality in Liquid Crystals (LCs)

**Figure 6:**
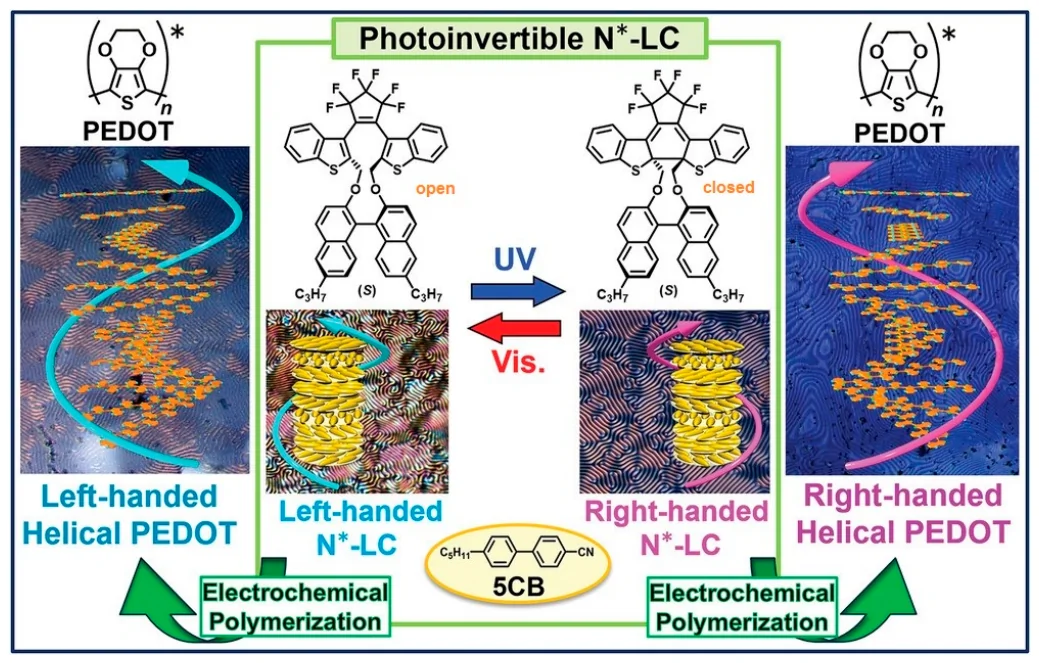
Induction of helicity in poly(3,4-ethylenedioxythiophene)s (H-PEDOTs) during asymmetric electrochemical polymerization of EDOT within a chiral nematic liquid crystal (N*-LC) matrix containing photoresponsive chiral dopants. Reprinted with permission from S. Yoshida, S. Morikawa, K. Ueda, M. Hidaka, K. Kaneko, K. Kaneko, T. Hanasaki, K. Akagi, Photoinvertible Chiral Liquid Crystal that Affords Helicity-Controlled Aromatic Conjugated Polymers. *Adv. Optical Mater.* 2020, 8, 2000936. https://doi.org/10.1002/adom.202000936 [22]. Published by John Wiley and Sons © 2020 Wiley-VCH GmbH.

Chiral liquid crystals (LCs) can serve as templating reaction fields to direct the macroscopic helicity of conjugated polymers during polymerization. Thermally invertible chiral LCs have been used for interfacial chemical polymerizations yielding helical polyacetylene films [21], whereas a photoinvertible chiral LC (N*-LC) was applied for the electrochemical synthesis of helical PEDOT [22]. This fatigue-resistant, photoinvertible N*-LC, was obtained by doping 5CB with a compound combining a photoresponsive bisbenzothienylethene core and axially chiral binaphthyl groups, imparting high helical twisting power (HTP). Reversible photoisomerization of the bisbenzothienylethene moiety between its open and photostationary state (PSS) closed forms flips the handedness of the LC matrix. Electrochemical polymerization of 3,4-ethylenedioxythiophene (EDOT) within this dynamic asymmetric field yields helical PEDOT (H-PEDOT) (Figure 6) with a direction of helicity transcribed from the LC matrix. 

### 2.5. Aggregations Leading to Optical Activities

**Figure 7:**
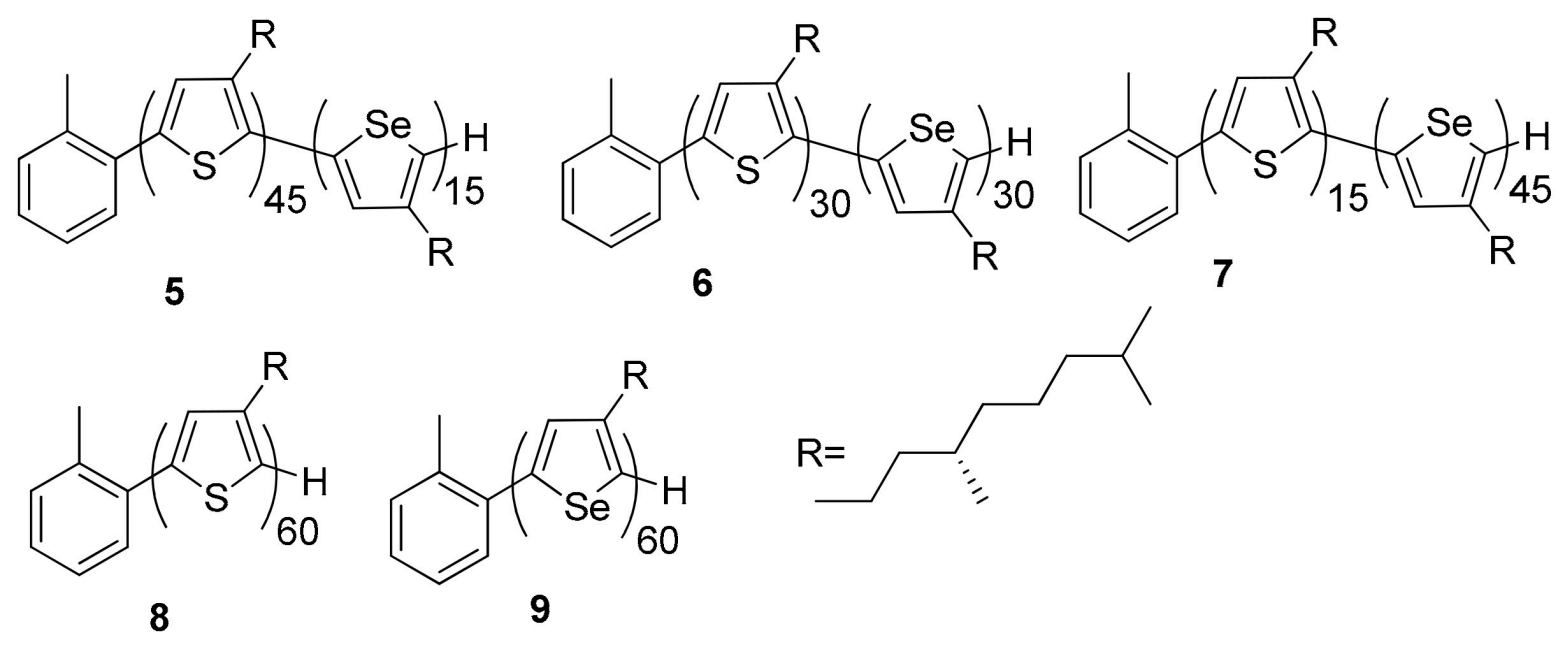
Block copolymer **5**–**7** (**top**) and homopolymers of poly(thiophene) **8** and poly(selenophene) derivatives **9** (**bottom**).

Self-assembly and aggregation govern the optoelectronic properties of π-conjugated polymers, as demonstrated by studies on three co-crystallizing chiral block copolymers **5**–**7** containing poly((*S*)-3-(3,7-dimethyloctyl)thiophene) and poly((*S*)-3-(3,7-dimethyloctyl)selenophene) blocks (Figure 7) [23]. Copolymers **5**–**7** and polythiophene homopolymer **8** undergo chiral self-assembly, whereas polyselenophene homopolymer **9** does not self-assemble in a chiral way due to strong Se–Se interactions. In the block copolymers, co-crystallization leads to partial segment mixing within the crystalline structure, replacing strong Se–Se interactions with weaker Se–S interactions to unlock chiral order. These copolymers exhibited a stronger chiral response than the corresponding homopolymers, which is attributed to a higher degree of disorder that promotes helical twisting. Spectroscopic studies (UV–Vis, CD) and DSC confirmed co-crystallization, while dynamic light scattering (DLS) indicated that the optical activity depends on molecular organization rather than aggregate size. 

**Figure 8:**
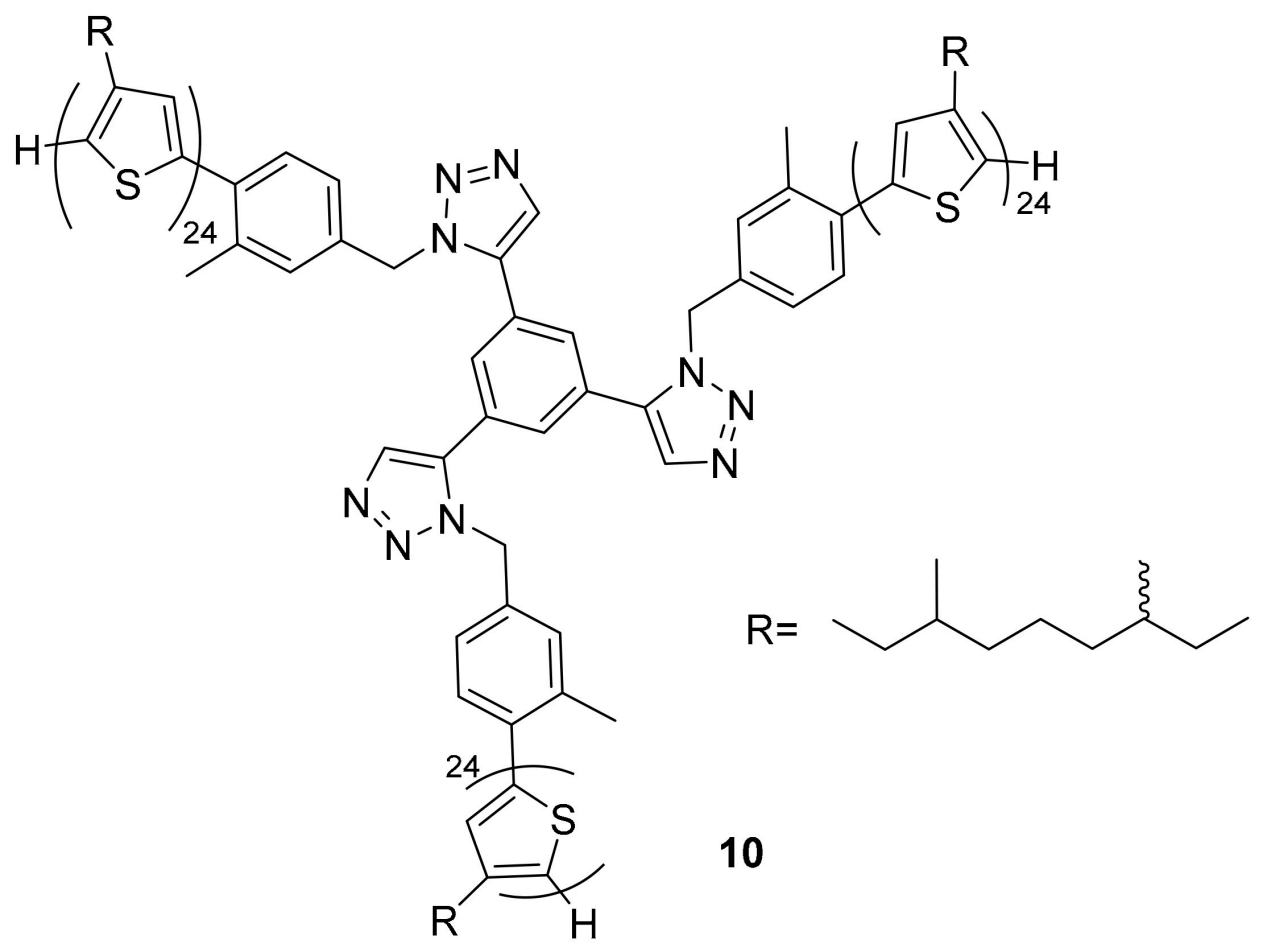
Structure of star-shaped P3AT (**10**) [24,25].

The macromolecular structure of poly(3-alkylthiophenes) (P3ATs) also plays a key role in governing their supramolecular organization. Unlike linear analogues, star-shaped P3ATs (Figure 8), form more ordered structures with stronger chiroptical effects [24]. Enhancement of optical activity is manifested by a strong Cotton effect and a significant increase in the dissymmetry factor (*g*-factor) up to two orders of magnitude compared to linear analogue. Detailed mechanistic studies by two research groups further clarify the supramolecular assembly of these star-shaped systems [24,25].

Importantly, this effect does not arise from increased electronic coupling between the arms, but from cooperative supramolecular organization [25]. The self-assembly of **10** may follow two different routes. In a solvent/nonsolvent system, aggregation proceeds via a multistep process starting with solvent-separated prenucleation clusters that lack electronic coupling (confirmed by CD and UV–Vis). Subsequently, with an increasing fraction of nonsolvent, partial chain planarization and increase in conjugation length occur, leading to highly ordered supramolecular structures with strong π → π* stacking interactions. In contrast, thermally induced aggregation proceeds in a single step at a critical temperature where simultaneous chain planarization and π → π* stacking occur, resulting in aggregates with greater size dispersity and less structural order. 

Further insight into the mechanism of chiral amplification in these systems was provided by studies on heterogeneous star-shaped conjugated polymers [25]. Three possible scenarios were considered: a Sergeant-and-Soldiers-like effect, a “defect effect”, and hampered supramolecular organization. UV–Vis, CD, DSC, and AFM studies revealed that the heterogeneous systems formed less ordered aggregates and exhibited a significantly weaker chiroptical response than the fully chiral, symmetric star-shaped analogues. Notably, the chiroptical response decreased more strongly than would be expected solely from the reduced number of chiral arms, thereby excluding both the Sergeant-and-Soldiers-like mechanism and the “defect effect”. The results further indicate that principles established for linear P3ATs cannot be directly transferred to more complex star-shaped architectures and that a high degree of symmetry and structural homogeneity is essential for achieving strong chiroptical activity.

**Figure 9:**
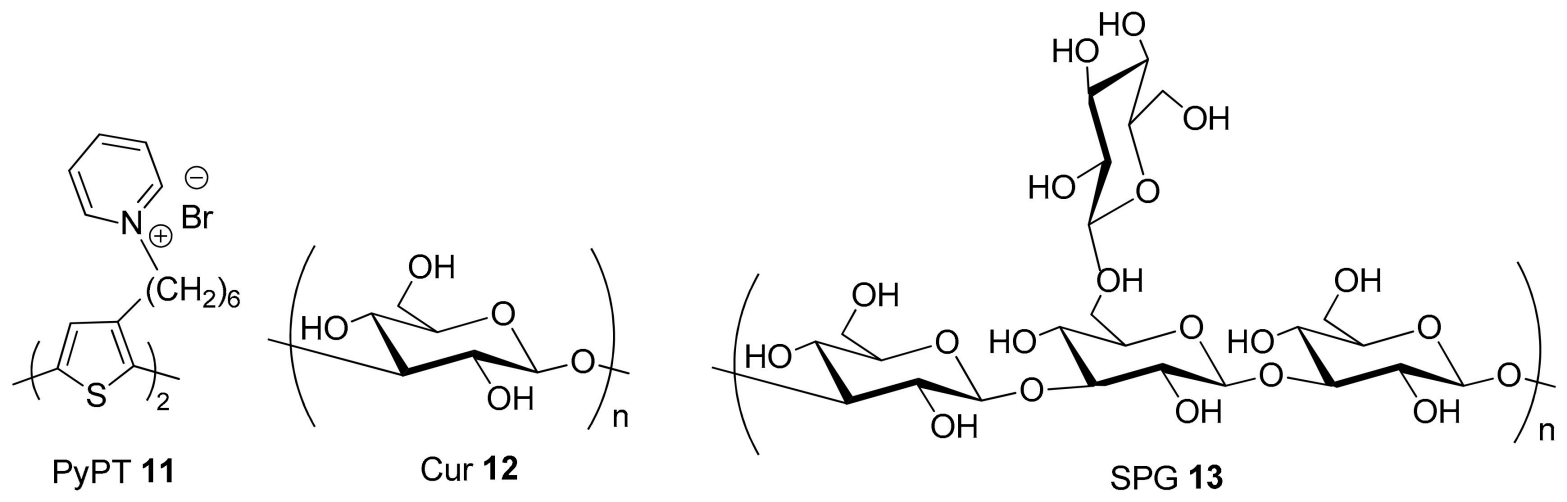
Structures of poly(3-(6-pyridiniohexyl)thiophene (PyPT) **11**, curdlan (Cur) **12** and schizophyllan (SPG) **13** [26].

**Figure 10:**
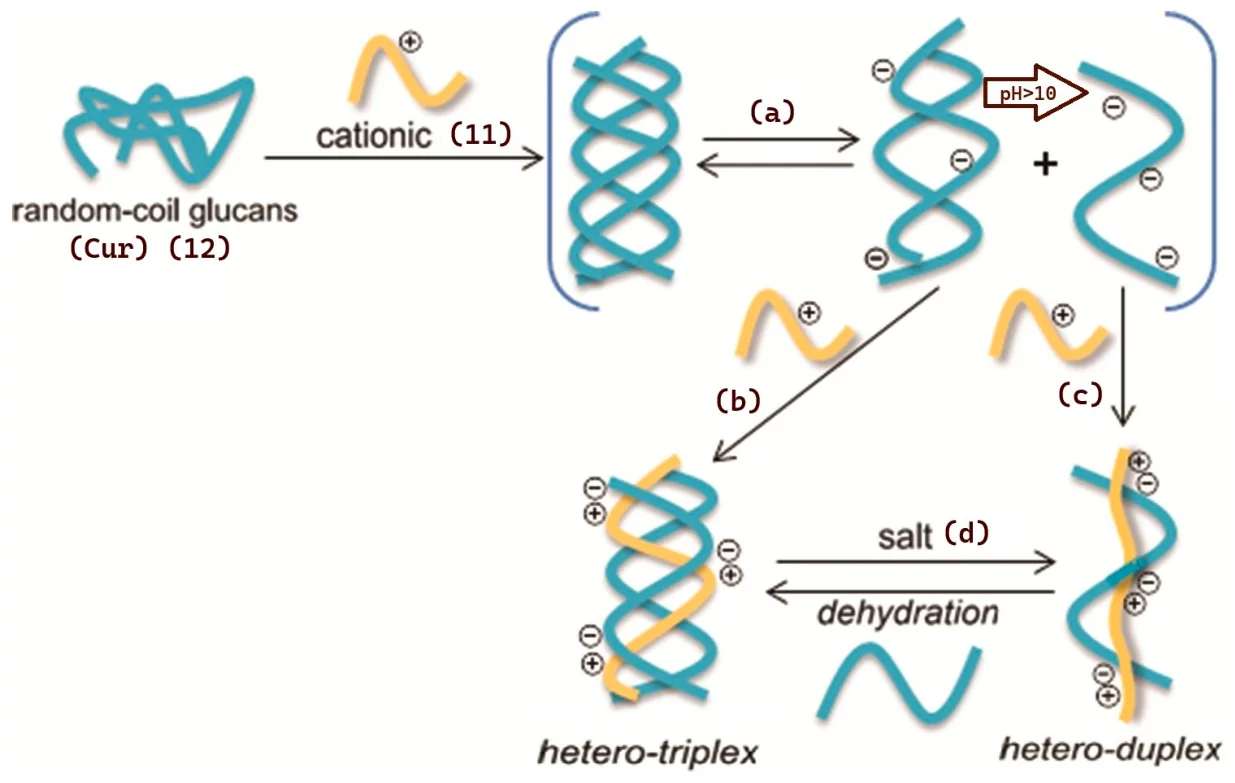
The mechanism of the hybridization of PyPT and Cur, (a) the equilibrium of the homo-triplex with a pair of single and double strands of Cur; (b) The hetero-triplex of anionic double strands of Cur with a cationic PyPT; (c) a loosely bound hetero-duplex of a cationic PyPT and an anionic Cur strand; (d) the equilibrium of hetero-triplex and duplex consisted of a polythiophene and Cur chains. Adopted with permission from Electrostatically promoted dynamic hybridization of glucans with cationic polythiophene *Org. Biomol. Chem*. 2016; 14 (41), 9741–9750. https://doi.org/10.1039/c6ob01353h [26]. © 2016 The Author(s). Published by the Royal Society of Chemistry.

Aggregation of cationic polythiophene **11** and *β*-1,3-glucan polysaccharide chains of curdlan (Cur) **12** or schizophyllan (SPG) **13**, driven by electrostatic interactions, leads to highly ordered supramolecular structures with pronounced optical activity (Figure 9) [26]. This assembly occurs via a hybridization mechanism that leads to the formation of hetero-triplex and hetero-duplex structures (Figure 10). In hetero-triplexes, two glucan chains stabilize one polythiophene chain, forming a helical complex with defined handedness. This structure imposes a helical conformation on the π-conjugated backbone, resulting in strong CD signals, even though the polythiophene itself is intrinsically achiral. Elevated temperatures or alkaline environments deprotonate glucan hydroxyl groups, driving homo-triplex dissociation and reorganization into more stable hetero-complexes with enhanced optical response. At high pH, a transition from hetero-triplex to hetero-duplex occurs relaxing the helical structure and altering conjugation length while maintaining persistent induced chirality. 

## 3. Synthesis and Structural Determination of Non-Racemic Polythiophenes

Several methods have been developed for preparing optically active polymers [1].

a. Polymerization of Optically Active Monomers Bearing Chiral Substituents [27]

Chiral polythiophenes (PThs), especially those with an optically active substituent at the 3-position, are widely studied. Beyond the chiral centers of the side-chain substituents, optical activity can manifest during the π → π* transition, resulting in chirality within the main polymer chain. The stereogenic side chains induce intramolecular helical order within aggregates or in the solid state, translating side-chain chirality into main-chain optical activity during π → π* transition.

b. Asymmetric Polymerization and Chirality Transfer [28,29]

In asymmetric variant of polymerization, prochiral monomer (either optically inactive or bearing a chiral auxiliary) is polymerized, to yield polymer with a defined backbone configuration or conformation, creating main-chain chirality.

Chirality transfer occurs during the electrochemical or chemical polymerization of achiral monomers when performed within a chiral matrix (e.g., a chiral liquid-crystal medium; Section 3.2.2), thereby transferring the matrix chirality into the polymer backbone.

c. Post-polymerization Chirality Induction in Achiral PThs

Induction of chirality after polymerization is achieved by introducing a chiral guest that binds to an optically inactive polymeric structure. For instance, an optically inactive polythiophene becomes chiral upon complexation with a chiral guest via acid–base interactions. This induction forces the conjugated main chain of the polymer to adopt a predominantly single-handed helical conformation [30,31].

Another example involves mixing achiral polythiophenes with chiral biomolecules, such as peptides or DNA [32,33], which impart their helical superstructures to the polymer. Driven primarily by electrostatic interactions, the polythiophene chains adopt a helical configuration with a preferred handedness dictated by the biomolecule, leading to the manifestation of supramolecular chirality in otherwise achiral PThs. 

Similarly, achiral polythiophene bearing oxazoline pendant groups form optically active, metal-induced supramolecular aggregates upon complexation with chiral amines or in the presence of a chiral polythiophene, exhibiting supramolecular chirality even in good solvents (as confirmed by CD spectroscopy) [34].

### 3.1. The Synthetic Approaches Based on the Chemical Polymerizations

The majority of studies on chiral polythiophenes (PThs) focus on derivatives with enantiomerically pure side chains, which induce main-chain chirality or promote the formation of helical aggregates. Investigating diverse chiral substituents helps clarify how molecular and supramolecular structure governs overall physicochemical properties. The following two subsections discuss the development of chemical polymerization methods for regioregular and regiorandom 3-substituted PThs, (including foundational studies to better illustrate overall synthetic progress).

#### 3.1.1. Synthetic Methods for Regioregular Chiral Polythiophenes

a. McCullough and Rieke Method

Synthetic approaches enabling regioregular head-to-tail (HT) coupling are highly sought after, as this regiochemistry imparts superior optical, electrochemical, and material properties. The evolution of these synthetic strategies is provided here in a brief historical overview.

**Scheme 1:**
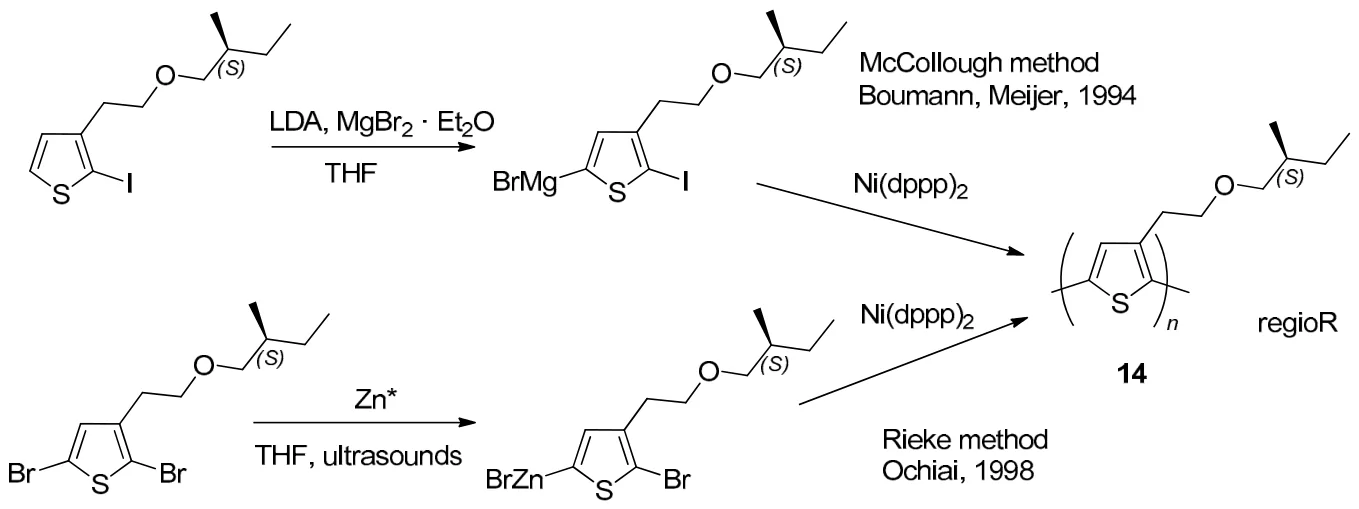
Synthesis of chiral regioregular polythiophene (PThs) **14**.

The pioneering synthesis of chiral rrPThs (**14**, Scheme 1) was reported by Meijer in 1994 utilizing the McCullough protocol, which relies on a Ni-catalyzed cross-coupling polymerization of a 3-substituted thiophene monomer bearing an enantiomerically pure alkoxyethyl side chain [35]. 

**Figure 11:**
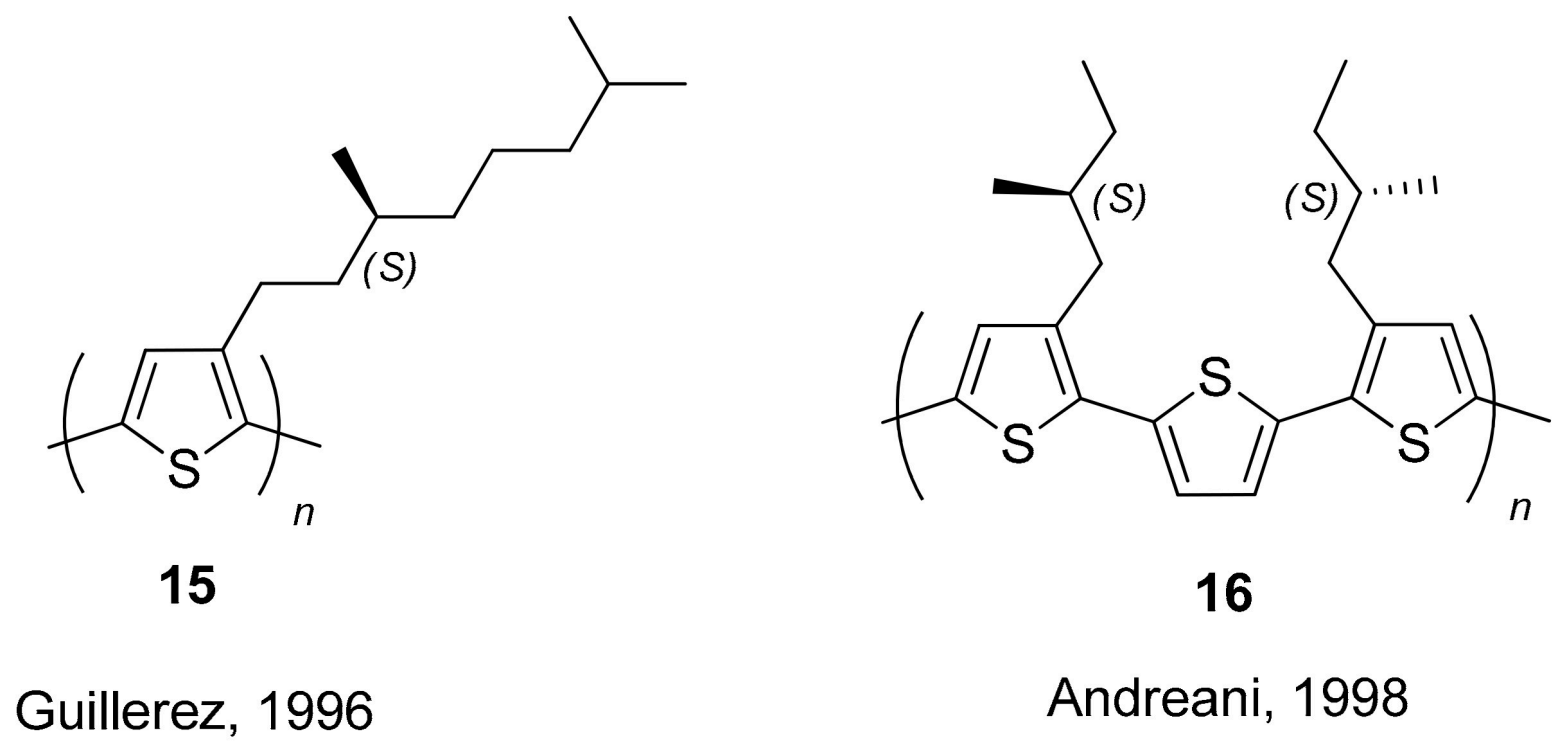
Polythiophenes **18** and **19**.

In 1995, Meijer and Bouman reported that this polymer **14** undergoes stereomutation under rapid cooling conditions, resulting in a complete conformational reversion and a switch in the screw sense of the main chain while preserving the absolute configuration of its side-chain stereocenters [16]. In 1998, Ochiai and coworkers synthesized highly regioregular head-to-tail poly(3-[2-((*S*)-2-methylbutoxy)ethyl]thiophene) (**14**) utilizing a modified Rieke protocol (Scheme 1) [36,37]. Using the McCullough procedure, Guillerez and coworkers [18] synthesized poly(3-((*S*)-3′,7′-dimethyloctyl)thiophene) (**15**) (Figure 11), achieving 100% of HT couplings as determined by NMR spectroscopy.

b. Chemical Oxidative Polymerization

**Scheme 2:**
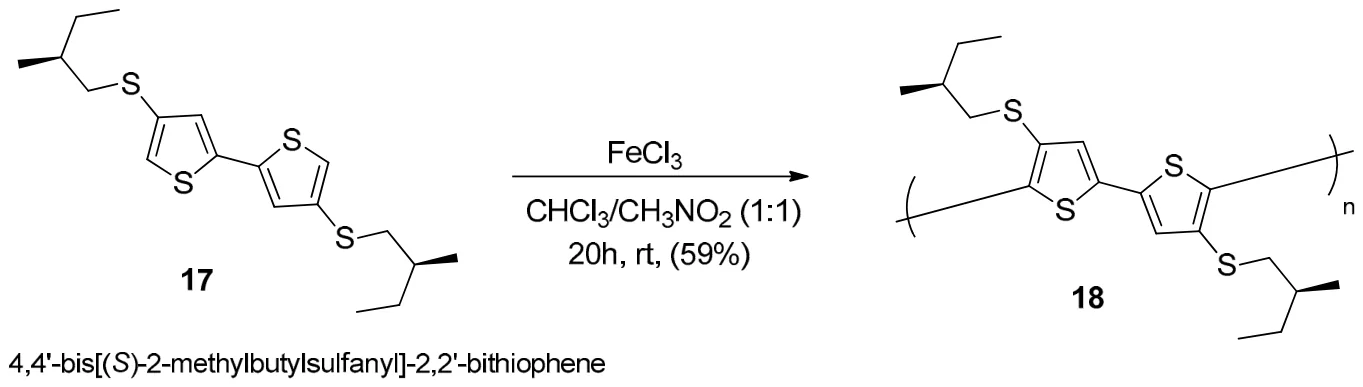
Synthesis of regiosymmetric disubstituted poly(bithiophene) **18**.

Andreani and coworkers [38] synthesized a chiral regiosymmetric polythiophene **16** from the centrosymmetric monomer 3,3″-di[(*S*)-(+)-2-methylbutyl]-2,2′:5′,2″-terthiophene via oxidative chemical polymerization with FeCl_3_ according to the Sugimoto protocol (Figure 11). As sulfur-containing side-chain representatives, poly(3-alkanesulfanylthiophene)s were prepared either chemically or electrochemically from 3-alkanesulfanyl monothiophene or β,β′-dithialkyl bithiophene precursors. For instance, tail-to-tail (TT) precursor, (+)-4,4′-bis[(*S*)-2-methylbutylsulfanyl]-2,2′-bithiophene **17**, underwent FeCl_3_-mediated oxidative polymerization to yield regioregular poly(β,β′-disubstituted bithiophene) **18** (Scheme 2) [39]. 

Structural characterization via ^1^H NMR spectroscopy revealed a single aromatic resonance at 7.19 ppm, confirming the head-to-head/tail-to-tail (HH/TT) regiochemistry. UV-Vis and CD studies of **18** in various solvent mixtures demonstrated pronounced solvatochromic behavior and chiral supramolecular induction. In chloroform, **18** exists in a disordered, CD-inactive state with an absorption maximum at 469 nm (π → π* transition). Adding poor solvents (methanol or *n*-hexane) triggers chiral aggregation in reflected by UV-Vis and CD responses as an orange-to-violet color change, a red-shifted absorption spectrum with vibronic peaks at 530, 565, and 623 nm, and a bisignate Cotton effect in the absorption and circular dichroism spectra. 

**Scheme 3:**
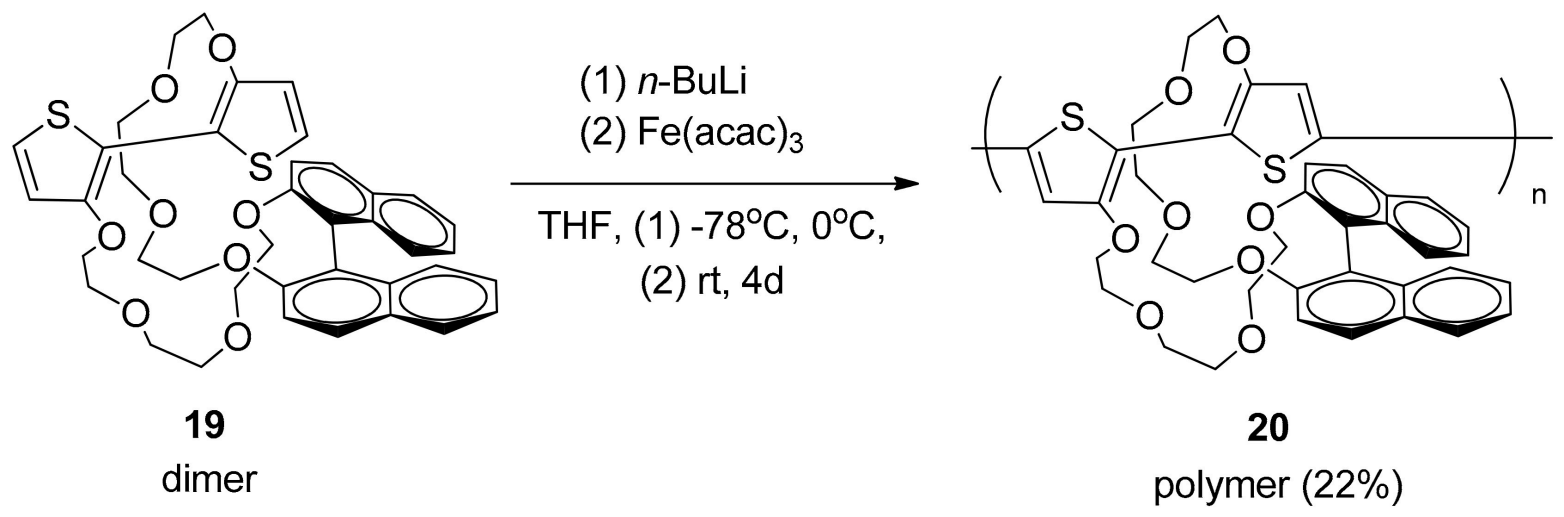
The polymerization of bithiophene with binaphthyl-ether crown-appended pendants.

Separately, optically active poly(bithiophene) **20**, incorporating a crown ether linker composed of oxyethylene and binaphthyl motifs, was synthesized in 22% yield via the [Fe(acac)_3_]-mediated oxidative coupling of a head-to-head (HH)-configured dimer **19** (Scheme 3) [40]. 

Full structural characterization of polythiophene **20** via ^1^H and ^13^C NMR confirmed selective polymerization at the reactive α-positions of the bithiophene unit. Gel permeation chromatography (GPC) determined a number-average molecular weight (M_n_) of 3400 and polydispersity index (PDI) of 1.6. Notably, aa additional higher-molecular-weight fraction eluting above the upper exclusion limit of the GPC column was attributed to self-assembled aggregates formed through strong interchain interactions. Furthermore, polythiophene **20** exhibited chiroptical sensing capabilities toward chiral compounds upon host–guest inclusion, which can be monitored using UV-Vis absorption spectroscopy (Section 4.2).

c. Direct Arylation Polymerization (DArP)

**Scheme 4:**
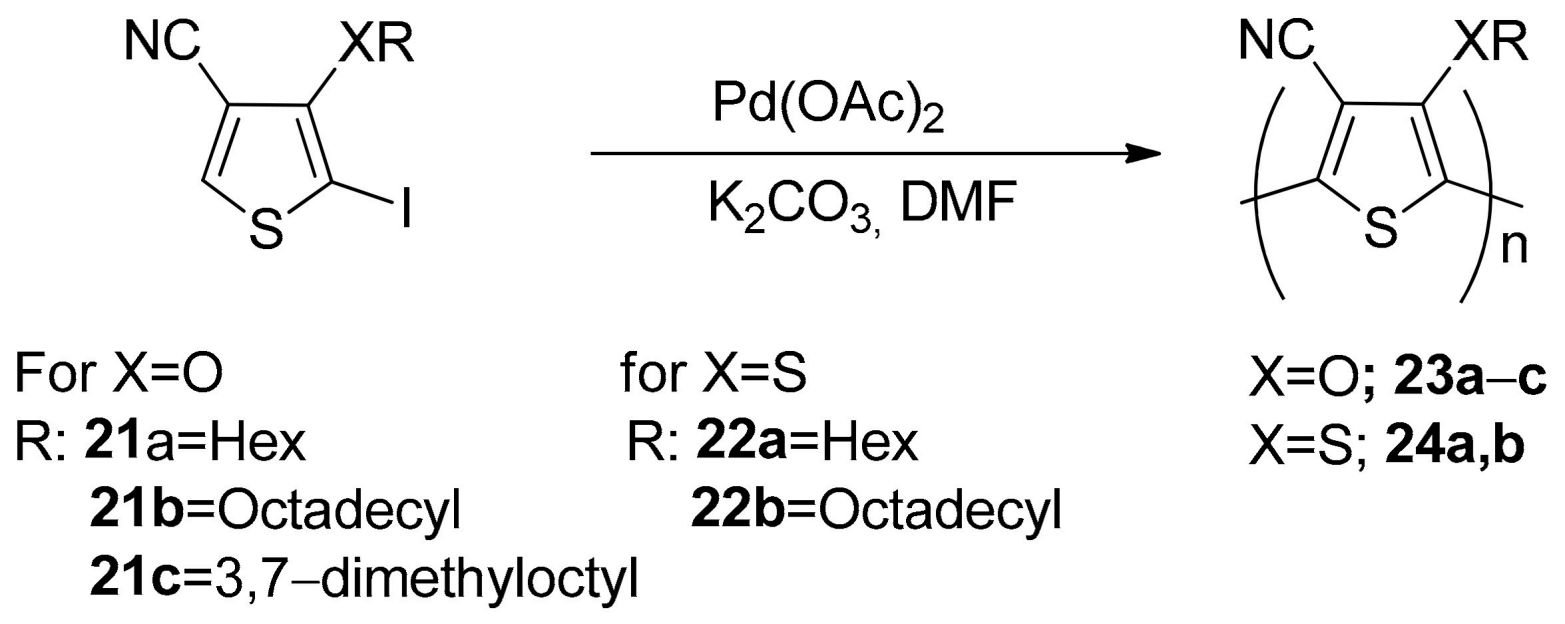
Synthesis of poly(3-alkoxy(alkylsulfanyl)-4-cyano)thiophenes **26**–**27**.

Direct arylation polymerization (DArP) is an alternative, atom-economical synthetic approach to π-conjugated polymers via dehydrohalogenative coupling based on palladium-catalyzed C–H bond activation. Although this reaction manifold has been known since the early 1980s, its application to macromolecular synthesis was realized only recently. Conventional catalytic systems typically combine Pd(OAc)_2_ paired with a base such as K_2_CO_3_ and polar solvents like *N*,*N*-dimethylacetamide (DMA) or *N*,*N*-dimethylformamide (DMF). In some cases, phosphine ligands are employed. With this approach, poly(3-alkoxy(alkylsulfanyl)-4-cyano)thiophenes **23**, **24** were synthesized via direct arylation polymerization utilizing K_2_CO_3_ (3 equiv.) and Pd(OAc)_2_ (10 mol%) in DMF (Scheme 4), pairing electron-donating alkoxy or alkylthio groups (**21**, **22**) with electron-withdrawing cyano units [41].

**Scheme 5:**
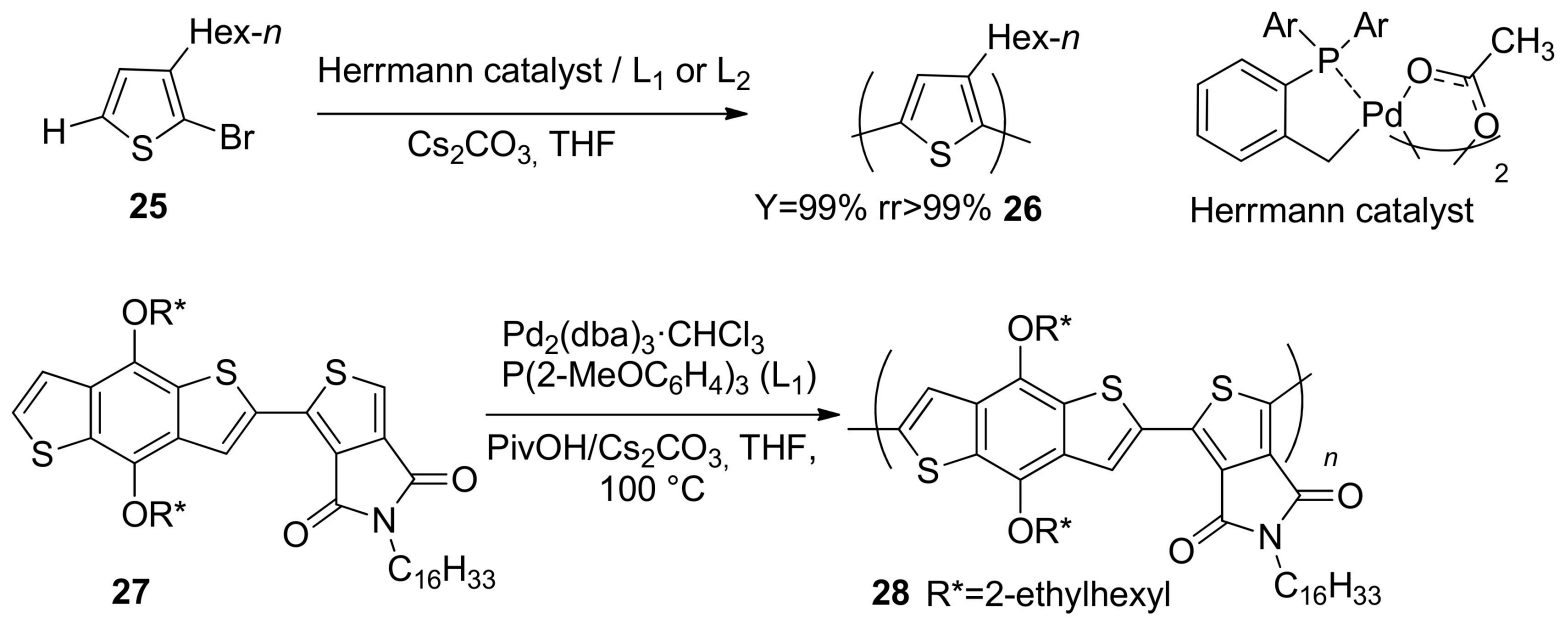
Synthesis of poly(3-hexylthiophene) **26** and copolymer **28**.

More recently, Wakioka and Ozawa advanced DArP protocols using palladium catalysts, pivalic acid (PivOH), Cs_2_CO_3_ and a bulky phosphine ligands (**L_1_** or **L_2_**); P(2-MeOC_6_H_4_)_3_ (**L_1_**) or P(2-Me_2_N-C_6_H_4_)_3_ (**L_2_**) [42,43,44]. This enabled the synthesis of highly head-to-tail (HT) regioregular poly(3-hexylthiophene) **26** from-bromo-3-hexylthiophene **25** with >99% regioregularity when employing Herrmann’s catalyst and ligand P(2-Me_2_NC_6_H_4_)_3_ (**L_2_**) (Scheme 5, top) [44]. Furthermore, this DArP methodology was extended to prepare donor–acceptor (D-A) copolymer **28** starting from **27** using a Pd_2_(dba)_3_·CHCl_3_/P(2-MeOC_6_H_4_)_3_ (**L_1_**)/PivOH/Cs_2_CO_3_ catalytic cocktail (Scheme 5, bottom).

d. Direct C-H Coupling Polymerization

**Scheme 6:**
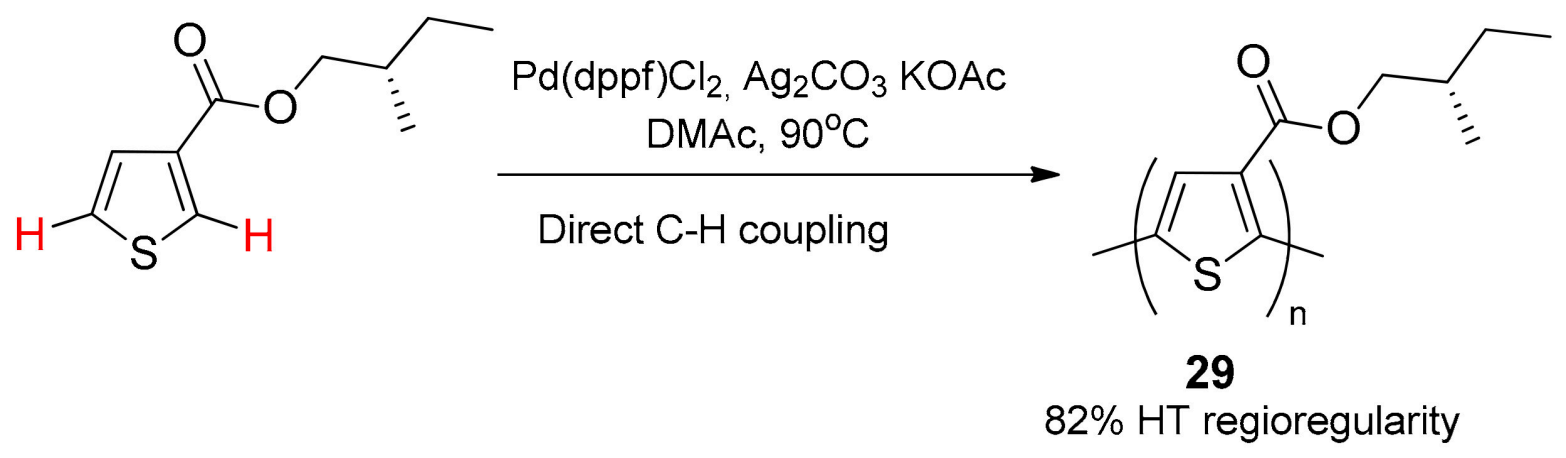
Synthesis of poly[(*S*)-2-methylbutyl thiophene-3-carboxylate] (**29**).

As a complementary synthesis strategy, direct C-H coupling polymerization offers an alternative pathway to π-conjugated systems, as exemplified by the synthesis of poly(3-substituted thiophene) **29** with high head-to-tail (HT) regioregularity (ca. 82%) conducted utilizing [Pd(dppf)Cl_2_] (0.05 equiv.) as the catalyst (Scheme 6) [45]. Introducing electron-withdrawing, alkyl ester substituents stabilizes the HOMO energy level, widening the bandgaps, to 2.54–2.66 eV compared to standard poly(3-alkylthiophenes).

e. Stille Polycondensation

**Scheme 7:**
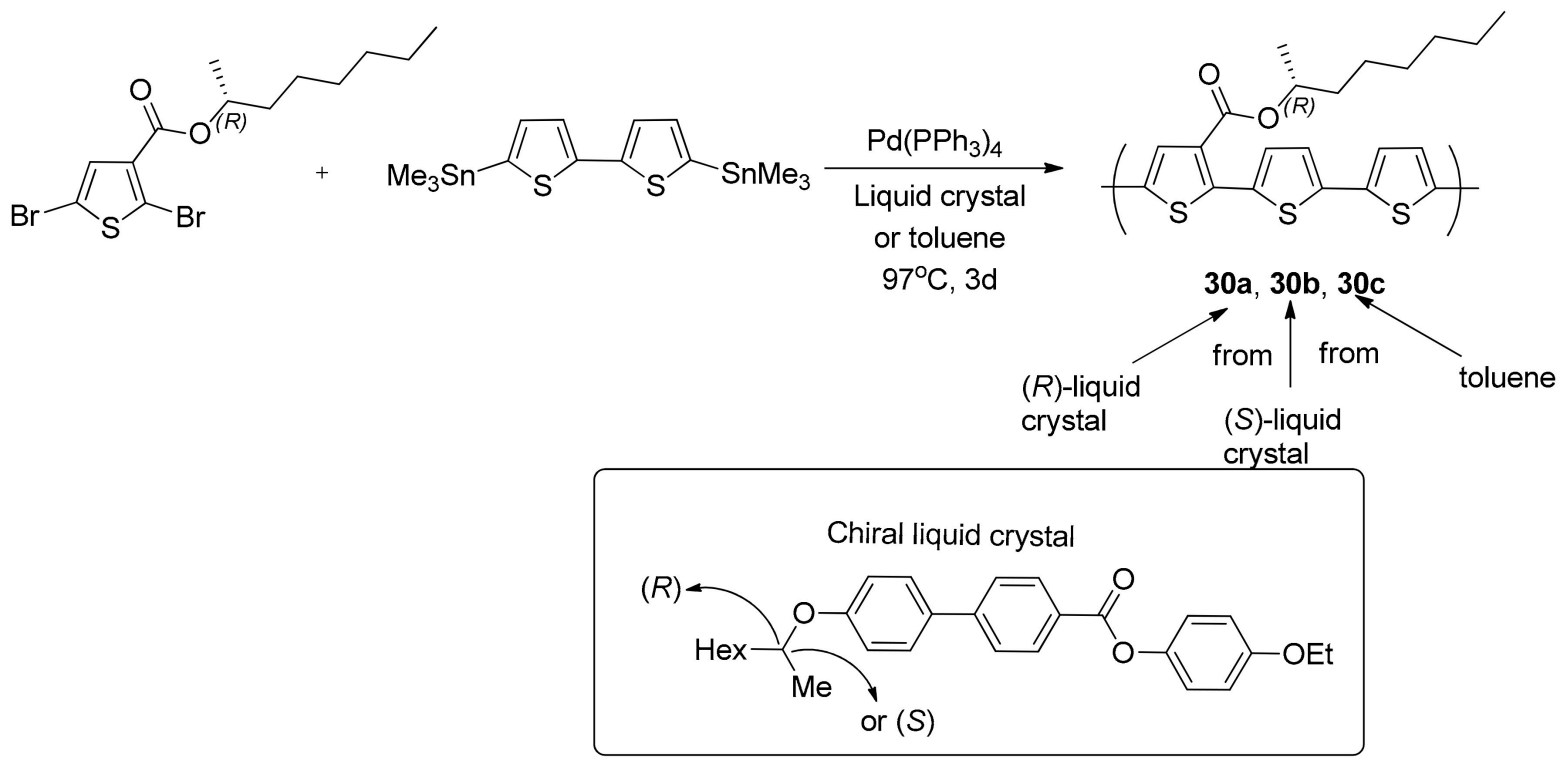
Stille polycondensation in various reaction media leading to poly(terthiophene) derivative **30**.

Among the diverse synthetic routes to head-to-tail (HT) regioregular polythiophenes, Migita–Kosugi–Stille cross-coupling polycondensation is a notable step-growth method [46]. Goto, Ohkawa, and co-workers applied this approach to synthesize novel chiral polythiophenes **30a**–**30c** by coupling (*R*)-octan-2-yl 2,5-dibromothiophene-3-carboxylate with 5,5′-bis(trimethylstannyl)-2,2′-bithiophene in both achiral toluene and a chiral liquid crystal (CLC) medium (Scheme 7) [47]. This procedure yielded a copolymer that adopts a supramolecular chiral conformation in an achiral solvent, driven by point stereogenic centers. Alternatively, when the polycondensation was conducted in a chiral medium, asymmetric induction was mediated directly by the solvent environment. Circular dichroism (CD) spectra of **30a**–**30c** in CHCl_3_ revealed a preferred main-chain twist, giving a positive Cotton effect at 400 nm and a negative one at 300 nm (absent in **30c**). Polymer **30c** showed chiroptical activity in the absence of a chiral medium, indicating that its macromolecular asymmetry arises from spontaneous aggregation driven by its chiral pendant group. Polymer **30b**, synthesized from the (*R*)-configured monomer within an (*S*)-configured CLC matrix, displayed significantly more intense CD signals than either **30a** or **30c**. Although polymer **30c** showed clear CD signals without a chiral medium, the enhanced optical activity of **30b** suggests a dual mechanism. Specifically, the structural chirality of **30b** arises from synergistic chirality transfer from the CLC host alongside spontaneous chiral aggregation.

**Scheme 8:**
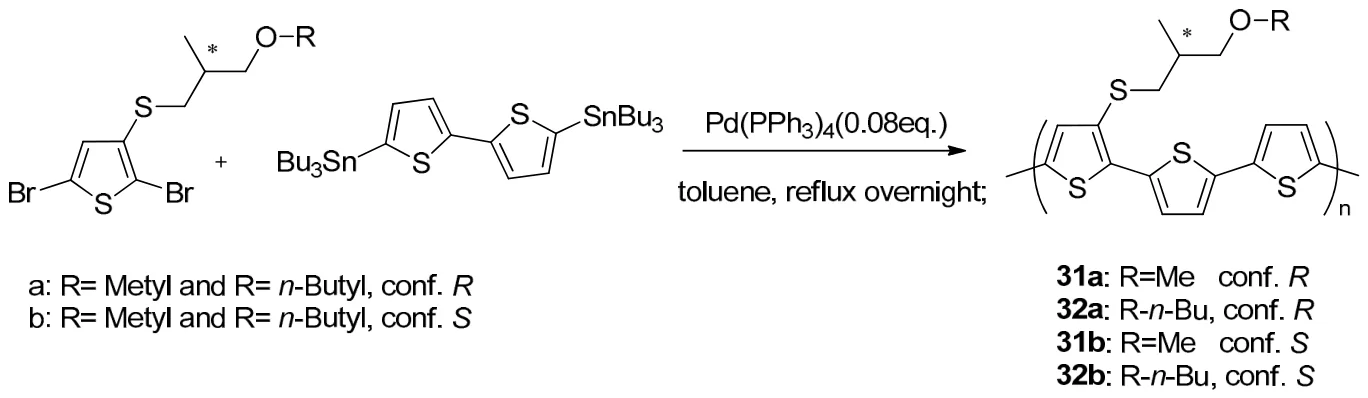
Synthesis of poly(terthiophenes) containing alkoxyalkylsulfanyl side chains with one *R* or *S* stereogenic carbon atom.

A similar Stille coupling polycondensation was applied to produce polythiophenes **31**, **32** featuring α, α′-conjugated thiophene backbone of alternating bithiophene and 3-(alkoxyalkylthio)thiophene units (Scheme 8) [48]. The polythiophenes, containing alkyl side chains with one (*R*)- or (*S*)-chiral carbon atom were converted into enantiopure chiral nanoparticles ((*R*)-or (*S*)-NPs) via reprecipitation. 

The (*S*)-polymer exhibited a blue-shifted absorption relative to the (*R*)-enantiomer, indicating lower solution-phase aggregation. In the nano-assembled state, (*R*)-NPs were blue-shifted compared to (*S*)-NPs. Moreover, comparing the spectra of the NPs with the solution spectra of polymers, the growth of an absorption peak at 580 nm was observed, which was attributed to dense interchain aggregation within the nanoparticle cores. The quantum yields of *R* and *S* polymers **32a** and **32b** in THF and of the nanoparticles in water suspension were measured, indicating values of 10% for the polymers and under 1% for the NPs, nevertheless, the NP luminescence remained sufficient for intracellular imaging. Circular dichroism confirmed the transfer of chirality from the side chains to the backbone within the π → π* transition region, with the NPs displaying a significantly enhanced Cotton effect relative to the molecularly dissolved polymers.

**Scheme 9:**
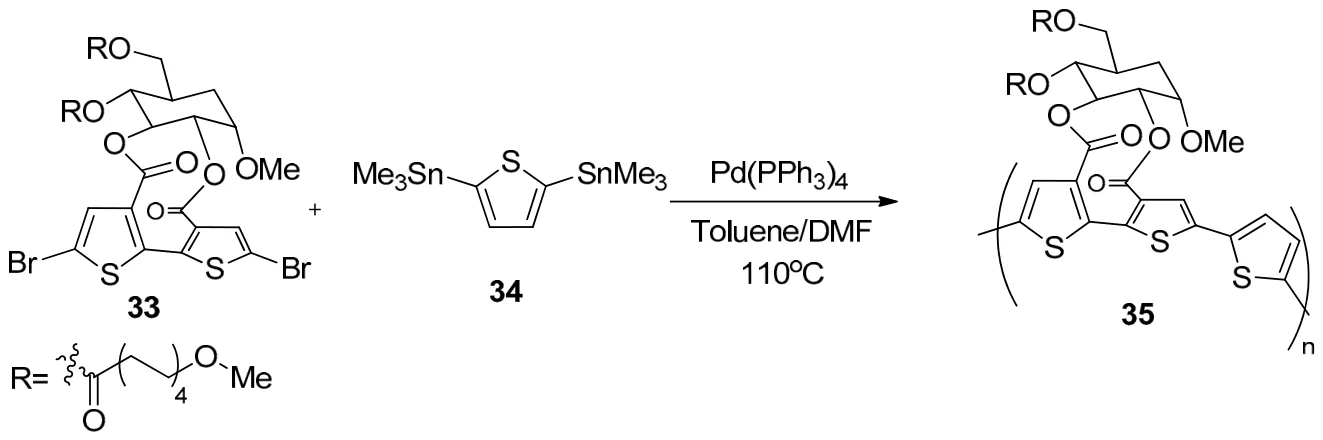
Synthesis of one-handed helical, glucose-decorated poly(terthiophene).

The procedure *via* Stille coupling (co)polymerization was applied to link a glucose-decorated 5,5′-dibromobithiophene **33** with 2,5-bis(stannyl)thiophene **34** to provide an optically active, one-handed helical polythiophene built up from an axially chiral bithiophene **35** (Scheme 9) [19]. Owing to the structurally constrained *syn*-conformation of the bithiophene unit, the polymer adopted helical conformation which persisted in solution without requiring aggregate formation. The intense CD signals arisen from the helical chirality of **35** were even present in such good solvents, as tetrahydrofuran and chlorobenzene [see Section 2.2.1]. This system represents the first example of a one-handed helical polythiophene in good solvents whose backbone conformation remains essentially invariant to environmental factors such as changes in concentration, temperature, or solvent polarity.

f. GRIM Grignard Metathesis Polymerization/Kumada Catalyst-Transfer Polycondensation KCTP

Grignard metathesis (GRIM) is a nickel-catalyzed cross-coupling polymerization utilizing *i*-PrMgCl, and [Ni(dppp)Cl_2_]. The mechanism involves a catalytic cycle consisting of three key steps: oxidative addition, transmetalation, and reductive elimination. This method was first developed in 1999 by McCullough [11,49] and was subsequently advanced by Yokozawa et al. [50,51]. Later, this regiocontrolled polymerization pathway was established as Kumada catalyst-transfer polycondensation (KCTP). Further studies provided deeper insight into the mechanism, revealing that KCTP proceeds via a “quasi-living” chain-growth mechanism that consistently yields polymers with exceptionally high HT regioregularity. This mechanism is characterized by unidirectional polymer chain propagation, where the addition of each monomeric unit regenerates the active catalytic species at the growing chain end.

**Scheme 10:**
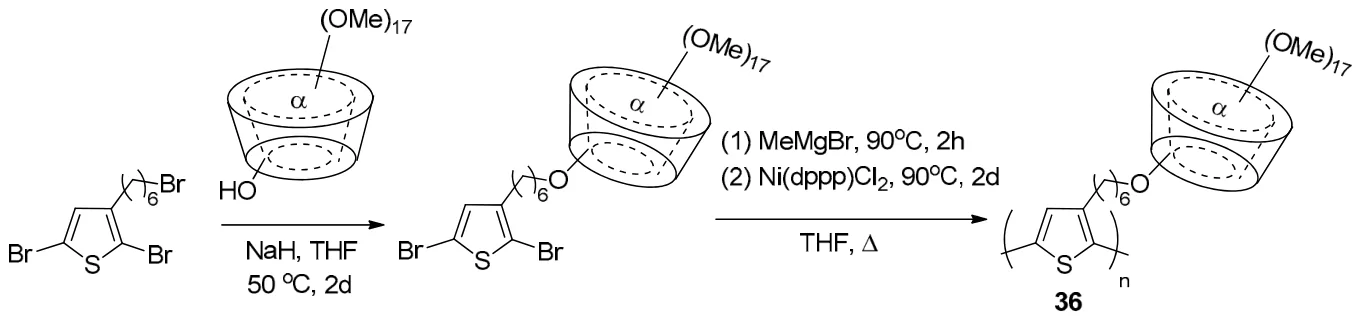
The preparation and polymerization of the permethyl-α-cyclodextrin-modified thiophene monomer.

Following this procedure [49], cyclodextrin-functionalized polythiophene **36** was prepared (Scheme 10) to evaluate its chiroptical sensing capabilities toward essential biomolecules [17].

The ^1^H and ^13^C NMR spectra of polymer **36** show one singlet proton signal in the aromatic region and four carbon resonance signals of the thiophene ring, indicating the formation of a uniformly head-to-tail coupled polythiophene. 

Evaluated in 50% aqueous methanol, **36** exhibited host–guest binding and chiral recognition toward various amino acid derivatives and dipeptides, among which Tyr and 4-NO_2_-Phe exhibited the highest binding affinities and superior enantiodiscrimination (also see Section 4.2).

To study how side-chain heteroatoms (oxygen vs. sulfur) affect macromolecular self-assembly, regioregular head-to-tail polythiophenes **38a**,**b** were synthesized from monothiophenes functionalized at the 3-position with either a chiral (*S*)-2-methylbutyloxy or (*S*)-2-methylbutylsulfanyl moiety **37a**,**b** according to GRIM polymerization [52].

**Scheme 11:**
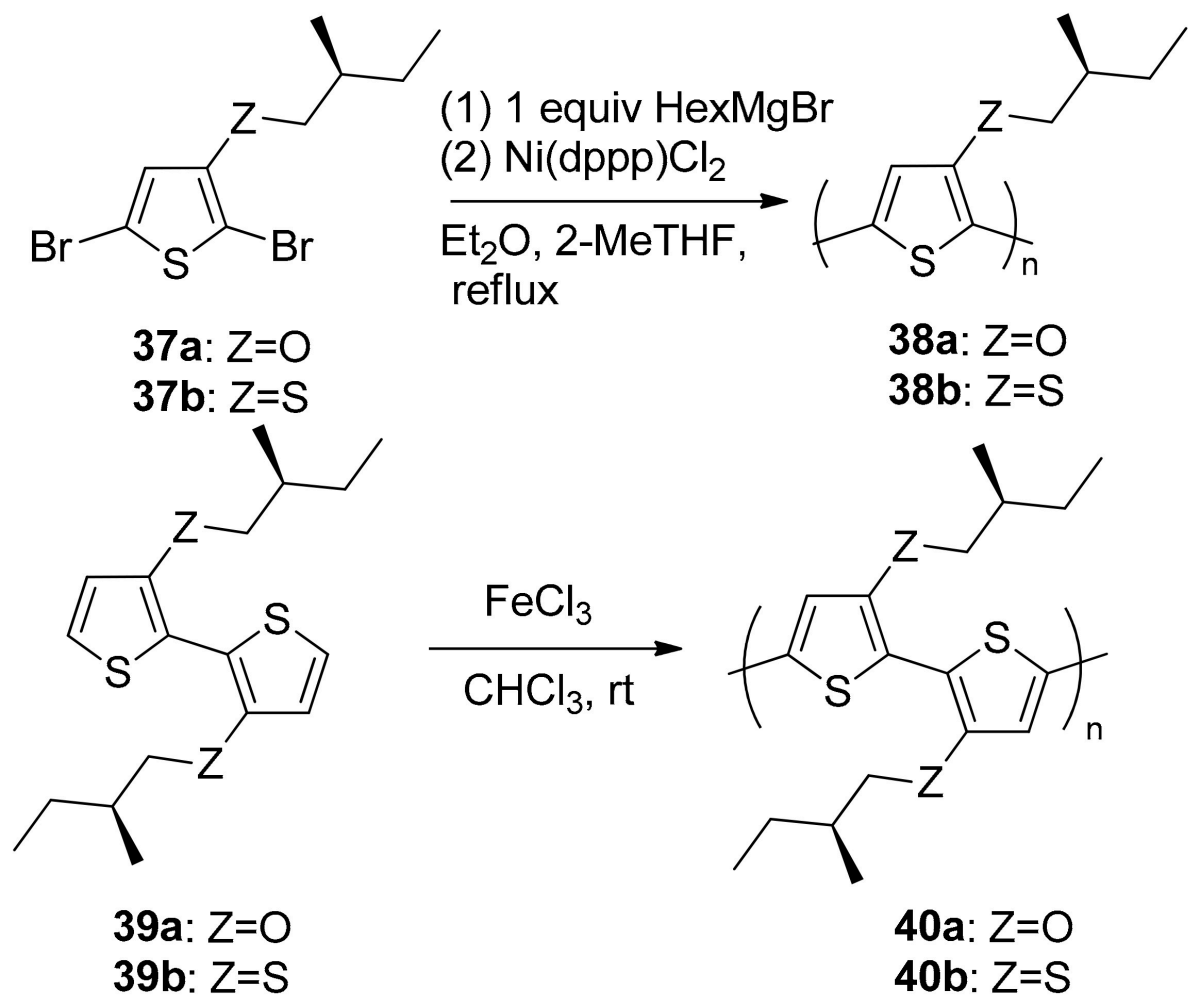
Synthesis of regioregular head-to-tail (HT) polythiophene **38a**,**b** and head-to-head/tail-to-tail (HH/TT) polymers **40a**,**b**.

Additionally, FeCl_3_-mediated oxidative coupling of bithiophenes **39a**,**b** yielded the corresponding head-to-head/tail-to-tail (HH/TT)-configured polymers **40a**,**b** (Scheme 11).

^1^H-NMR spectroscopy in CDCl_3_. revealed that alkylsulfanyl-functionalized polythiophene **38b** possesses 90% head-to-tail (HT) regioregularity (singlet at 7.38 ppm), whereas the alkoxy analogue 42a showed a broad aromatic signal indicating a predominantly HT configuration. For polymer **40b**, the ^1^H-NMR spectrum displayed a thienyl backbone proton (H-4) resonance at 7.2 ppm, alongside intense end-group signals, indicating a relatively low molecular weight for this sample. In contrast, the ^1^H-NMR spectrum of **40a** pointed to a regiorandom architecture, evidenced by three broad signals at 7.13, 6.97 (dominant), and 6.83 ppm. These split resonances were assigned to a complex mixture of regioirregular dyad and triad linkages such as HH/TT, HH/TT/HT, and HT/HT within a predominantly HH/TT-linked oligomeric framework. 

**Scheme 12:**
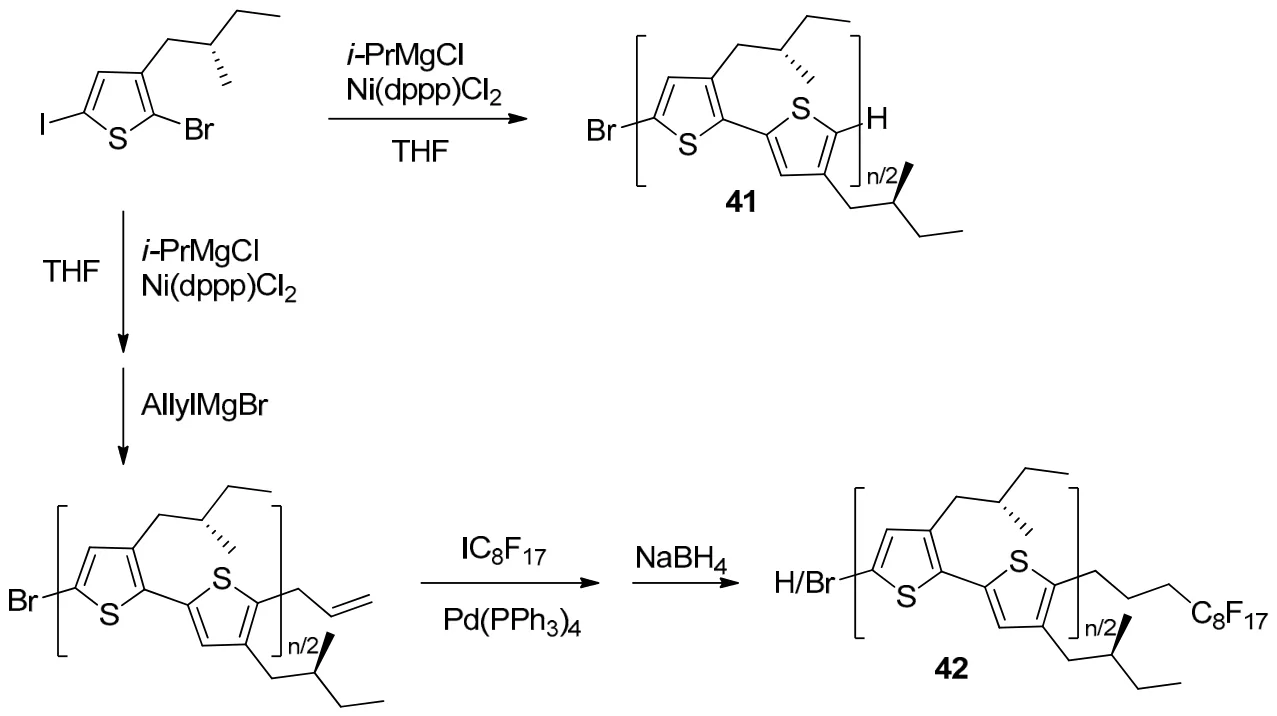
Synthesis of ((*S*)-P3MBT) **41** and ((*S*)-P3MBT-F17) **42**.

Regioregular head-to-tail (HT) poly(3-(*S*)-2-methylbutylthiophene) ((*S*)-P3MBT) **41** and its semifluoroalkyl end-functionalized analogue ((*S*)-P3MBT-F17) **42** were synthesized via Kumada catalyst-transfer polycondensation (KCTP) utilizing a nickel catalyst. (Scheme 12) [53,54].

**Scheme 13:**
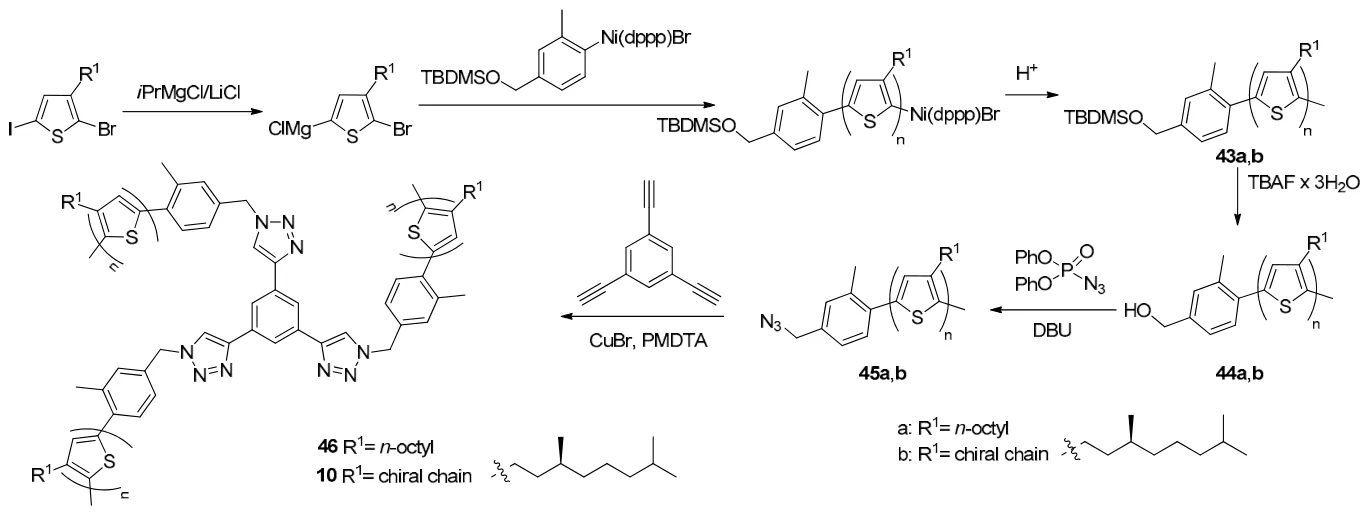
Synthesis of star-shaped, *C*_3_-symmetric compounds **10** and **46**.

Kumada catalyst-transfer polycondensation (KCTP) was selected to synthesize poly(3-alkylthiophene) arms, which were subsequently used to construct high-molecular-weight, star-shaped compounds **10** and **46** (Scheme 13) [24]. THF solution provided the polythiophenes **43a**,**b** with the required low dispersity and controlled molar mass. Three postpolymerization steps were next performed: (1) deprotection of the silyl-protected alcohol function of **43a** and **43b** with an excess of TBAF·3H_2_O to afford the compounds **44a** and **44b** (confirmed by ^1^H NMR upfield shift of the CH_2_ signal from 4.75 to 4.70 ppm); (2) azide formation by reacting **44a** and **44b** with diphenylphosphoryl azide and 1,8-diazabicycloundec-7-ene (DBU); and (3) click-coupling, a copper(I)-catalyzed alkyne-azide cycloaddition, by attaching the azide-functionalized chiral and achiral P3AT arms of **45a**,**b** to the central core in the presence PMDTA and CuBr, to yield the star-shaped polymers **10** and **46**.

Solvatochromic UV-Vis and CD measurements in chloroform/methanol mixtures showed that methanol addition induces self-assembly in star-shaped (**10**, **46**) and linear (**45a**,**b**) polymers, which adopt random-coil conformations in pure chloroform.

For achiral polymers, this resulted in a red-shifted UV-Vis absorption band with fine structure but no CD signal. For chiral polymers, it triggered a strong bisignate Cotton effect, confirming the formation of a chiral supramolecular structure. In comparison, the linear polymers (**45a**,**b**) exhibited weaker aggregation. Notably, the *C*_3_-symmetric chiral star polymer displayed a 100-fold stronger CD response than its linear arm counterpart, underscoring a greatly enhanced chiral organization driven by the star-geometry.

**Scheme 14:**
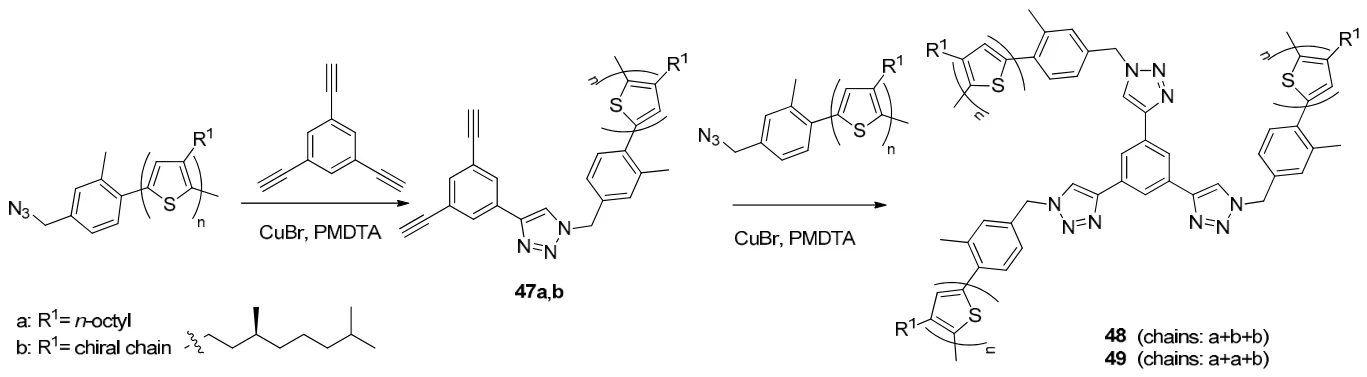
Synthetic pathway to the heterogeneous star-shaped P3ATs (**48** and **49**).

The same synthetic pathway was applied to prepare mixed-arm star analogues **48**, **49** containing both chiral and achiral polythiophene arms (Scheme 14) [25]. The main objective of this study was to investigate how incorporating different types of arms into the star-shaped system impacts its supramolecular organization and chiral response. Using UV-Vis, CD, DSC, and AFM analyses, three models were evaluated (“sergeants-and-soldiers”, “defect effect”, and “hampered organization”). Only the **hampered organization** model was applicable, as replacing even a single chiral arm with an achiral substitute introduced structural defects that severely disrupted self-assembly.

Supramolecular experiments in chloroform/methanol mixtures revealed aggregation onsets at 40% MeOH for **48** and 35% MeOH for **49**. Compared to the ***C***_3_-symmetric chiral star polymer **47b**, both **48** and **49** displayed weaker UV-Vis fine structures and lower bisignate Cotton signals in their CD spectra, confirming less ordered aggregation under the hampered organization hypothesis. Temperature-variable CD spectroscopy proved unsuitable for evaluating chiral expression, as it produced weaker signals lacking a positive bisignate Cotton effect.

Finally, UV-Vis and CD spectra were recorded for spin-coated films of **48** and **49** both before and after thermal annealing. Unannealed samples mirrored solution solvatochromism, confirming hampered organization in the solid state. Upon thermal annealing, the chiral response decreased for **48** but increased for **49**, with both losing their bisignate Cotton shape. Complementary AFM imaging pointed to a tendency to organize into well-defined fibers. 

**Scheme 15:**
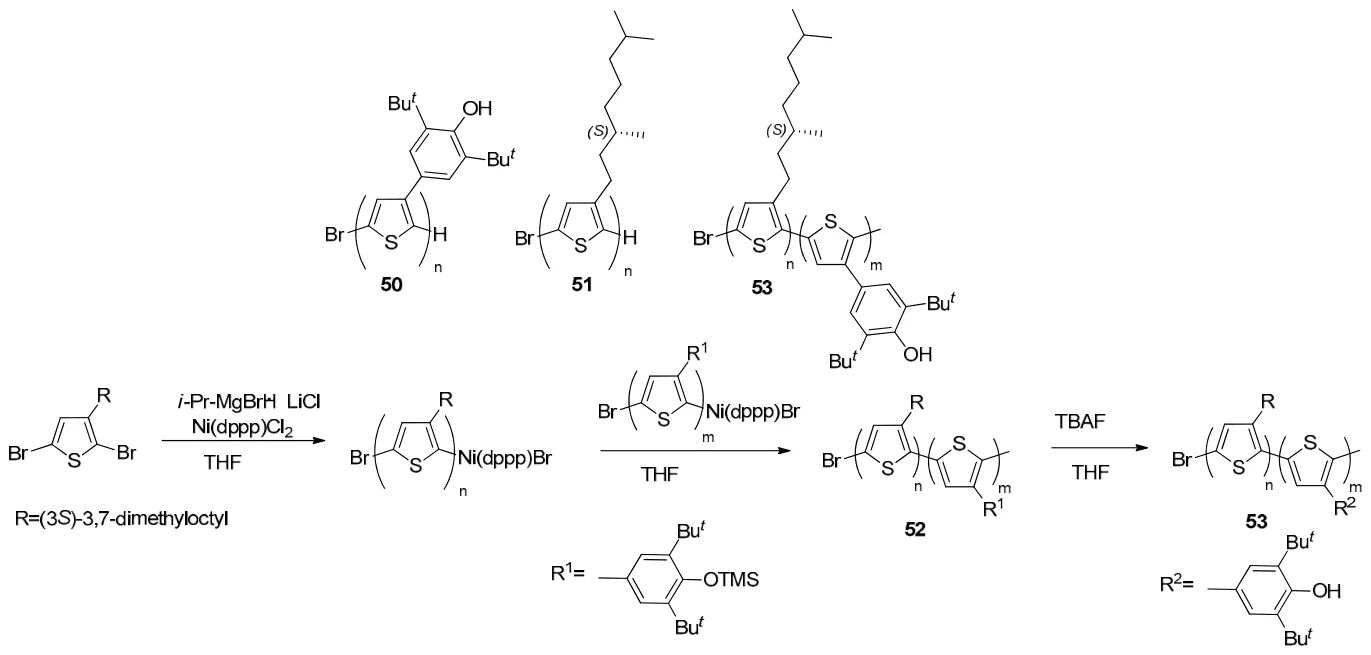
Synthesis of homopolymers **50**, **51** and copolymers **52**, **53**.

Highly stereoregular, chiral magnetic block copolymers **52**, **53** containing a polythiophene backbone, a magnetic phenoxy radical and chiral side chains were synthesized via a Grignard metathesis (GRIM) (Scheme 15). The two corresponding homopolymers **50**, **51** were also prepared. Chiral substituents successfully induced one-handed chiral J-aggregation within block copolymer [55].

Electron spin resonance (ESR) spectroscopy confirmed the generation of phenoxy radicals, suggesting potential antiferromagnetic behavior. Ultimately, the investigation of its optical activity and spin electronics demonstrates that the polymer successfully incorporates both optically active and magnetic blocks.

g. Dehydrobrominative Ni-Catalyzed Polycondensation with Knochel-Hauser Base

**Scheme 16:**
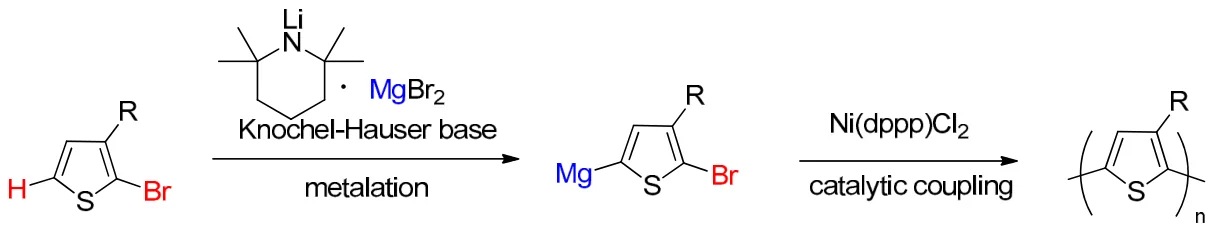
General concept.

Another valuable synthetic method for nickel-catalyzed polymerization is a Kumada coupling variant [56] (Scheme 16) based on dehydrobrominative polycondensation using a Knochel-Hauser base. 

**Scheme 17:**
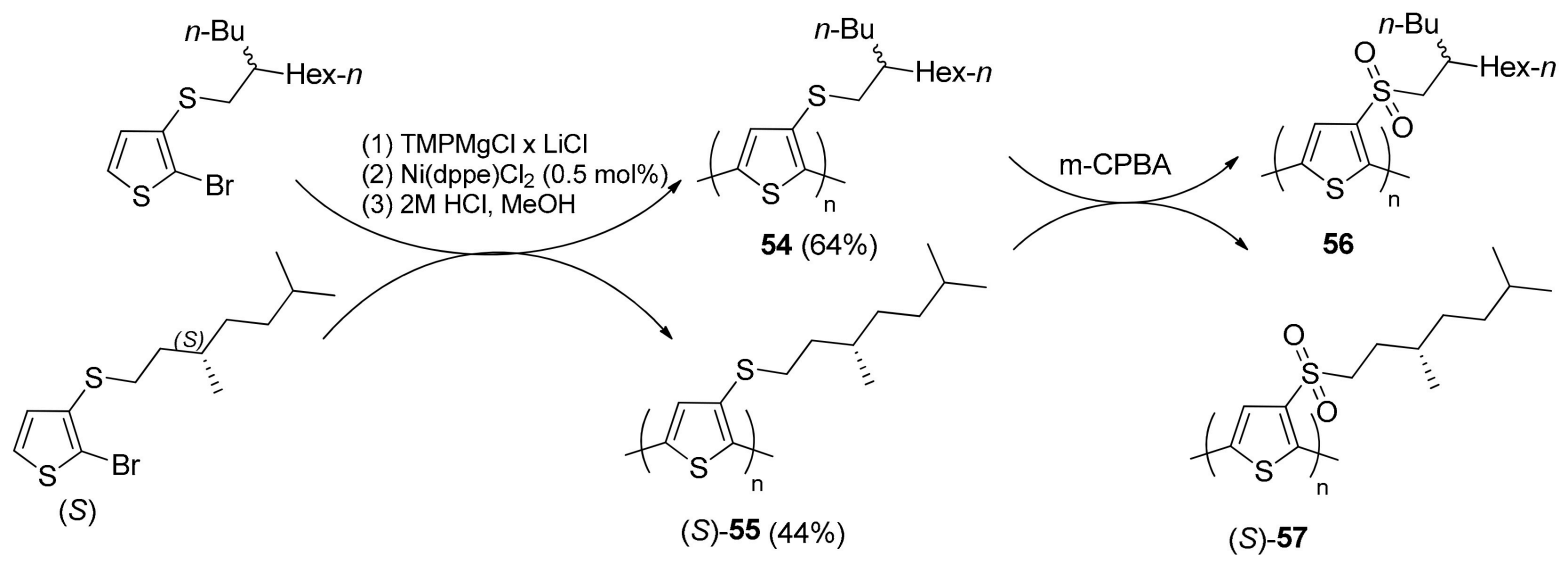
Synthesis of chiral regioregular poly(3-(alkylsulfanyl)thiophene)s **54**, **55** and poly(3-(alkylsulfonyl)thiophene)s, **56** and **57**.

This synthetic route was exploited to prepare regioregular poly(3-(alkylsulfanyl)thiophene)s which upon a post-polymerization oxidation were converted to the corresponding poly(3-(alkylsulfonyl)thiophene)s (Scheme 17) [57].

The resulting chiral helical poly(3-(alkylsulfonyl)thiophene)s, **56** and **57** exhibited high magneto-optic (MO) activity, distinguished by an enormous Faraday effect with Verdet constants reaching up to (7.63 ± 0.78) × 104 deg T^−1^ m^−1^ at 532 nm. The sign and magnitude of the Verdet constant depended directly on polymer helicity, establishing a promising strategy for designing novel materials with tailored magneto-optic properties.

In solution, (*S*)-**55** and (*S*)-SO_2_-**67** displayed strong bisignate Cotton effects for the π → π* transitions. No Cotton effect was observed for racemic **54** and **56**, nor for (*S*)-**57** in pure THF. However, adding methanol as a poor solvent induced a strong CD signal for (*S*)-**57**, confirming ordered helical aggregation. The spectrum revealed first a negative-then-positive CD band at longer wavelengths, implying P-helicity with a right-handed chiral assembly.

h. RuPhos Protocol–(Pd-catalyzed Negishi Coupling Polymerization)

**Scheme 18:**
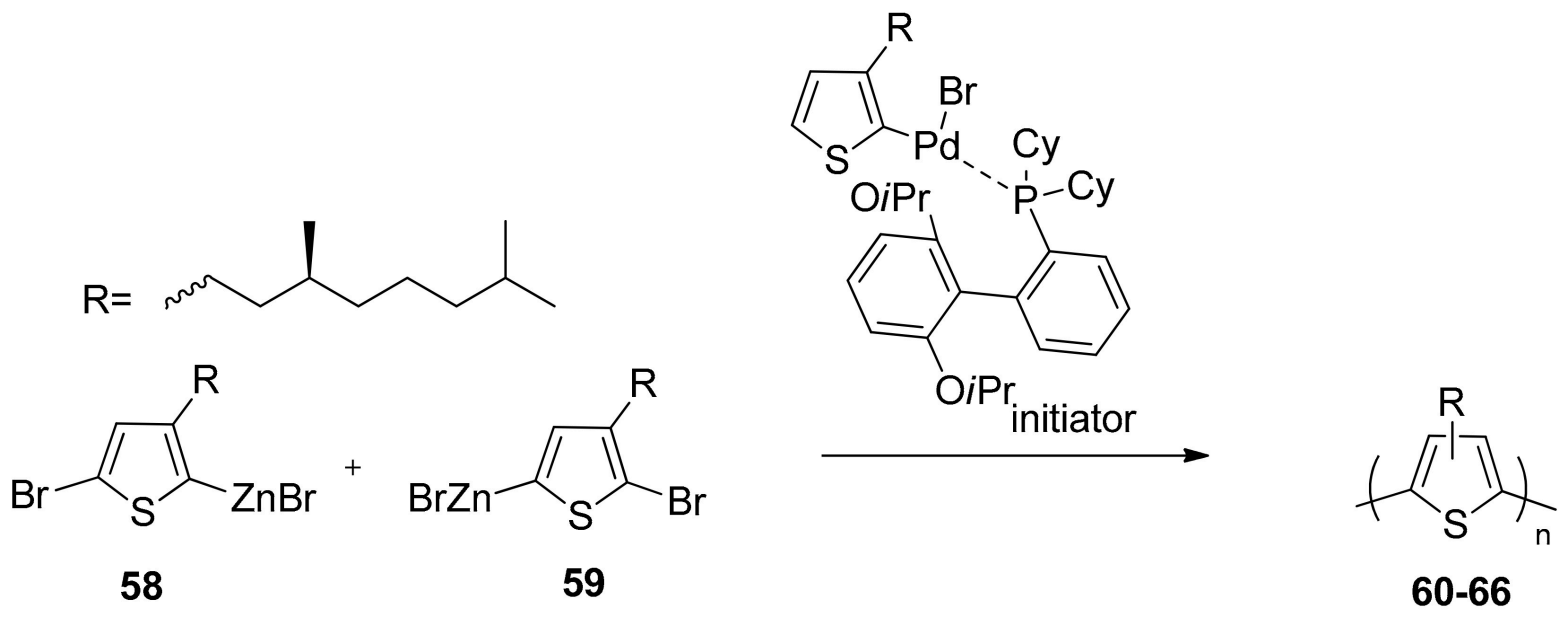
Synthesis of a series of chiral poly(3-alkylthiophenes) with a different regiochemistry (**64**–**70**).

Another strategy to synthesize regioregular polythiophenes is Pd–RuPhos-catalyzed Negishi polymerization using organozinc halide intermediates generated from a Pd–RuPhos–thiophene initiator. This chain-growth polymerization yielded a series of chiral poly(3-alkylthiophene)s (**60**–**66**), with regioregularity controlled by adjusting the ratio of organozinc isomers **58** (2-bromo-5-bromozincio-3-alkylthiophene) and **59** (2-bromozincio-5-bromo-3-alkylthiophene) (Scheme 18) [58].

This synthetic strategy incorporated all three coupling configurations, head-to-tail (HT), tail-to-tail (TT), and head-to-head (HH), into the polythiophene backbone, enabling a systematic study of regioregularity–property relationships via UV-Vis, CD, DSC, and AFM. It was hypothesized that the percentage of HT couplings can be predicted, provided that all coupling types are integrated via a statistical copolymerization of the two constituent isomers. Theoretically, increasing the ratio of isomer **58** should yield a higher proportion of HH couplings, reaching a maximum when equimolar amounts of both isomers were utilized. The experimentally obtained dyad compositions of polymers **60**–**66** were quantified using ^1^H NMR spectroscopy and compared against theoretical statistical curves. The experimental data closely matched the theoretical values (with a minor deviation for **66**), confirming a true statistical copolymerization mechanism. Finally, the average molar masses and dispersities (PDI) of the resulting polymers were evaluated via gel permeation chromatography and are summarized in Table 1.

**Scheme 19:**
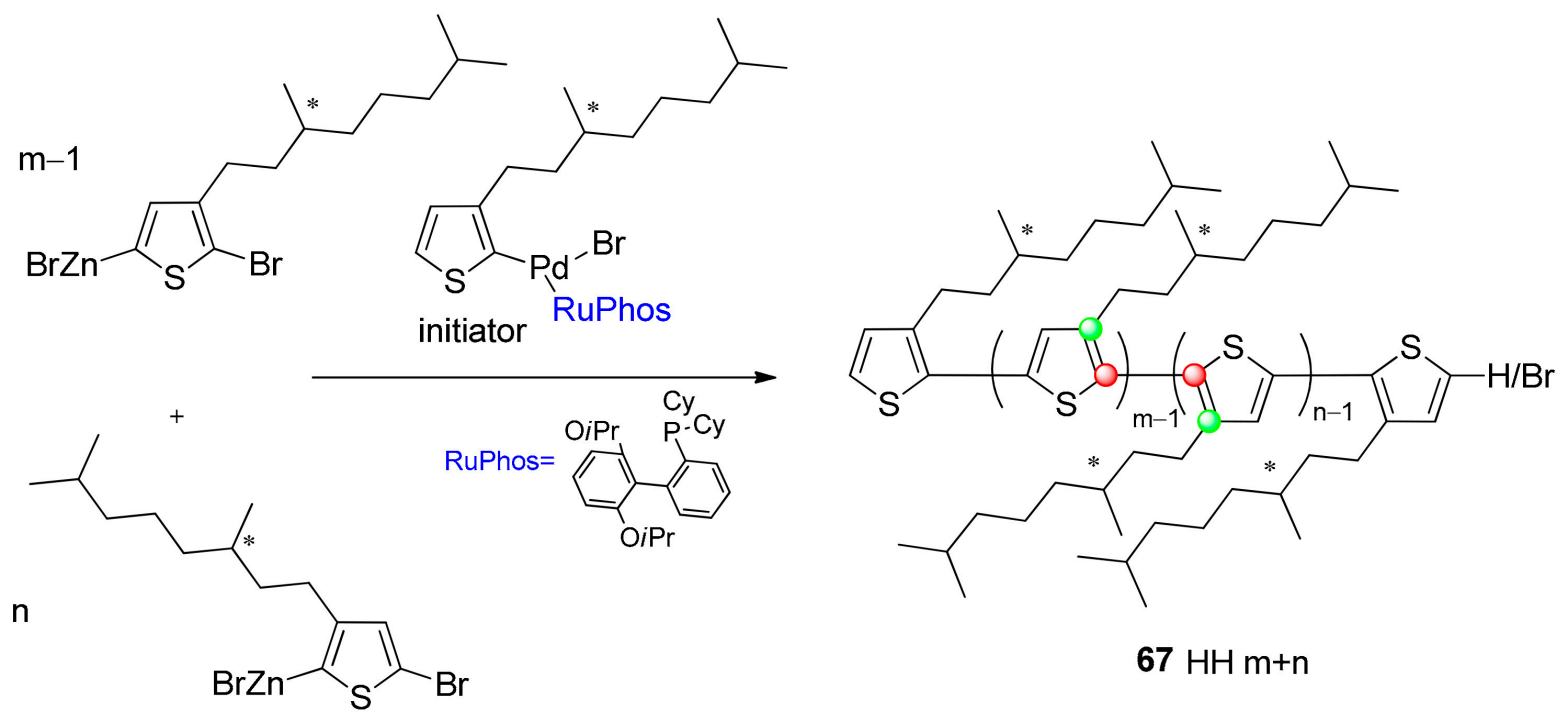
Synthesis of poly(3-alkylthiophene) **67** containing a single head-to-head (HH) type coupling.

**Scheme 20:**
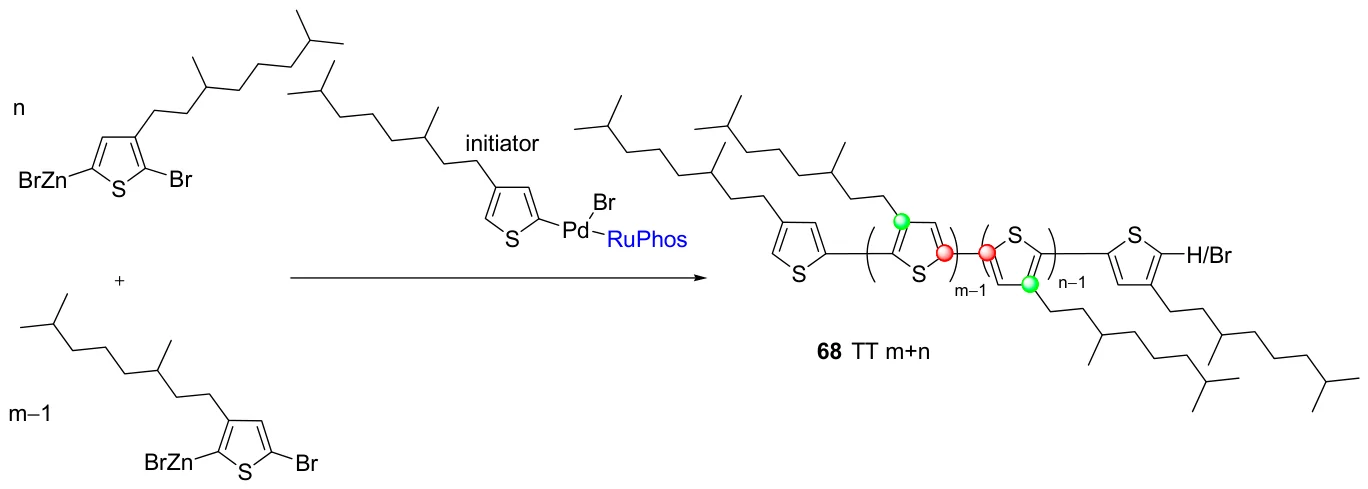
Synthesis of poly(3-alkylthiophene) **68** containing a single tail-to-tail (TT) type coupling.

**Scheme 21:**
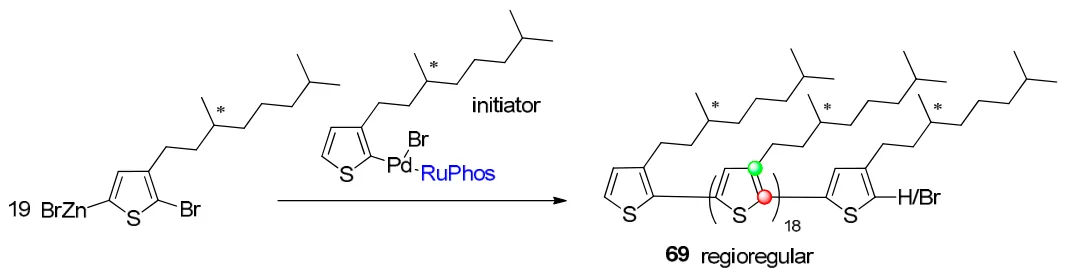
Synthesis of regioregular poly(3-alkylthiophene) **69**.

Chiral poly(3-alkylthiophene)s (P3ATs) containing a single head-to-head (HH) **67** or tail-to-tail (TT) regiodefect **68** at varied backbone positions were synthesized via the controlled Pd–RuPhos protocol (Scheme 19 and Scheme 20) [59]. ^1^H NMR spectroscopy confirmed the degree of polymerization and defect incorporation through triad signal assignments following the method of Barbarella et al. [60]. This analysis enabled the establishment of the chemical shifts of aromatic protons dependent on their specific triad environment (HT-HT, HT-TT, HT-HH, or TT-HH). Supramolecular assembly was monitored via UV-Vis and CD solvatochromism experiments in chloroform/methanol. For HH-defective polymers, a single terminal defect enhanced conformational flexibility, preventing kinetic trapping, and yielded a higher CD response than the fully regioregular polymer **69**. Shifting the HH defect toward the chain center shortened regioregular segments, lowering chiral packing efficiency (*g*_abs_) and CD signal. In contrast, TT defects generally reduced the chiral response relative to regioregular **69** (see synthetic approach, Scheme 21), with their optimal position shifting closer to the chain center compared to HH analogues.

#### 3.1.2. Synthetic Methods for Chiral Polythiophenes with Regiorandom Type of Couplings

**Scheme 22:**
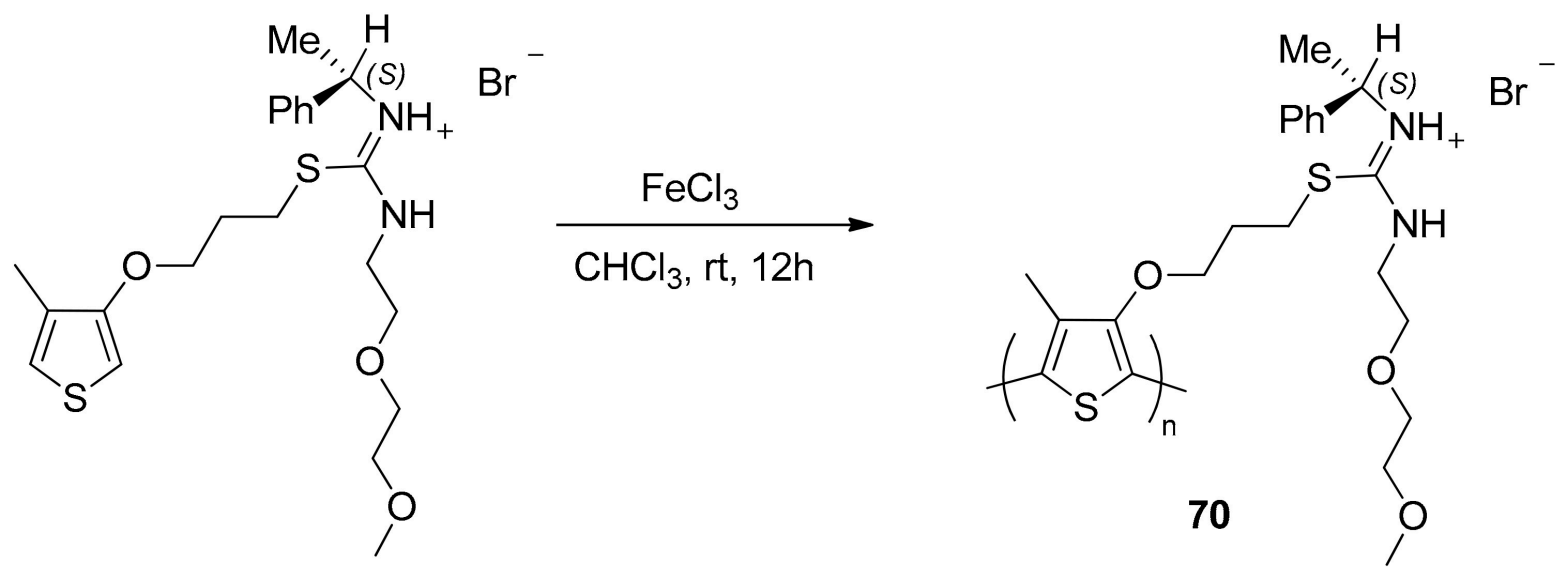
Synthesis of optically active polythiophene via oxidative coupling according to Sugimoto procedure.

FeCl_3_-Mediated oxidative coupling was employed to synthesize an optically active polythiophene **70** functionalized with chiral isothiouronium pendant groups (Scheme 22). Specific anion addition induced helical chirality through supramolecular aggregation [61].

In a 1:1 (*v*/*v*) H_2_O–MeOH mixture, polythiophene **70** exhibited an absorption band at 420 nm and no CD signal, indicating a random-coil conformation. The addition of the ${PF}_{6}^{-}$ anion altered both the UV-Vis and CD spectra; specifically, by inducing a bathochromic shift alongside a split-induced CD (ICD) pattern within the π → π* transition region. FE-SEM confirmed ${\;PF}_{6}^{-}$ anion—driven helical aggregation, revealing a networked fibrillar morphology.

**Figure 12:**
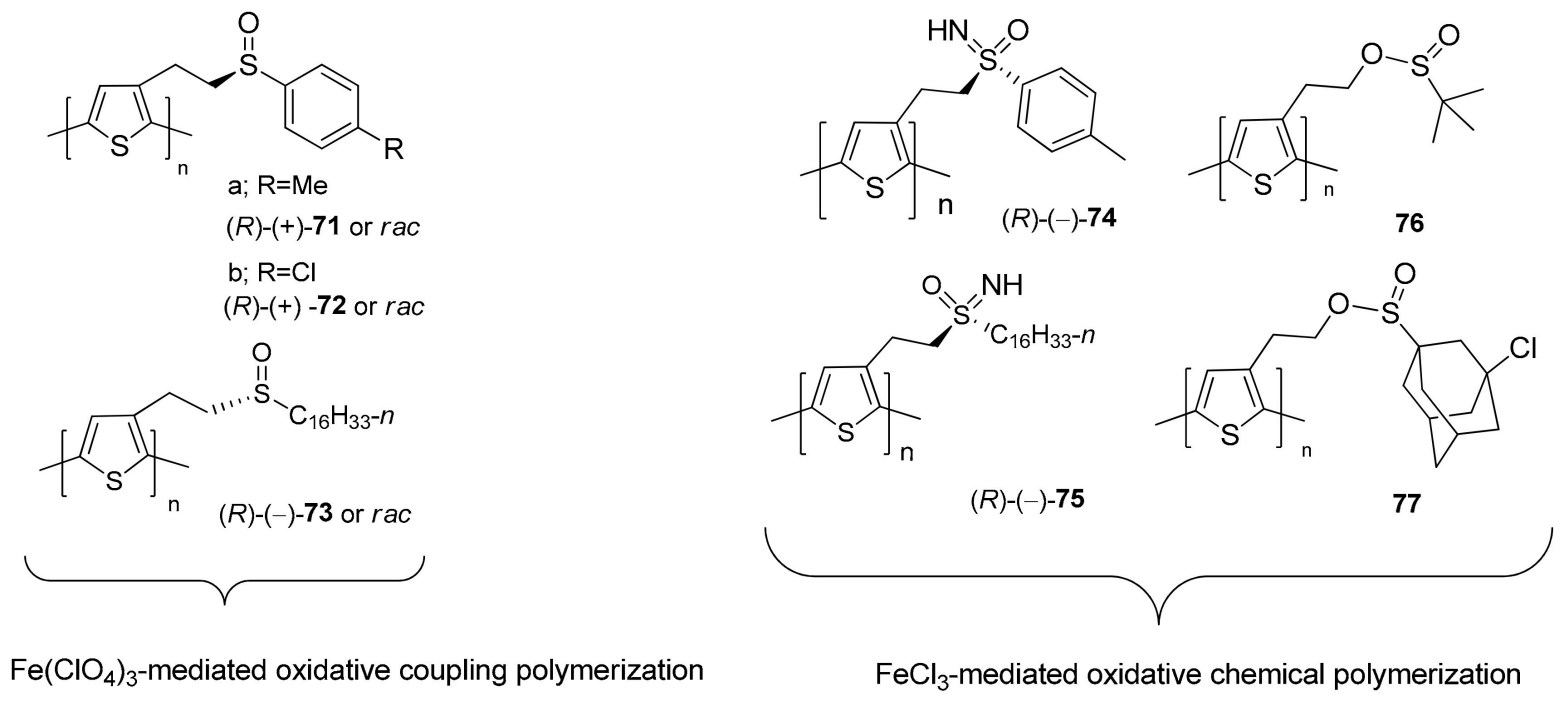
A series of regiorandom chiral polythiophenes **71**–**77** containing side chains with a point stereogenity at a sulfur atom.

Recently, chemical oxidative polymerization has been utilized to prepare novel chiral polythiophenes **71**–**77** [62]. The resulting polythiophenes contained at the 3-position of the thiophene rings a pendant group featuring a stereogenic sulfur atom (in the structures of sulfoxides **71**–**73**, sulfoximines **74**, **75**, or sulfinates **76**, **77**) (Figure 12). FeCl_3_-mediated oxidative coupling of enantiomerically pure sulfoxide-based thiophenes caused racemization at the side-chain sulfur stereocenter due to generated HCl, whereas using Fe(ClO_4_)_3_ during oxidative coupling preserved optical activity; sulfoximines and sulfinates resisted racemization under all conditions. This resistance to racemization is attributed to the high configurational stability of the sulfoximine and sulfinate side chains at sulfur. Beyond the optical activity originating from the localized chiral centers, CD spectroscopy confirmed induced chirality within the conjugated polythiophene backbone. Finally, solution fluorescence quantum yields (Φ_F_), determined using the relative Williams method, ranged from 6% to 24%. 

### 3.2. Electrochemical Polymerizations

#### 3.2.1. Polythiophenes Functionalized with Chiral Substituents

**Figure 13:**
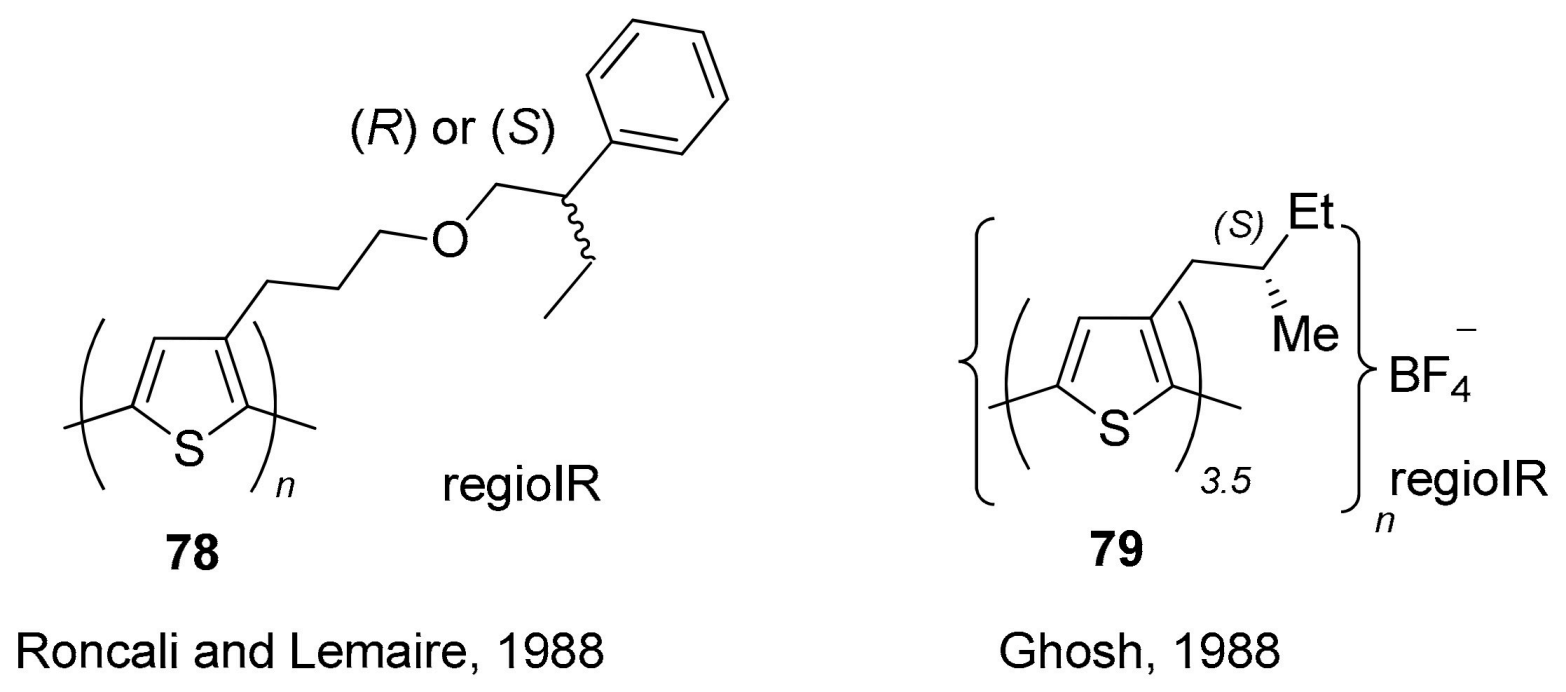
Polythiophenes **78** and **79** obtained by electropolymerization.

In 1988, Roncali and Lemaire reported the synthesis of chiral, 3-alkoxyalkyl-substituted polythiophenes **78** via electropolymerization (Figure 13) [63,64]. Although the resulting undoped polymer film possessed random regiochemistry, it still exhibited a remarkably high specific rotation of +3000 or −3000 for the respective opposite enantiomers. A comparison with the specific rotation of the corresponding monomers, which showed values of +22.5 or −22.5 (2%, CHCl_3_), clearly demonstrated the effect of macromolecular stereogenicity. Additionally, these polymers showed chiral discrimination toward two doping agents, (+)- and (−)-10-camphorsulfonic acid, owing to the formation of diastereomeric salts in the oxidized state. Through this example, the authors demonstrated that pioneering amperometric stereoselective recognition using doping agents.

**Scheme 23:**
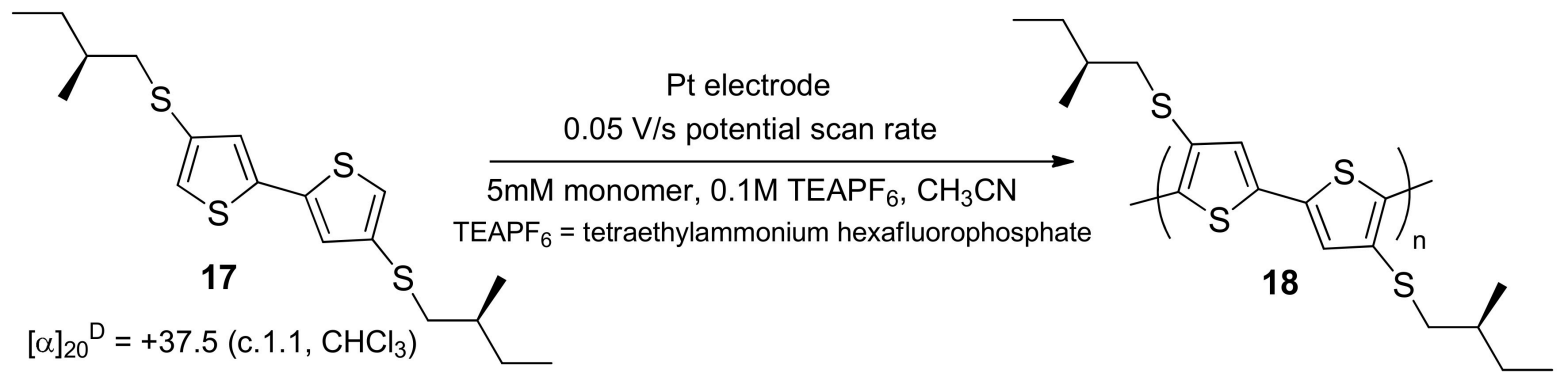
Synthesis of poly(β,β′-disubstituted bithiophene) **18**.

Ghosh and coworkers electropolymerized (*S*)-(+)-3-(2-methylbutyl)thiophene to yield chiral polythiophene **79** as a potential asymmetric electrode for chiral electrosynthesis (Figure 13) [65]. In 2001, the electrochemical polymerization of 2,2′-bithiophene functionalized with (*S*)-2-methylbutylsulfanyl chains **17** was employed in the synthesis of a regiosymmetric head-to-head/tail-to-tail poly(β,β′-disubstituted bithiophene) **18** (Scheme 23) [39]. The polymer exhibited different electrochemical behavior in tetraethyl- and tetrabutylammonium hexafluorophosphates as supporting electrolytes, indicating a dependence on the cation size.

**Scheme 24:**
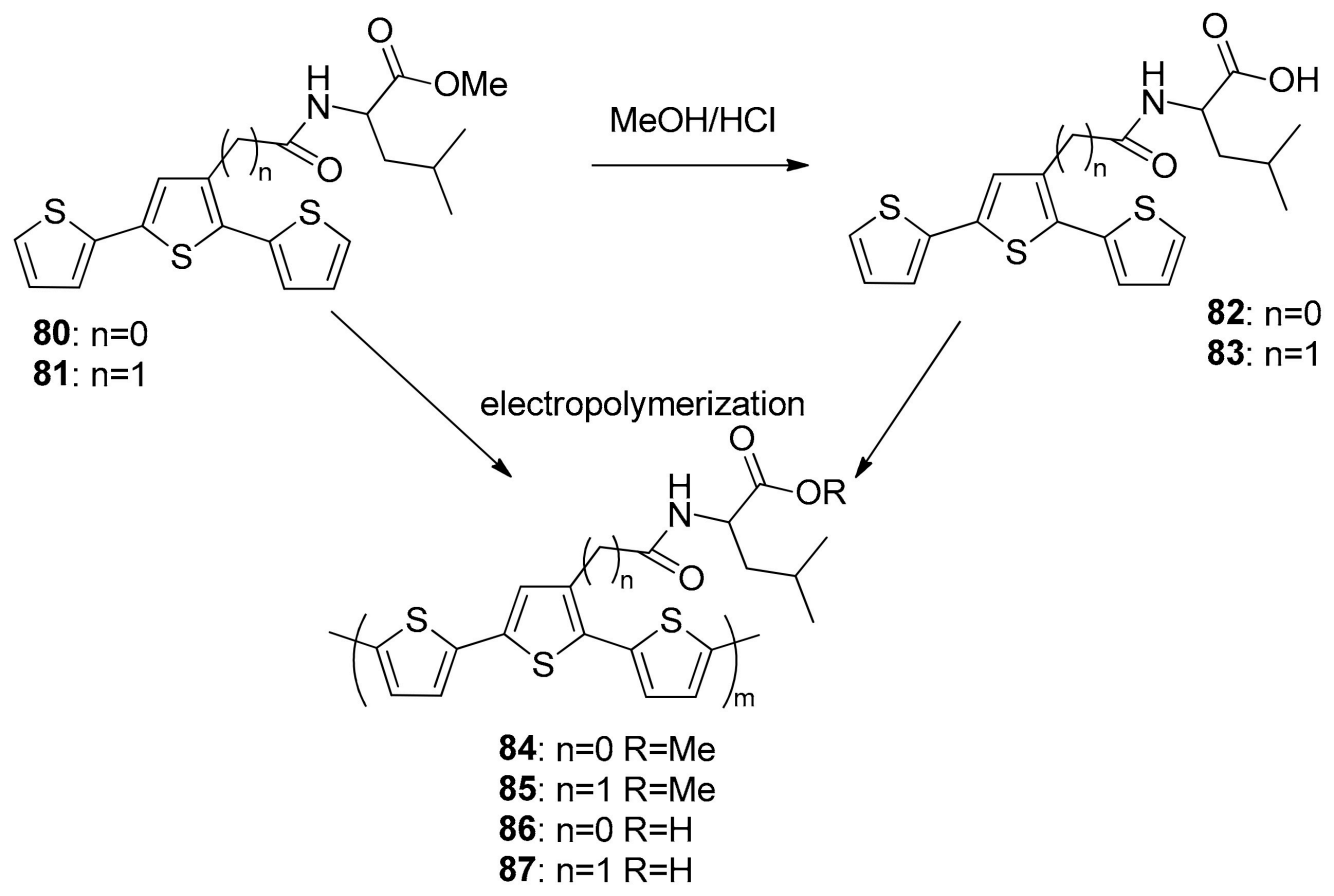
Electropolymerization of terthiophenes **80**–**83** in a 1 M *n*-Bu_4_NPF_6_/acetonitrile solution through repeated cyclic voltammetry (CV) cycling beyond the oxidation peak potential of the thiophene core-specifically at 0.660 V and 0.670 V (vs. Fc+/Fc) for compounds **84** and **87**.

The self-assembly of regiosymmetric polythiophene **18**, both in solution and in the solid state, was characterized via UV–Vis, CD, and AFM. Circular dichroism (CD) spectroscopy revealed a weak, bisignate Cotton effect with a positive signal at longer wavelengths, comparable to those reported for chemically synthesized samples. However, this weak signal corresponding to low optical activity likely arose from rapid film formation. As monitored by UV–Vis spectroscopy, macromolecular aggregation can be induced by the addition of a non-polar poor solvent (e.g., *n*-hexane) to the chloroform solution. Atomic force microscopy (AFM) morphological analysis revealed a uniform, dune-like topography.

l-leucine-functionalized terthiophenes **80**–**83** were electropolymerized to yield chiral, conducting polymer surfaces (Scheme 24) [66]. overcoming the enantioselective recognition limitations of earlier alanine-bearing polythiophenes [67]. Subsequently, the resulting poly(terthiophene)s **84**–**87** were examined to determine whether their chiral conducting surfaces were capable of hydrogen-bonding interactions with other amino acids. For the **87-Pt** electrode, cyclic voltammetry in the presence of d,l-alanine methyl ester revealed a concentration-dependent potential shift. Surface hydrogen-bonding interactions established a barrier that hinders electron transfer. Notably, **87-Pt** formed stronger hydrogen bonds with l-leucine methyl ester than with d,l-alanine methyl ester. 

**Scheme 25:**
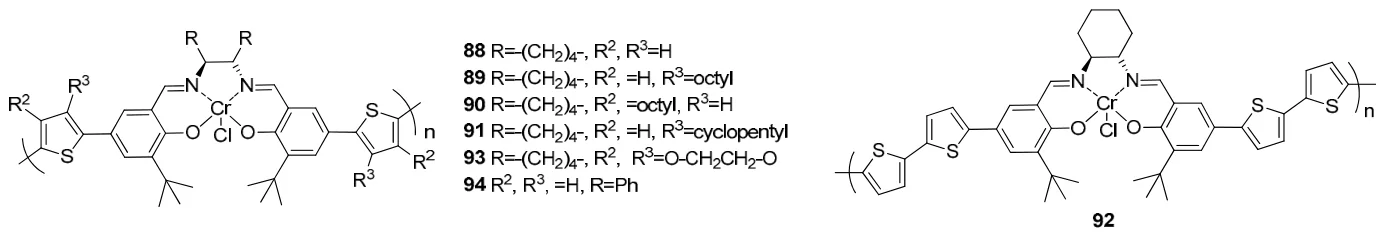
Polythiophenes derived from chiral salen–thiophene chromium complexes **88**–**94**.

Electrochemical oxidation of chiral salen–thiophene chromium complexes yielded polymers **88**–**94** on a preparative scale, isolated as insoluble black powders recovered from the electrode surface (Scheme 25) [68]. No electropolymerization occurred when using the metal-free salen–thiophene ligands.

**Scheme 26:**
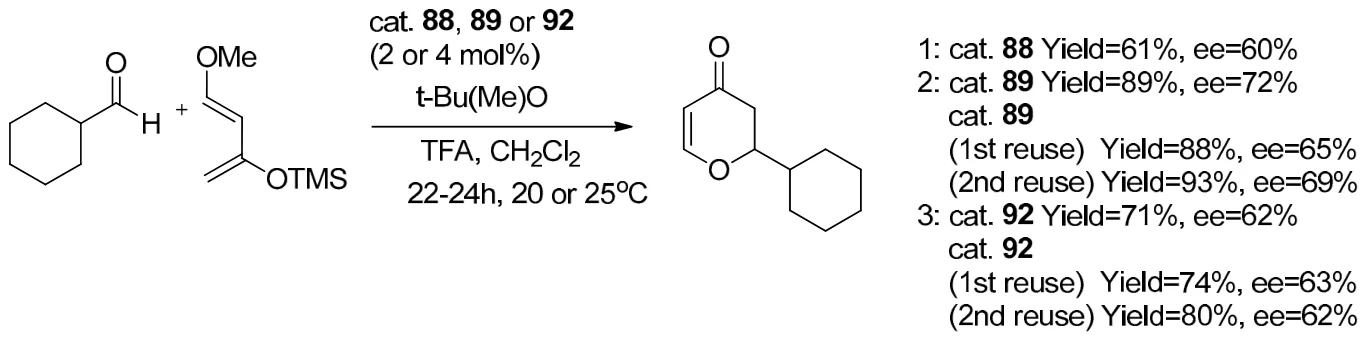
Evaluation of catalytic activity of **88**, **89**, **92** in hetero-Diels-Alder reaction.

**Scheme 27:**
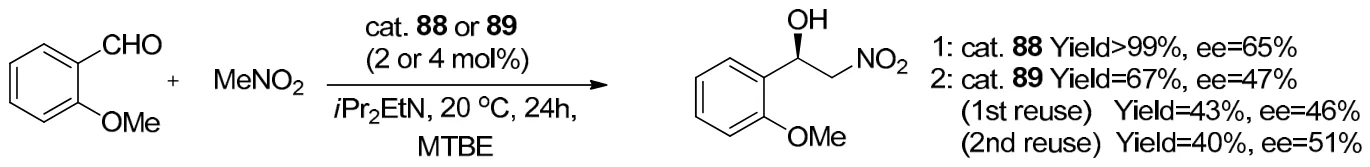
Enantioselective synthesis of 1-(2-methoxy-phenyl)-2-nitro-ethanol prompted by catalysts: **88** and **89**.

**Scheme 28:**
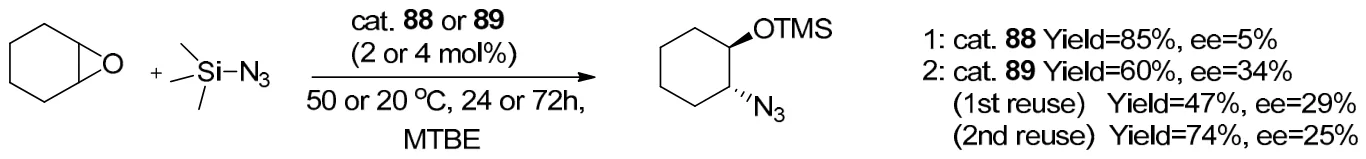
Ring opening of 7-oxa-bicyclo [4.1.0]heptane with trimethylsilylazide prompted by catalysts: **88** and **89**.

The resulting polymers were evaluated as heterogeneous catalysts for asymmetric hetero-Diels-Alder (HDA), Henry (nitroaldol) and epoxide ring-opening reactions. While high activities and enantioselectivities were generally achieved, the catalytic performance varied across the systems: Polymer **88** catalyzed the HDA reaction of various aldehydes, maintaining stable activity over 15 recycling cycles. In the Henry reaction of aldehydes with nitromethane, it was reused five times with minimal yield/*ee* loss. However, **88** exhibited poor performance in epoxide ring-opening compared to its homogeneous counterpart. Polymers **89** and **92** outperformed **88** in the synthesis of 2-cyclohexyl-2,3-dihydro-pyran-4-one, demonstrating higher catalytic activity. The reaction catalyzed by **89** yielded up to 72% *ee*. The catalytic performances of these polymeric systems are summarized in Scheme 26, Scheme 27 and Scheme 28 (see also Section 4.3).

**Figure 14:**
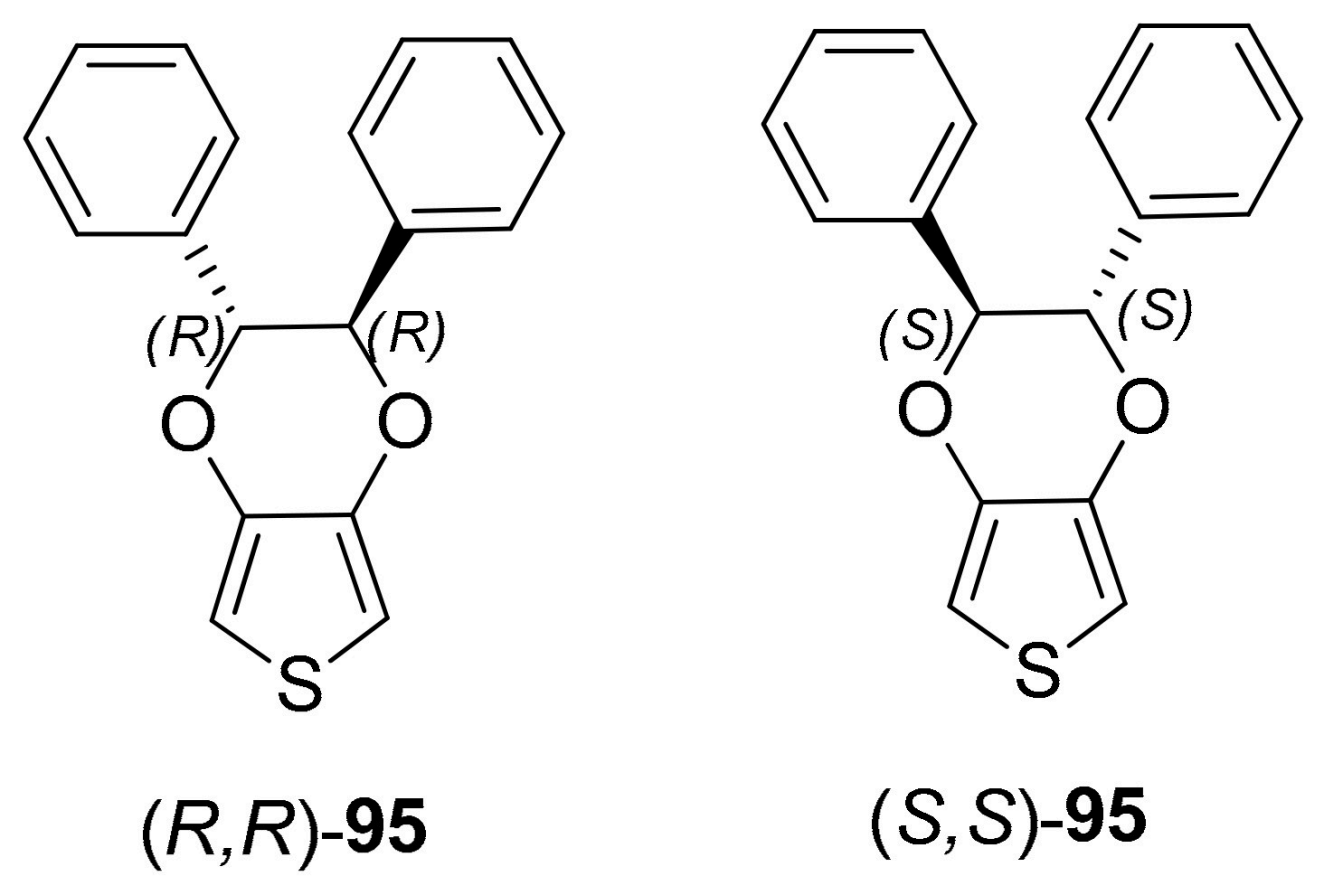
Structure of monomers (*R*,*R*)- and (*S*,*S*)-(**95**).

Electro-oxidative polymerization of enantiopure 3,4-ethylenedioxythiophene monomers, (*2R*,*3R*)- and (*2S*,*3S*)-2,3-diphenyl-3,4-ethylenedioxythiophene ((*R*,*R*)- and (*S*,*S*)-**95**, Figure 14), was found to be tunable via spin-polarized currents, depending on the electron spin orientation [69]. Electropolymerization was performed on a ferromagnetic electrode magnetized by an external magnetic field in either the “up” (pointing into the electrode surface) or “down” (pointing into the electrolyte) configuration. CD and UV–Vis spectra revealed that applying a magnetic field during electropolymerization significantly increased absorbance intensity, demonstrating enhanced chiral PEDOT film thickness relative to field-free conditions.

#### 3.2.2. Polythiophenes Lacking Chiral Substituents 

Asymmetric electropolymerization of achiral monomers in cholesteric (chiral nematic) liquid crystal (N*-LC) and liquid-crystalline ionic liquid (N*-LCILs) reaction media became a powerful method for synthesizing helical, π-conjugated polymers that lack chiral substituents. Helical PEDOT films synthesized in N*-LCs exhibited a distinct spiral morphology coupled with good electroactive properties [70,71]. Furthermore, asymmetric electropolymerization of EDOT in cholesteric liquid crystalline ionic liquids (N*-LCILs) was achieved without the need for either a supporting electrolyte or an external chiral dopant [72]. For these polymerization processes, the N*-LCILs were prepared using two distinct approaches: by adding an axially chiral binaphthyl derivative as an external chiral dopant to imidazolium-based LCILs; or by incorporating a chiral binaphthyl phosphate counteranion directly into the LCIL structure.

##### Electrochemical Polymerization in Asymmetric Reaction Templates: Chiral Transcription in Cholesteric Liquid Crystals

**Scheme 29:**
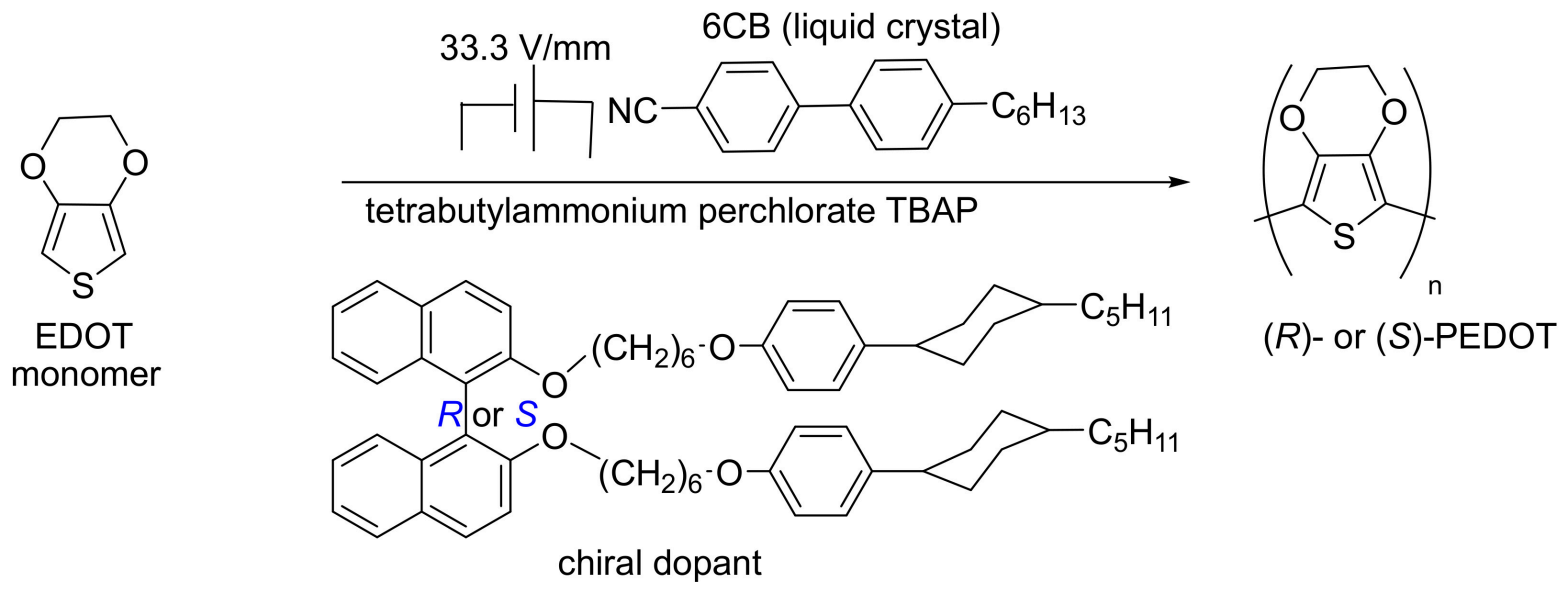
Electropolymerization of EDOT within the N*-LC environment.

Doping a nematic liquid crystal with a chiral molecule induces a chiral nematic phase (N*-LC, also referred to as the cholesteric phase) via the formation of a helical supramolecular structure. During the electrochemical oxidation of the monomer, the helical structure of the matrix forces the growing polymer chains into a helical π-stacked alignment, generating supramolecular chirality without requiring chiral monomer side chains. In line with this concept, asymmetric electropolymerization of 3,4-ethylenedioxythiophene (EDOT) was performed in a cholesteric liquid electrolyte mixture consisted of the liquid crystal 4-cyano-4′-hexylbiphenyl (6CB), tetrabutylammonium perchlorate (TBAP) as the supporting electrolyte, and a chiral dopant, (*R*)- or (*S*)-1,1′-binaphthyl-2,2′-bis[para-(*trans*-4-pentylcyclohexyl)phenoxy-1-hexyl] ether, to produce the corresponding thin-film polymers of (*R*)-PEDOT* and (*S*)-PEDOT*, respectively (Scheme 29) [73].

CD spectra of (*R*)-PEDOT* exhibited a positive Cotton effect, while (*S*)-PEDOT*displayed a negative effect above 320 nm, confirming main-chain helicity. Polarized optical microscopy (POM) revealed a double-spiral texture, indicating that single-handed twisted chains aggregate into 3D helical bundles. This approach (reported in 2004) demonstrated that the N*-LC matrix alone can induce macromolecular chirality without requiring chiral monomer substituents. for preparing chiral polymers.

**Scheme 30:**
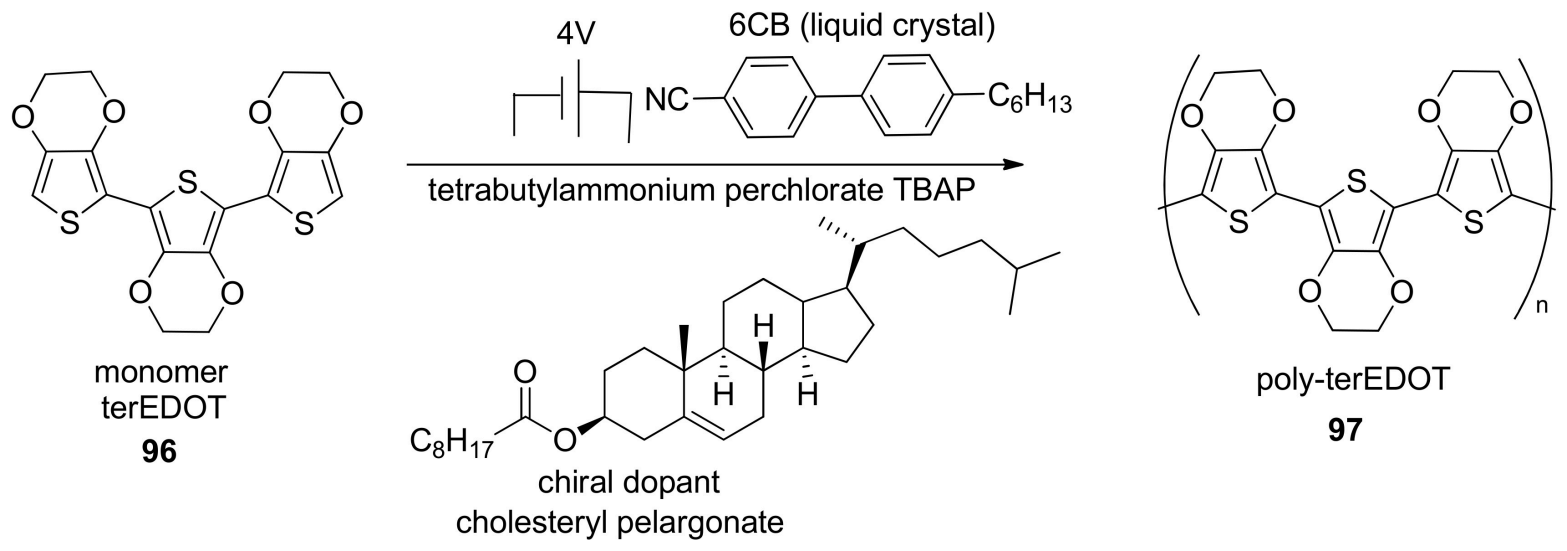
Electropolymerization of tertEDOT (**96**) using asymmetric reaction template.

The chirality of a nematic liquid crystal phase can be induced by adding cholesteryl pelargonate as a chiral dopant to liquid crystal (6CB) [74]. Within this medium, terEDOT (**96**) was electrochemically polymerized between ITO glass electrodes at 4.0 V to yield chiroptical PEDOT films (**97**) (Scheme 30). The CLC medium effectively templated polymer growth, inducing a one-handed helical aggregation. 

POM analysis revealed that the polymer **97** retained the host CLC fingerprint texture. SEM imaging showed fibrillar aggregates featuring thin, clockwise-twisted helical bundles, with coil-like fibrils forming vortices in the interstitial spaces. In the absorption spectra, the electrochemically doped PEDOT* film displayed a peak at 538 nm assigned to the π → π^∗^ transition of the main-chain. Increasing the oxidation potential weakened this band, giving rise to new peaks at 756 nm (polarons) and 1298 nm (bipolarons). In situ CD spectra showed reversible chiral electrochromism: the reduced state exhibited a bisignate Cotton effect from intermolecular exciton coupling, while upon oxidation, the intercalation of perchlorate counterions increased the interchain spacing, without disrupting the overall chiral packing.

**Figure 15:**
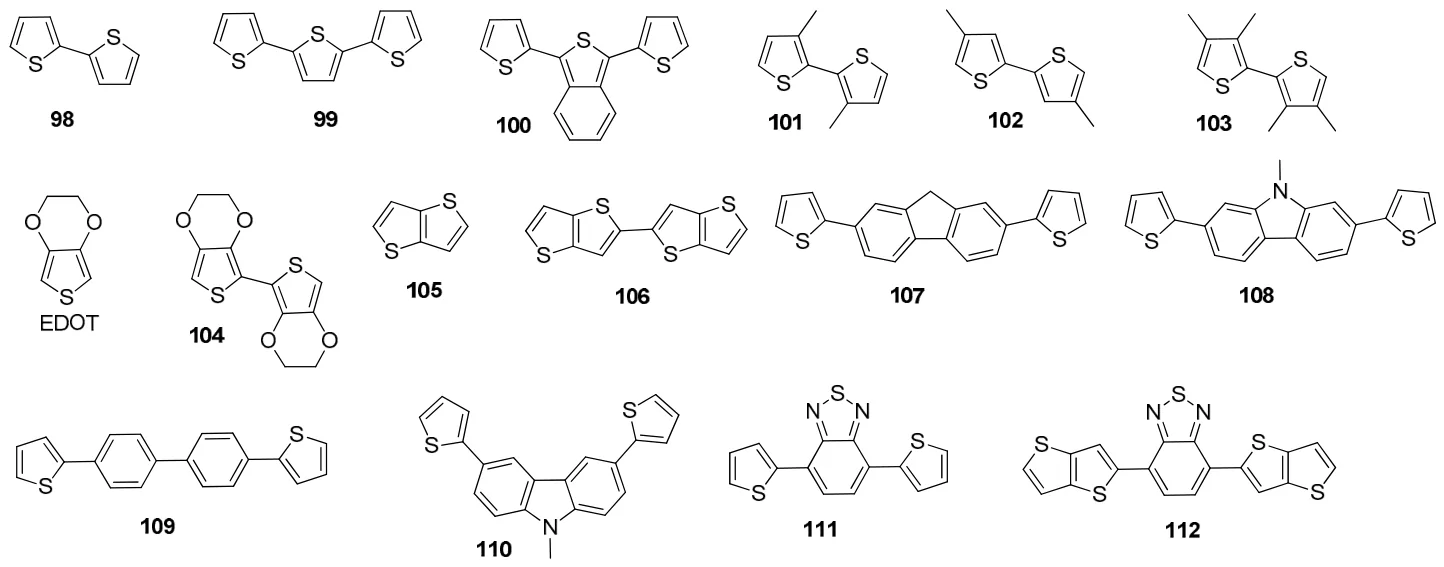
Heteroaromatic monomers.

The Goto group investigated the mechanism of chiral induction during asymmetric electropolymerization in a cholesteric liquid crystal (CLC) medium using EDOT and newly designed heteroaromatic derivatives **98**–**112** (Figure 15) [75,76].

**Figure 16:**
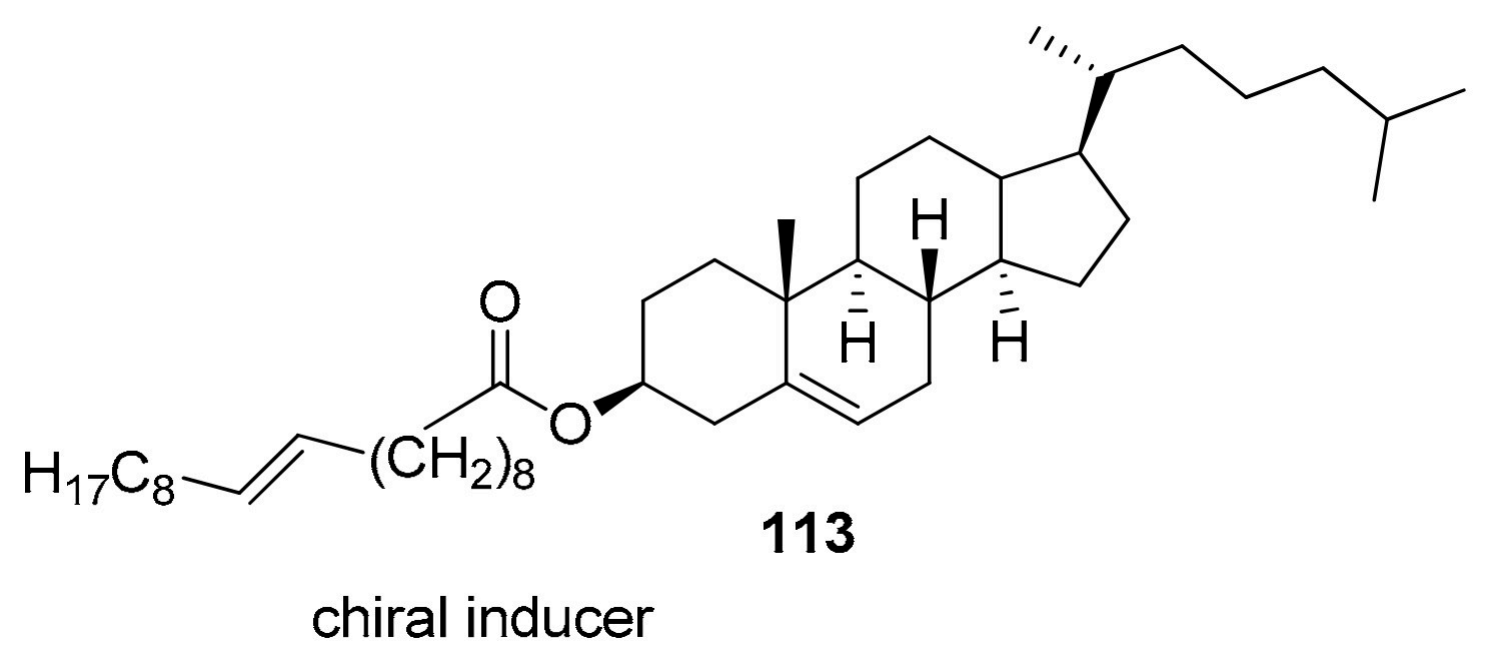
Structure of cholesteryl oleyl carbonate.

In these studies, cholesteryl oleyl carbonate **113** (COC, Figure 16) serves as a chiral dopant in 6CB due to its high helical twisting power and excellent compatibility. The mixture of chiral dopant **113**, 6CB, monomer, and TBAP form the electrolyte solution for asymmetric electropolymerization. Polymerization was initiated by applying 4 V DC across ITO electrodes for 10 min to 1 h. Rinsing off the CLC electrolyte solution with a hexane/acetone or methanol/acetone mixture yielded polymeric films on the anode surface [75]. The optical and chiral properties of the resulting polymers are summarized in Table 2. 

These characteristic optical and chiral variations among polymers sharing similar chemical backbones demonstrate that the choice of the parent monomer is critical for directing chiral assembly during CLC-templated asymmetric electrochemical polymerization. Furthermore, experiments using different chiral inducers (i.e., cholesteryl pelargonate (CP) and 4′-(4-hexyloxybenzoyloxy)biphenyl-4-carboxylic acid (*S*)-1-methylheptyl ester) with the same helical pitch yielded films with identical optical properties. This confirms that the helically aggregated polymer structures are templated not by the molecular chirality of individual dopants, but by the macroscopic helical molecular order of the bulk CLC phase.

The same research group reported that electrochemical polymerization under a magnetic field produces polymer films that exhibit linearly polarized electrochromism [77]. Polymer transcribes the magnetic-field-aligned liquid crystal (LC) order. 

**Scheme 31:**
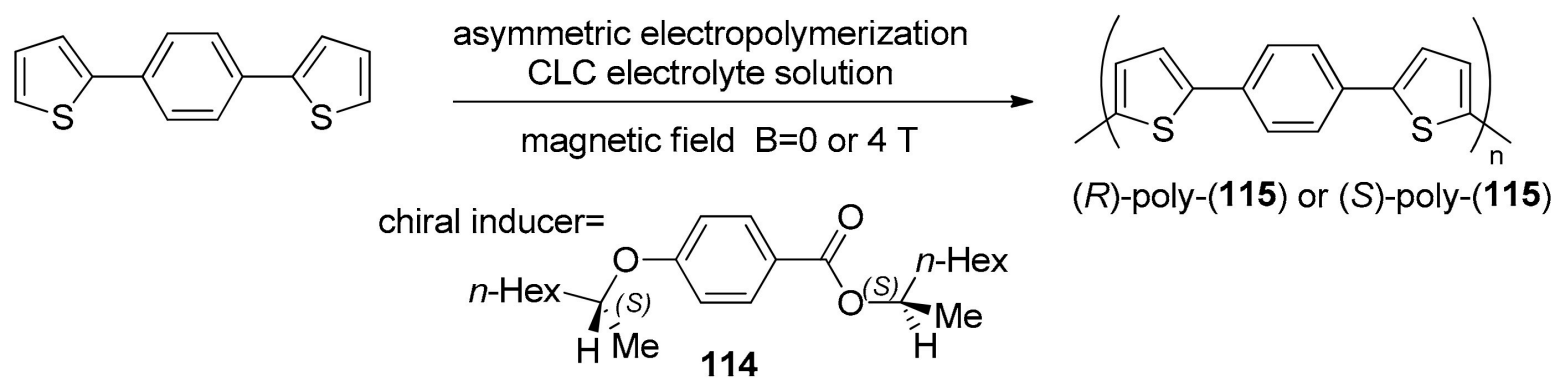
Synthesis of chiroptically active polymers **115** by electrochemical polymerization.

Later research utilized the chiral inducers (*R*,*R*)-**114** and (*S*,*S*)-**114**, having double stereogenic centers, for asymmetric electropolymerization in a cholesteric (CLC) medium (Scheme 31) [78]. The polymerization produced chiroptically active atropisomers of **115**, whose helical sense matched that of the CLC electrolyte.

**Figure 17:**
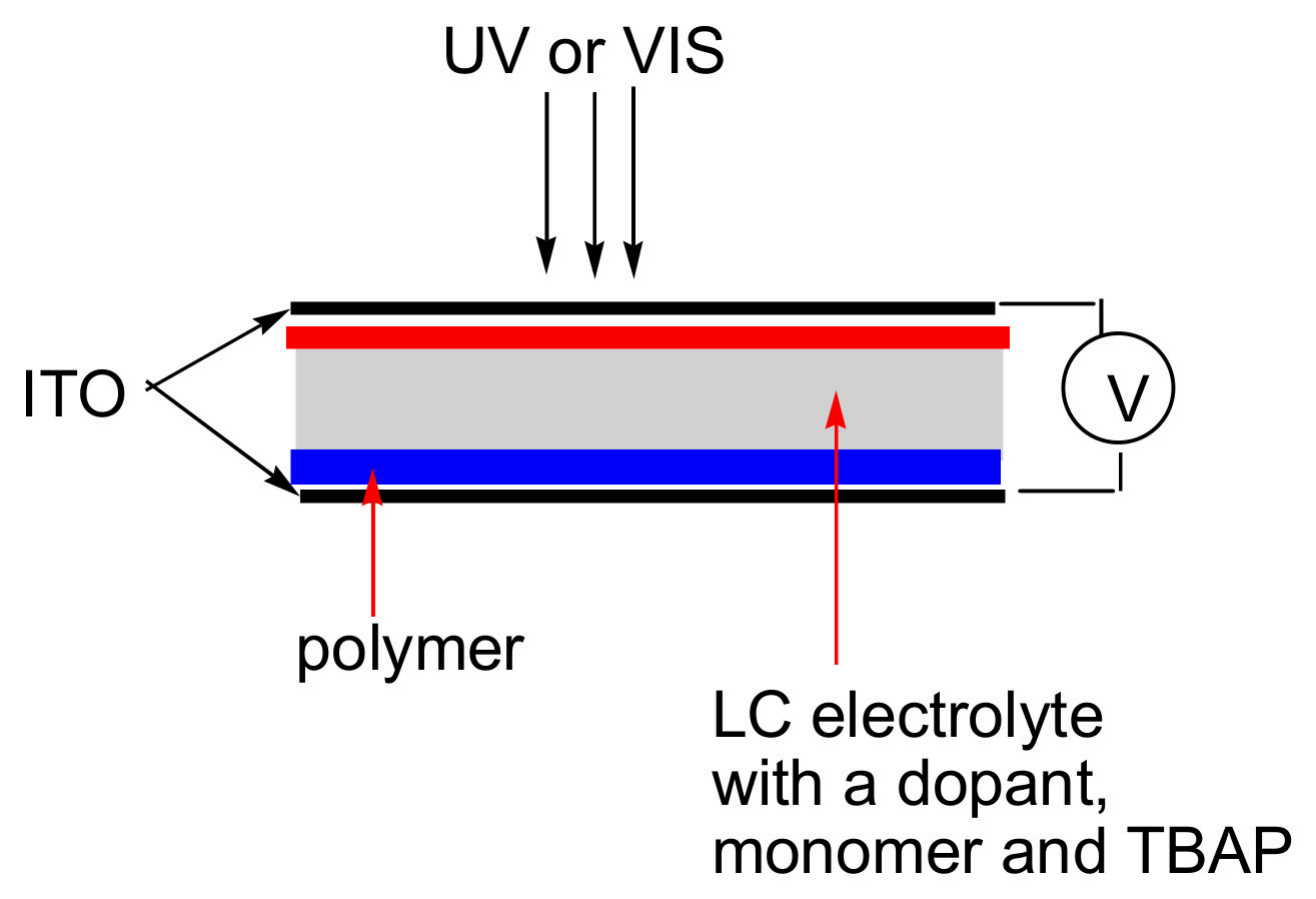
Schematic illustration of light-driven asymmetric electropolymerization.

Later, Goto and Kawabata developed a light-driven asymmetric electropolymerization of terEDOT (**96**) conducted in a liquid crystal (LC) electrolyte solution under UV or visible (Vis) light irradiation to tune the chiral environment (Figure 17) [79].

**Scheme 32:**
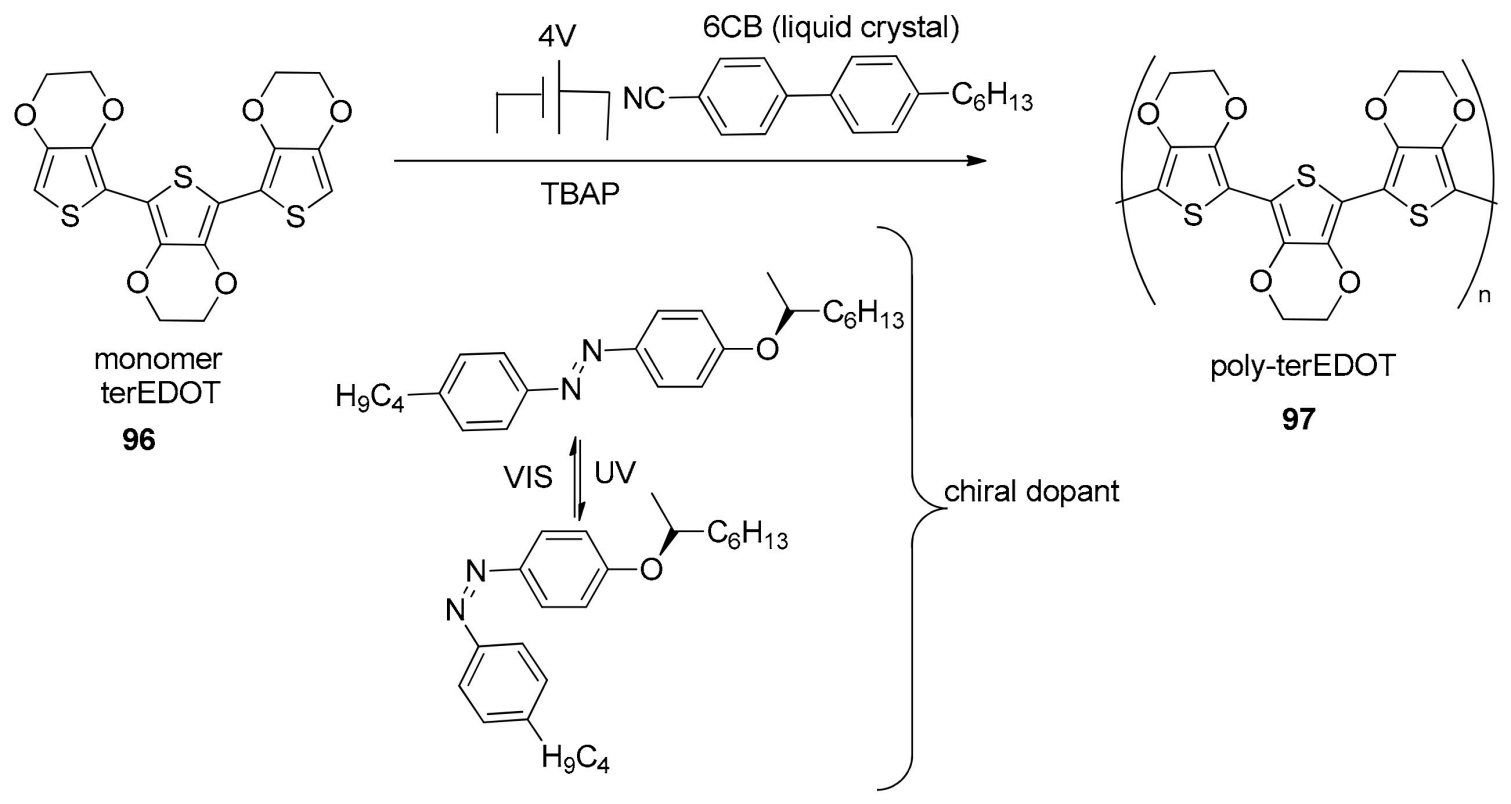
Light-driven asymmetric electropolymerization of terEDOT (**96**).

In this study, the selected LC undergoes a photo-induced phase transition triggered by the *trans*–*cis* isomerization of the azobenzene moiety within the chiral dopant. This photoisomerization alters both the structure and the chirality of the cholesteric LC electrolyte, enabling the tuning of chiroptical activity during polymerization to yield polymer **97** (Scheme 32). Optical texture monitoring during irradiation revealed a multi-step photo-induced transition: (1) a planar texture, (2) a shift in the helical axis direction, (3) a transition from the cholesteric to the nematic phase, and (4) a transition from the nematic to the isotropic phase. Repeated isomerization cycles under visible light ultimately produced a distinct fingerprint texture. 

**Figure 18:**
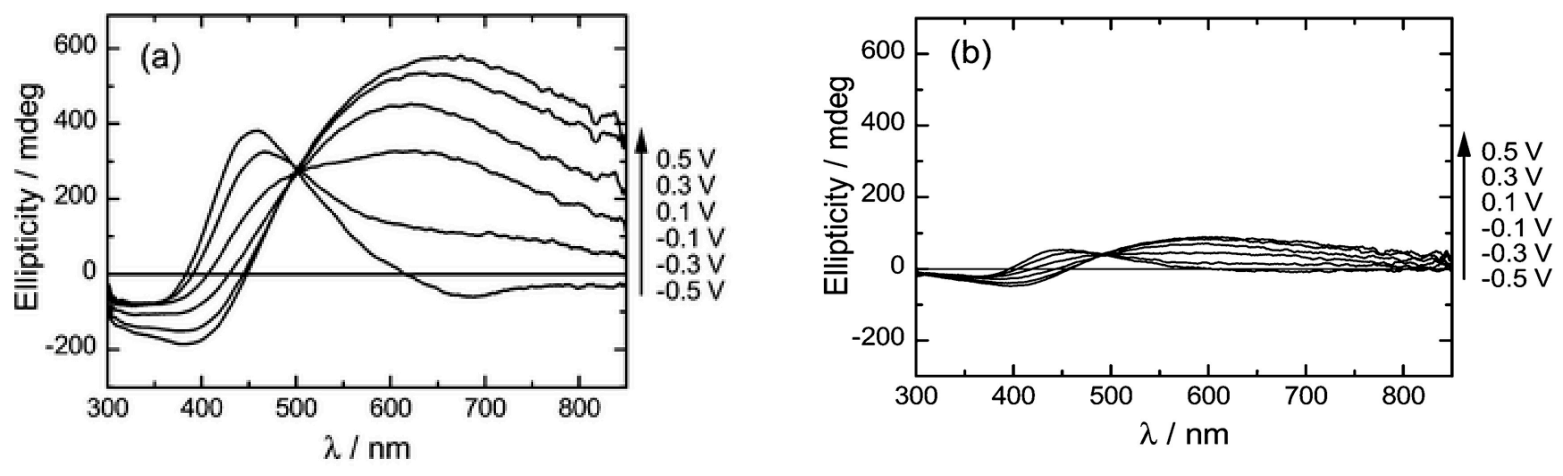
CD spectra of PterEDOTs at applied voltages from −0.5 V to +0.5 V. (**a**) PterEDOT prepared in cholesteric LC under Vis light (Poly(terEDOT) (Vis)). (**b**) Poly(terEDOT) prepared in cholesteric LC after UV light irradiation for 30 min (PolyterEDOT (UV30)). Adopted with a permission from: Hiromasa Goto, Kohsuke Kawabata; Light driven asymmetric polymerization: an approach for tele-control reaction. Polym. Chem. 2011; 2 (5): 1098–1106 [79] https://doi.org/10.1039/c0py00396d. Published by the Royal Society of Chemistry. © 2011 The Author(s).

At low voltage, the CD spectra showed a split-type bisignate profile, indicating exciton coupling from intermolecular chiral aggregation (Figure 18a). The polymer retained partial chiral aggregation even when oxidized, stabilized by the intercalation of perchlorate ions between the polymer backbones during the redox process. Polymerization under UV irradation (30 min) (Figure 18b) yielded much weaker CD signals than under visible light. This confirms that the UV-induced *cis*-dopant disrupted the chiral ordering of the cholesteric phase.

**Scheme 33:**
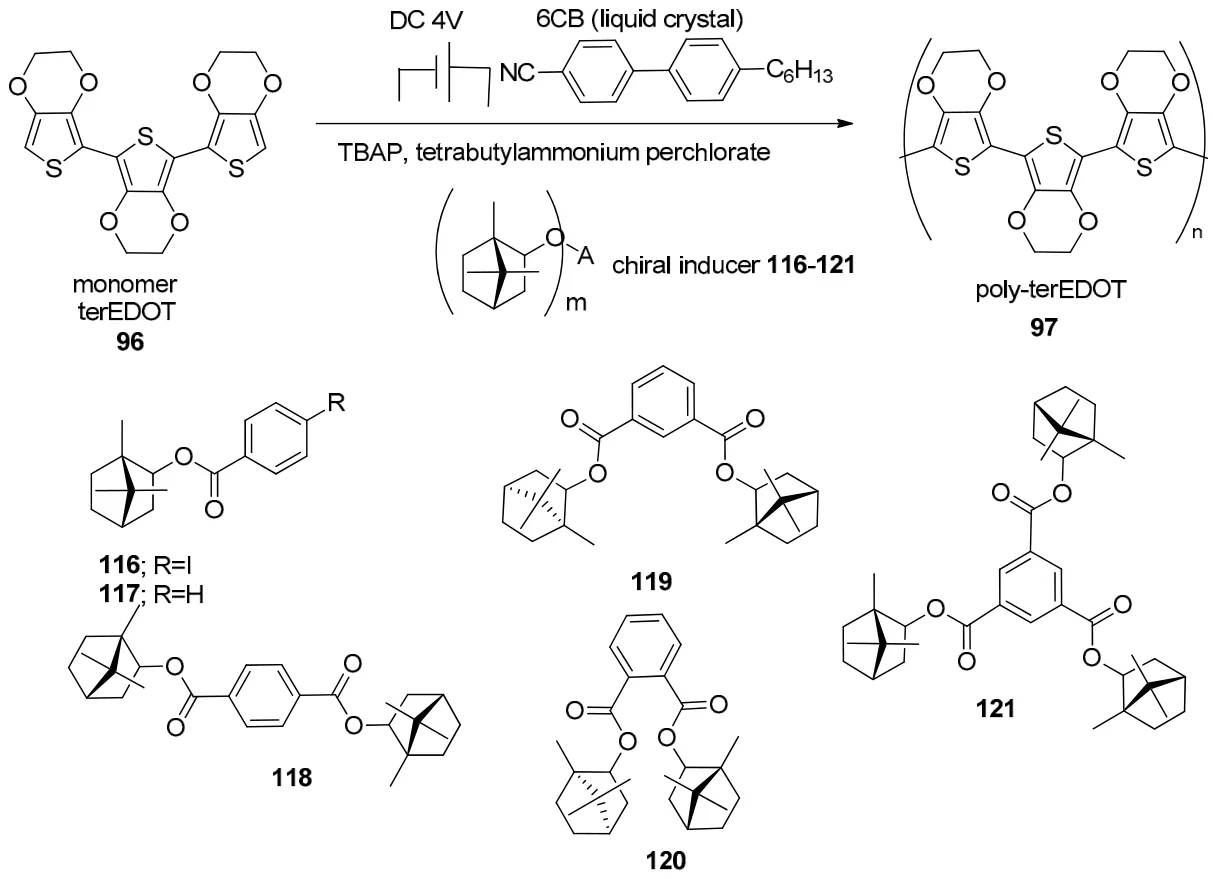
Asymmetric electropolymerization of terEDOT in the induced cholesteric LC electrolyte containing chiral inducers based on l-borneol **116**–**121**.

To produce chiral PEDOT* films **97** with helical backbones, Goto et al. developed an asymmetric electropolymerization strategy (Scheme 33) using achiral terEDOT **96** in a cholesteric liquid crystal medium, doped with l-borneol-derived chiral inducers [80,81]. Helical twisting power (HTP) along with macroscopic HTP (*β*_M_) evaluations showed that inducers **118** and **119** exhibited higher *β*_M_ values than **116** or **117**, indicating that a greater number of bornyl groups enhances twisting ability. Inducers **120** and **121** were less effective due to differences in host LC matrix affinity. 

Polarizing optical microscopy (POM) revealed a vortex structure in the film, mimicking the parent CLC fingerprint texture. Circular dichroism (CD) spectra of the polymers in their reduced state displayed a bisignate Cotton effect (Davydov splitting) indicating right-handed helical aggregation transcribed from the CLC template. The CD spectrum of the film in its oxidized state lacked a bisignate Cotton effect and exhibits broadly negative CD signals at long wavelengths, likely arising from the polaron band in the doped state. 

**Scheme 34:**
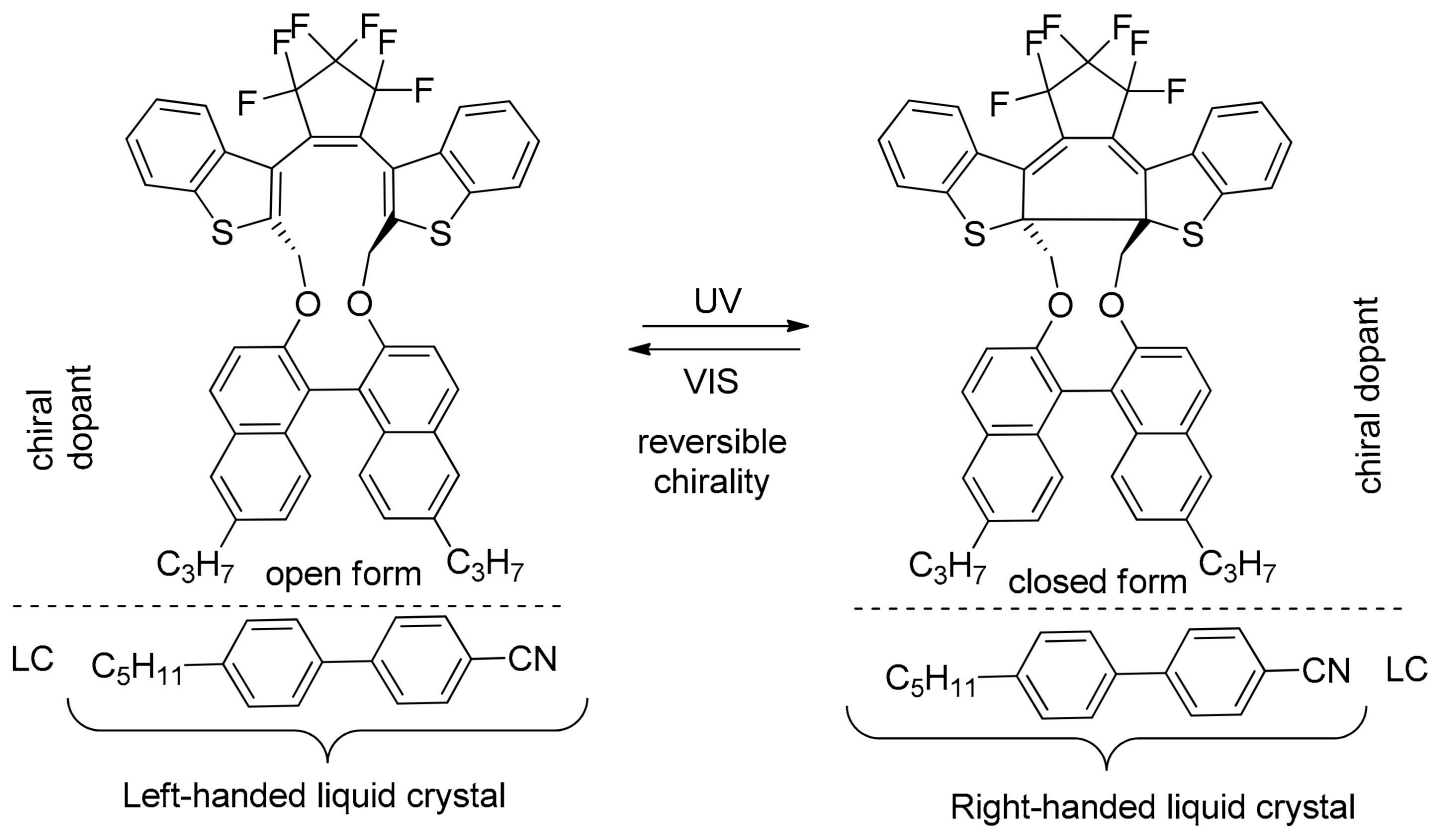
Chiral nematic liquid crystals based on 4-cyano-4′-pentylbiphenyl (5CB) containing photoresponsive chiral dopants.

Subsequent three-ring chiral inducers [82] were designed in order to achieve better twisting ability in asymmetric electrochemical polymerization. Recently, photoinvertible chiral liquid crystals combining a photoresponsive bis(benzothienyl)ethene unit and an axially chiral binaphthyl moiety were applied to the asymmetric polymerization of an EDOT dimer [22]. Reversible photoisomerization between the open form and the photostationary state (PSS) closed form inverted the LC matrix chirality under selective light irradiation (Scheme 34). CD spectra displayed positive or negative bisignate Cotton effects, demonstrating that the synthesized polythiophenes exhibited left- or right-handed helicity depending on whether the host matrix was in its open or closed PSS form.

**Scheme 35:**
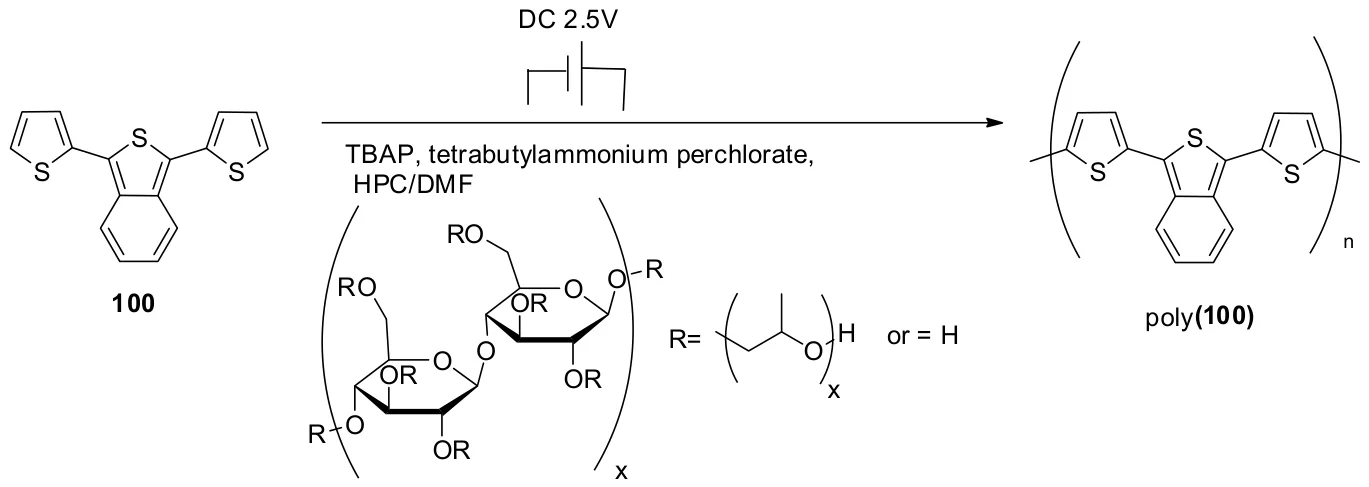
Asymmetric electrochemical polymerization of [thiophene]-[isothianaphthene]-[thiophene] monomer.

Electrochemical polymerization was also carried out in a hydroxypropyl cellulose/*N*,*N*-dimethylformamide system (HPC/DMF), a lyotropic liquid crystal serving as an electrolyte solution, to afford the chiral, electroactive polymer poly(**100**), despite that the thiophene-isothianaphthene-thiophene triad monomer **100** used had no stereogenic center (Scheme 35) [83].

The resulting polymer had a clear fingerprint texture in the polarized optical microscopic image. Because the HPC phase aggregates helically, its supramolecular chirality was transcribed onto the polymer film during electropolymerization. Upon electrochemical oxidation, a new polaron (radical cation) absorption band emerged at 960 nm. This band reversibly increased during oxidation and decreased during reduction, demonstrating stable, repeatable electrochromic behavior across doping/dedoping cycles.

**Scheme 36:**
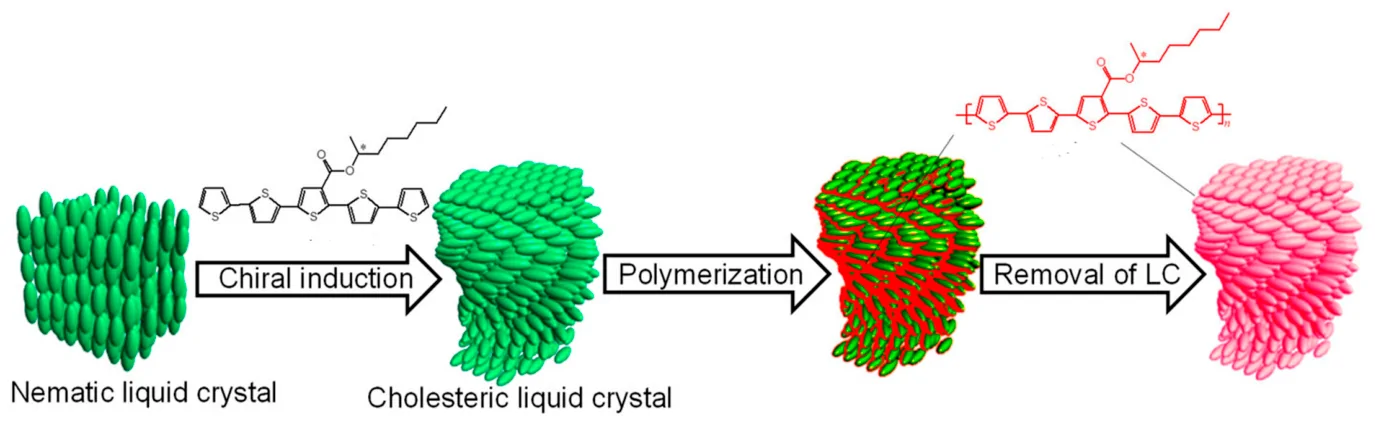
Chiral induction from nematic LC to cholesteric LC by chiral (+)-quinquethiophene, and its electrochemical polymerization. The insolubility of the polymer facilitates phase separation thus the resulting film of pure poly(quinquethiophene) with a helical structure was isolated after washing. Adopted with permission from: Goto, H. (2022). Reaction field induction self-amplification optical activity during polymerization in liquid crystal. *Molecular Crystals and Liquid Crystals*, *745*(1), 26–39. https://doi.org/10.1080/15421406.2022.2073421 [84]. Rights managed by Taylor & Francis.

More recently, Goto reported a strategy wherein a chiral monomer serves a dual function as both the chiral dopant to a liquid crystal (LC) matrix and the polymerizable unit [84]. By introducing a chiral quinquethiophene into a nematic liquid crystal (LC) matrix, the host mixture transformed into a chiral nematic (cholesteric) LC phase. Subsequent electropolymerization within this helically ordered matrix yielded a polythiophene film that imprinted the host’s helical supramolecular structure (Scheme 36).

Electrochemical analysis showed that poly(quinquethiophene) underwent reversible doping/dedoping with perchlorate ions, characteristic of electroactive polythiophenes. Circular dichroism (CD) spectroscopy revealed a distinct Cotton effect originating from the π → π* transitions along the polymer backbone. A bisignate CD profile shifting from a positive to a negative signal with decreasing wavelength suggested a clockwise (right-handed) helical orientation. 

##### Transcription of Chirality from Chiral Nematic Liquid-Crystalline Ionic Liquids

**Figure 19:**
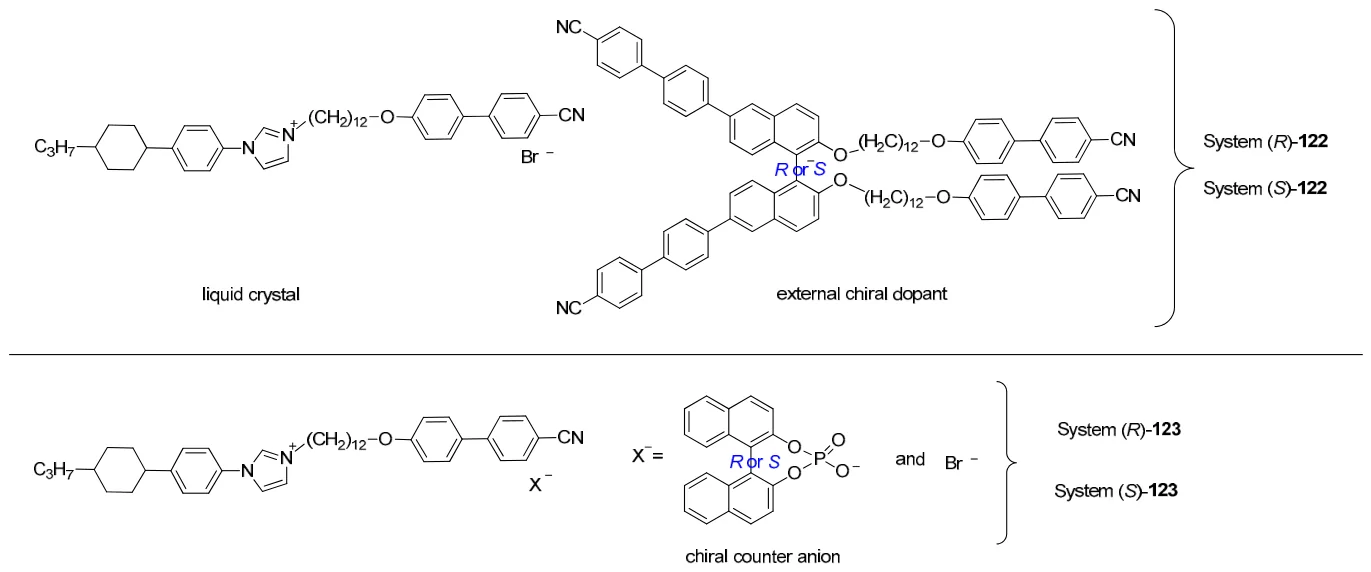
Two chiral nematic liquid-crystalline ionic liquids (N*-LCILs).

Chiral liquid-crystalline ionic liquids are utilized as functional media for asymmetric electrochemical polymerizations. To this end, two types of chiral nematic liquid-crystalline ionic liquids (N*-LCILs) were developed [72]: the first utilizes an axially chiral binaphthyl derivative as a chiral dopant (systems (*R*)-**122** and (*S*)-**122**, Figure 19), while the second incorporates a chiral binaphthyl phosphate anion directly into an imidazolium-based LCIL framework (systems (*R*)-**123** and (*S*)-**123**, Figure 19). The use of these N*-LCILs eliminates the need for additional supporting electrolytes or external chiral dopants during electropolymerization; due to their inherent ionic conductivity, the LCILs intrinsically function as both the reaction medium and the supporting electrolyte.

The synthesized N*-LCILs were utilized as both chiral solvents and supporting electrolytes for the asymmetric electropolymerization of bi- and terEDOT, yielding helical poly(3,4-ethylenedioxythiophene)s (H-PEDOTs) with helically π-stacked chains and single-handed fibril bundles. Morphological analysis via scanning electron microscopy (SEM) revealed that the handedness of the helically π-stacked structures matched that of the parent N*-LCIL, whereas the macroscopic twisted and spiral morphologies were opposite to those of the template. CD spectra of the neutral (*R*)- and (*S*)-H-PEDOT films displayed negative and positive bisignate Cotton effects, respectively, within the π → π* transition region of the conjugated chain, confirming the formation of left- and right-handed helically π-stacked structures. 

### 3.3. The Synthesis of Copolymers and Composites and Their Characterization

#### 3.3.1. Nickel-Catalyzed Chain-Growth Polymerization (GRIM/Kumada Methods)

Grignard metathesis (GRIM), or Ni-catalyzed Kumada catalyst-transfer polycondensation (KCTP) represents a controlled/living chain-growth strategy applied to chiral block copolymer synthesis. 

**Scheme 37:**
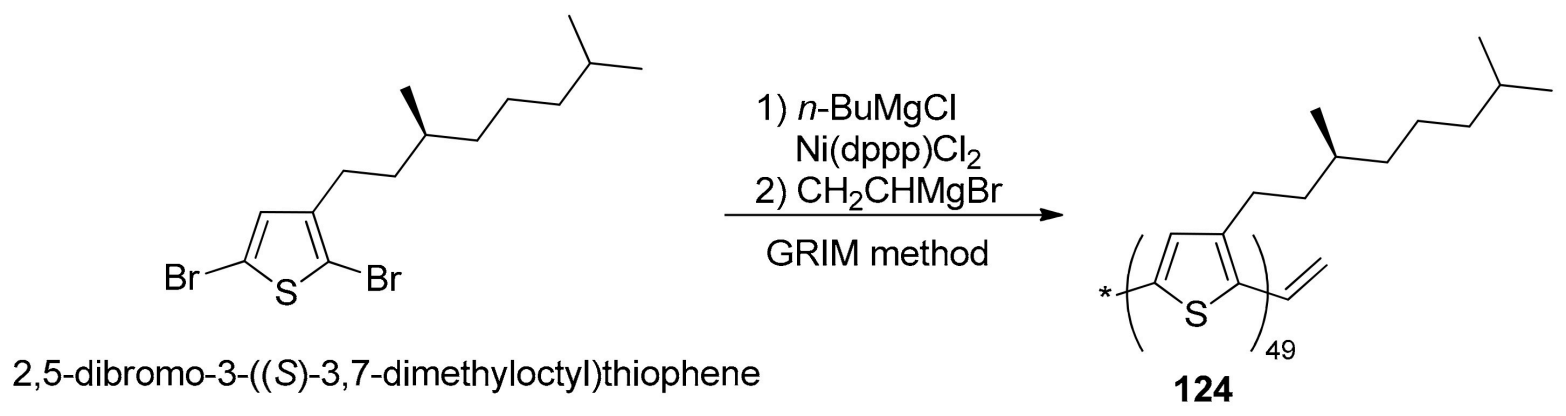
Synthesis of hydrophobic polymeric block **124**.

**Scheme 38:**
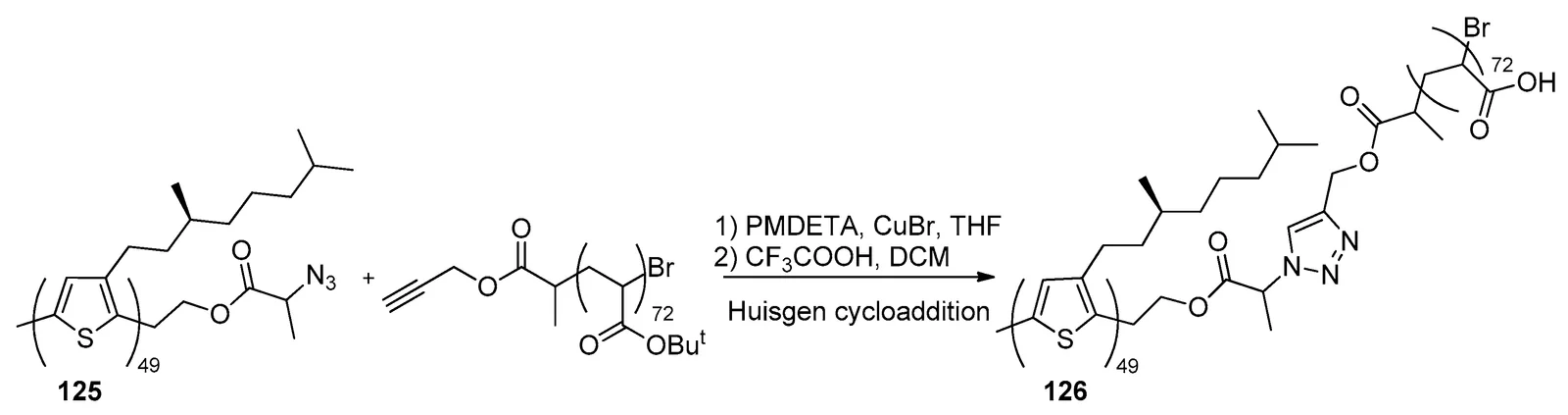
Synthesis of amphiphilic block copolymer **126**.

Kumada catalyst-transfer polycondensation (KCTP) was used to synthesize a hydrophobic polymeric block **124** consisting of regioregular head-to-tail (HT) *β*-substituted polythiophene (Scheme 37). Following the introduction of a terminal azide group, this block (*S*)-**125** was subsequently conjugated to hydrophilic poly(acrylic acid) chains to yield an amphiphilic block copolymer **126** (Scheme 38) [85]. 

The copolymer with chiral polythiophene blocks **126** self-assembles into stable micelles upon adding water to its THF solution and exhibits an induced circular dichroism (ICD) due to the formation of supramolecular π-stacked chiral aggregates within the micellar core. These aggregates remained stable without precipitation for over 5 days, whereas homopolymer (*S*)-**125** in a poor solvent mixture (CHCl_3_/methanol (1/1, *v*/*v*)) precipitated rapidly, losing over half of its CD intensity within 24 h. This indicates that micellar core encapsulation effectively stabilizes chiral supramolecular assemblies.

**Scheme 39:**
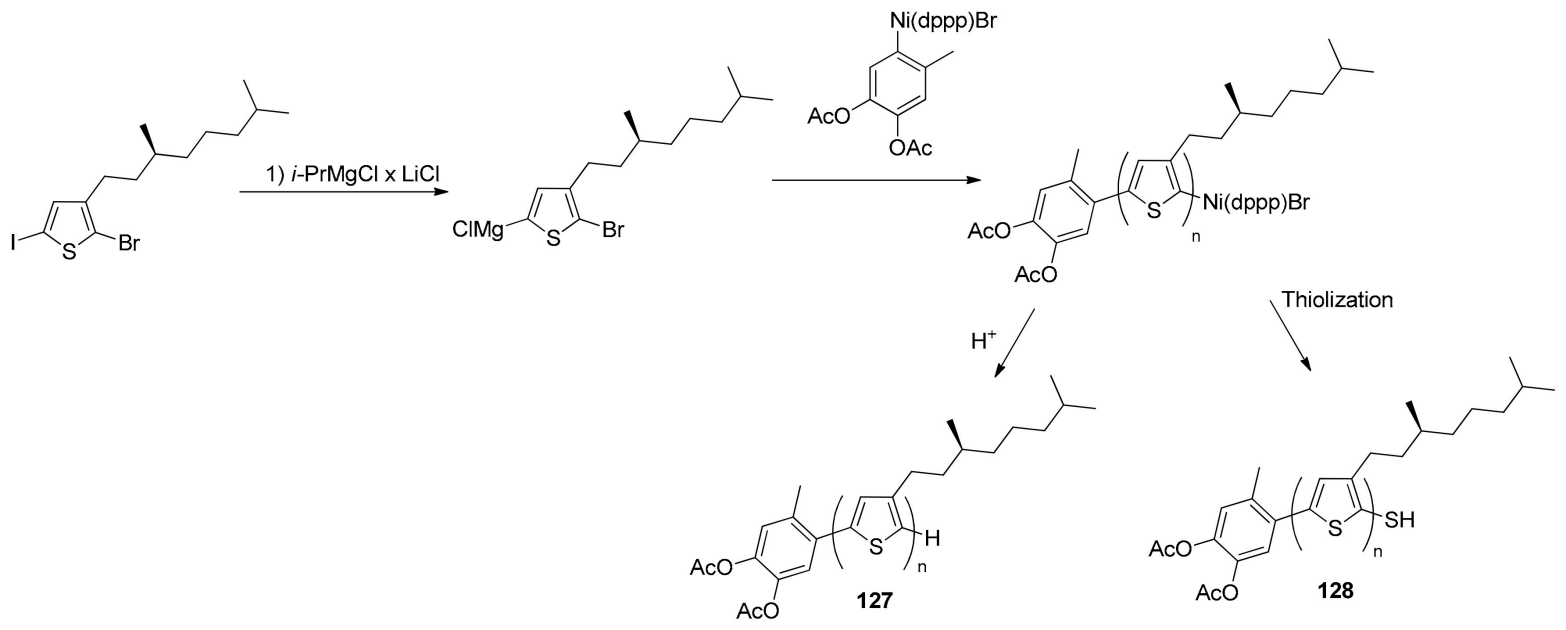
Synthesis of chiral α,ω-bifunctional polythiophenes.

**Scheme 40:**
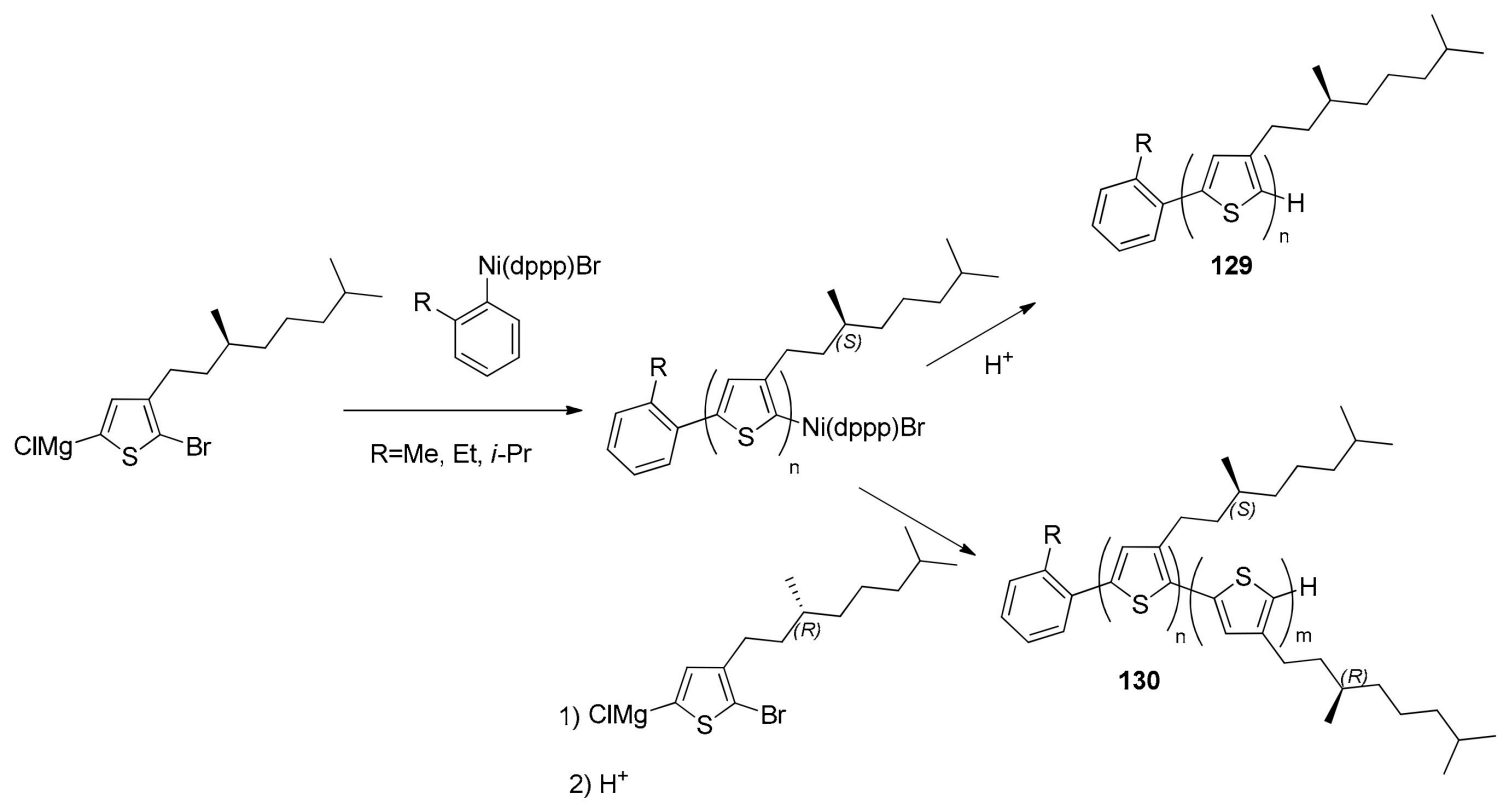
Synthesis of polythiophenes **129** and **130**.

Kumada catalyst-transfer polycondensation (KCTP) was next employed to synthesize chiral α,ω-bifunctional polythiophenes by capping poly(3-((*S*)-3,7-dimethyloctyl)thiophene) with a 4-methylcatechol moiety at the α-terminus **127** and a thiol functionality at the ω-terminus **128** (Scheme 39) [86], or to obtain a series of phenyl end-capped analogues **129** and **130** (Scheme 40) [87]. The latter studies established that specific end-capping groups directly influence the chiral self-organization of the resulting copolymers.

**Figure 20:**
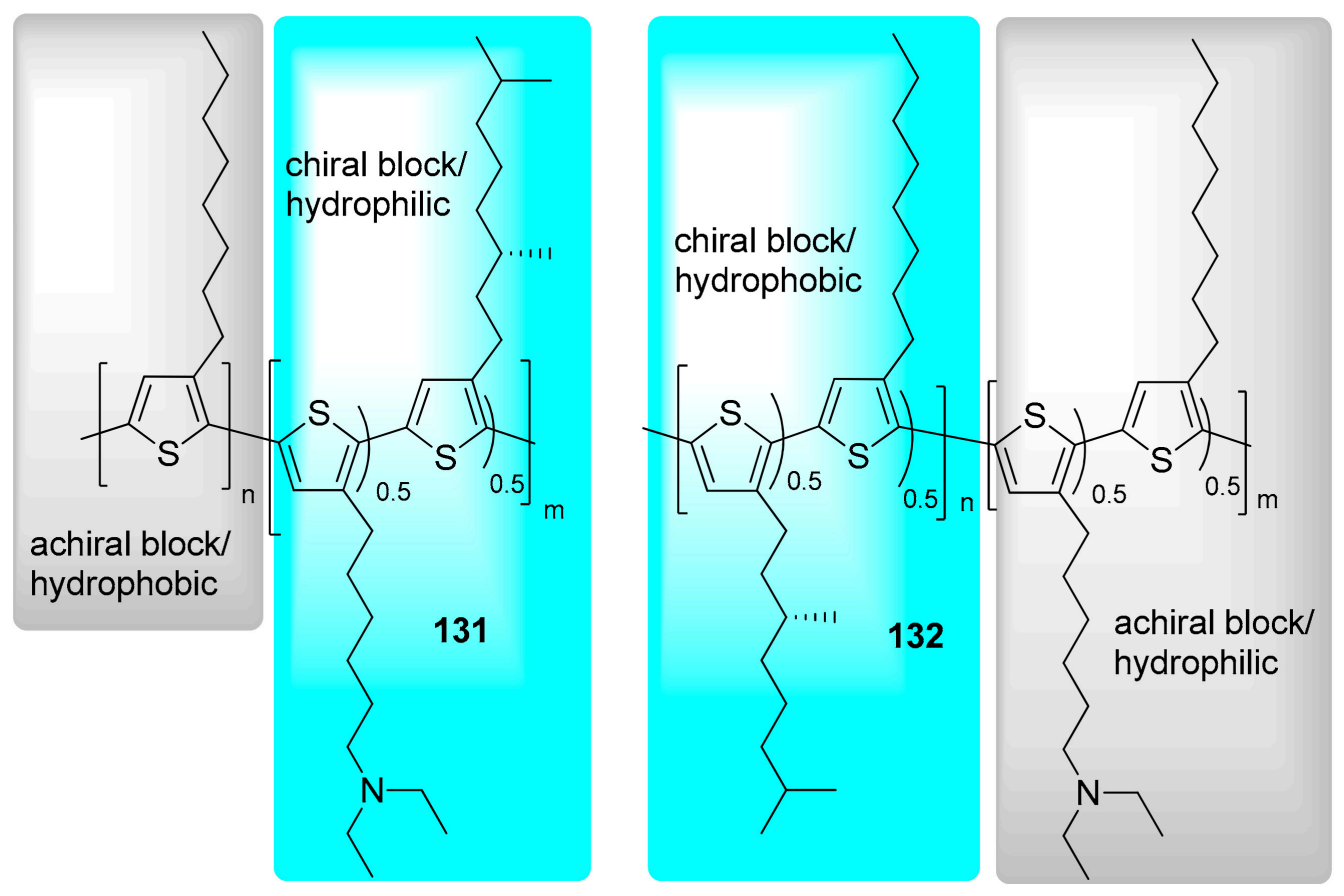
Amphiphilic block-copoly(thiophene)s **131** and **132**.

Similarly, amphiphilic block-copoly(thiophene)s **131** and **132** (Figure 20) bearing hydrophobic and hydrophilic blocks were synthesized via a one-pot Kumada cross-coupling using (*o*-tolyl)-Ni(dppp)Br as the initiator. The chirality was ensured by the presence of a chiral 3-((*S*)-3,7-dimethyloctyl)thiophene monomer facilitating chiroptical evaluation of polymer aggregation [88]. 

**Scheme 41:**
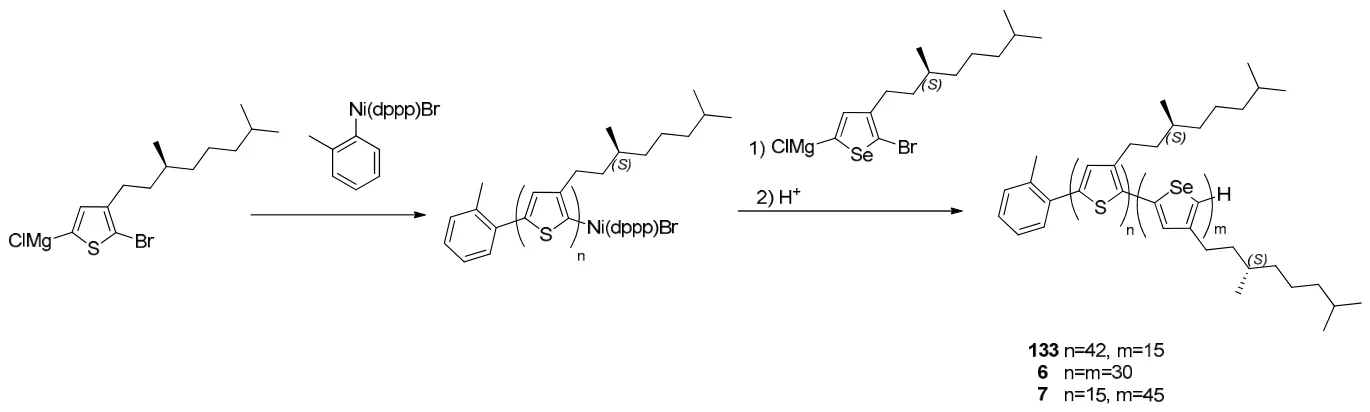
Synthesis of various poly(thiophene)-*b*-poly(selenophene) copolymers.

The identical KCTP methodology was expanded to synthesize various poly(thiophene)-*b*-poly(selenophene) copolymers **133**, **6**, **7** [23] (Scheme 41) enabling the study of the chiral expression and co-crystallization behavior of mixed-heteroatom block structures. 

However, synthesizing block copolymers whose individual blocks require two distinct mechanistic pathways is typically tedious and time-consuming. A more direct approach uses a single catalyst for a one-pot, living block copolymerization [89].

**Scheme 42:**
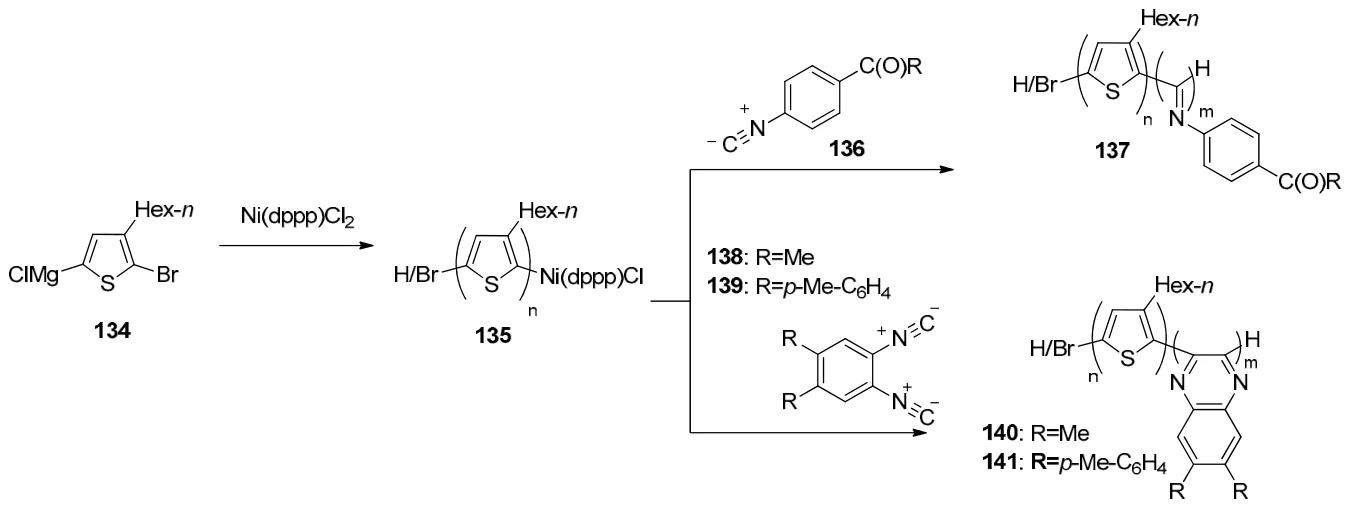
Synthesis of block copolymer **137**, **140** and **141**.

For example, a *π*-conjugated polymer macroinitiator **135** (Scheme 42) synthesized from 2-bromo-3-hexyl-5-chloromagnesiothiophene (**134**) via Kumada catalyst-transfer living polymerization was directly coupled with monomer (**136**) to yield the block copolymer **137** or with monomers **138** or **139** to afford **140** and **141**, respectively.

#### 3.3.2. Palladium-Catalyzed Cross-Coupling Polycondensations (Stille and Suzuki)

**Scheme 43:**
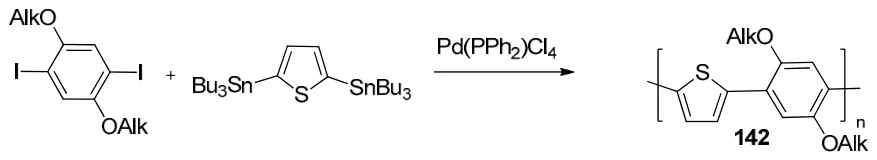
Synthesis of copolymers consisting of thiophene and dialkoxyphenyl units.

This section highlights step-growth polymerizations utilized to synthesize alternating copolymers of thiophene and various aromatic components, focusing on how spatial and structural arrangements dictate backbone chirality. In 1993, Stille cross-coupling was established as a powerful synthetic tool when it was first used to polycondense alternating copolymers **142** consisting of thiophene and dialkoxyphenyl units (Scheme 43) [90,91]. 

**Scheme 44:**
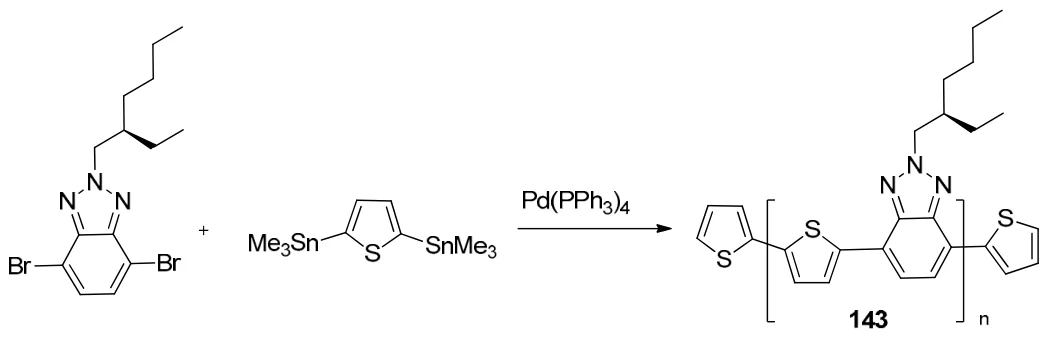
Synthesis of benzotriazole-thiophene copolymer.

Utilizing this classic methodology, a benzotriazole–thiophene copolymer **143** functionalized with chiral (*S*)-2-ethylhexyl substituent was prepared (Scheme 44) [92]. CD and optical absorption spectroscopies demonstrated that this polymer forms chiral aggregates even at exceptionally low concentrations in dichlorobenzene.

**Scheme 45:**
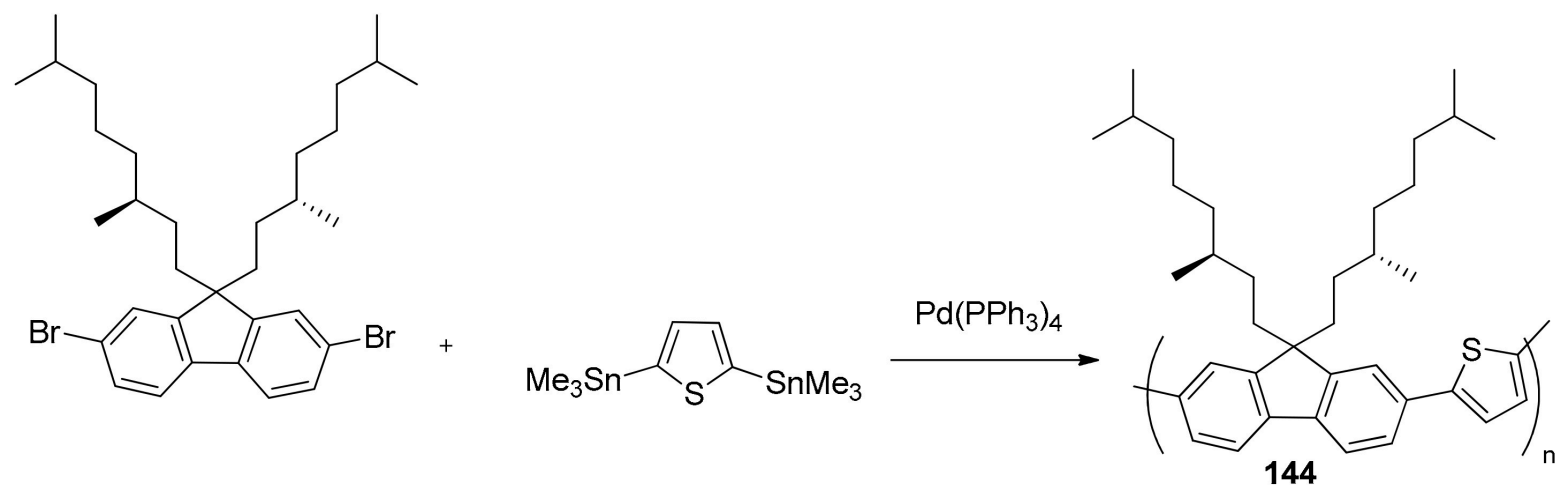
Synthesis of fluorene-thiophene based copolymer **144**.

About a decade ago, the fluorene–thiophene-based chiral copolymer **144** has been synthesized *via* Stille coupling of 2,7-dibromo-9,9-bis((*S*)-3,7-dimethyloctyl)fluorene with 2,5-bis(trimethylstannyl)thiophene (Scheme 45) [93]. Copolymer **144** showed unusually high optical activity in pristine and annealed thin films as demonstrated by intense circular dichroism signals.

Compared to polyfluorene homopolymer, the absorption band of copolymer **144** exhibited large bathochromic spectral shifts; which pointed out that thiophene, as a strongly electron-donating unit, can tune the HOMO/LUMO levels significantly narrowing the energy bandgap.

**Scheme 46:**
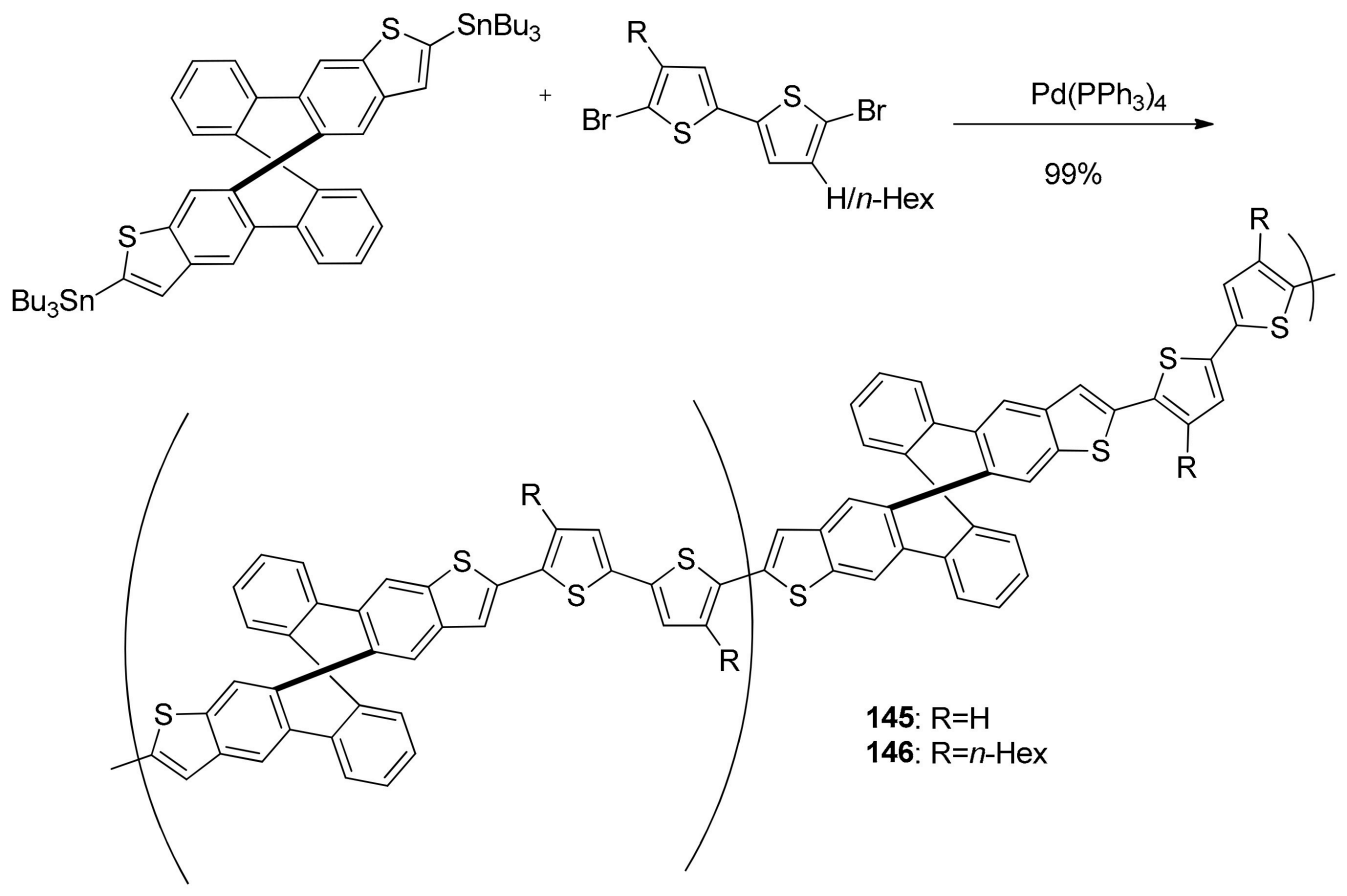
Synthesis of copolymers **145** and **146**.

Stille cross-coupling was used to conjugate a bithiophene unit with a *C*_2_-chiral thiophene-fused 9,9′-spirobifluorene, yielding copolymers **145** (unsubstituted) and **146** (hexyl-substituted), designed as switchable helical foldamers [94] (Scheme 46). 

It was expected that redox reaction would alter the planarity of bithiophene spacer units along the main chain, enabling a redox-driven conformational switch of the helical foldamers.

**Scheme 47:**
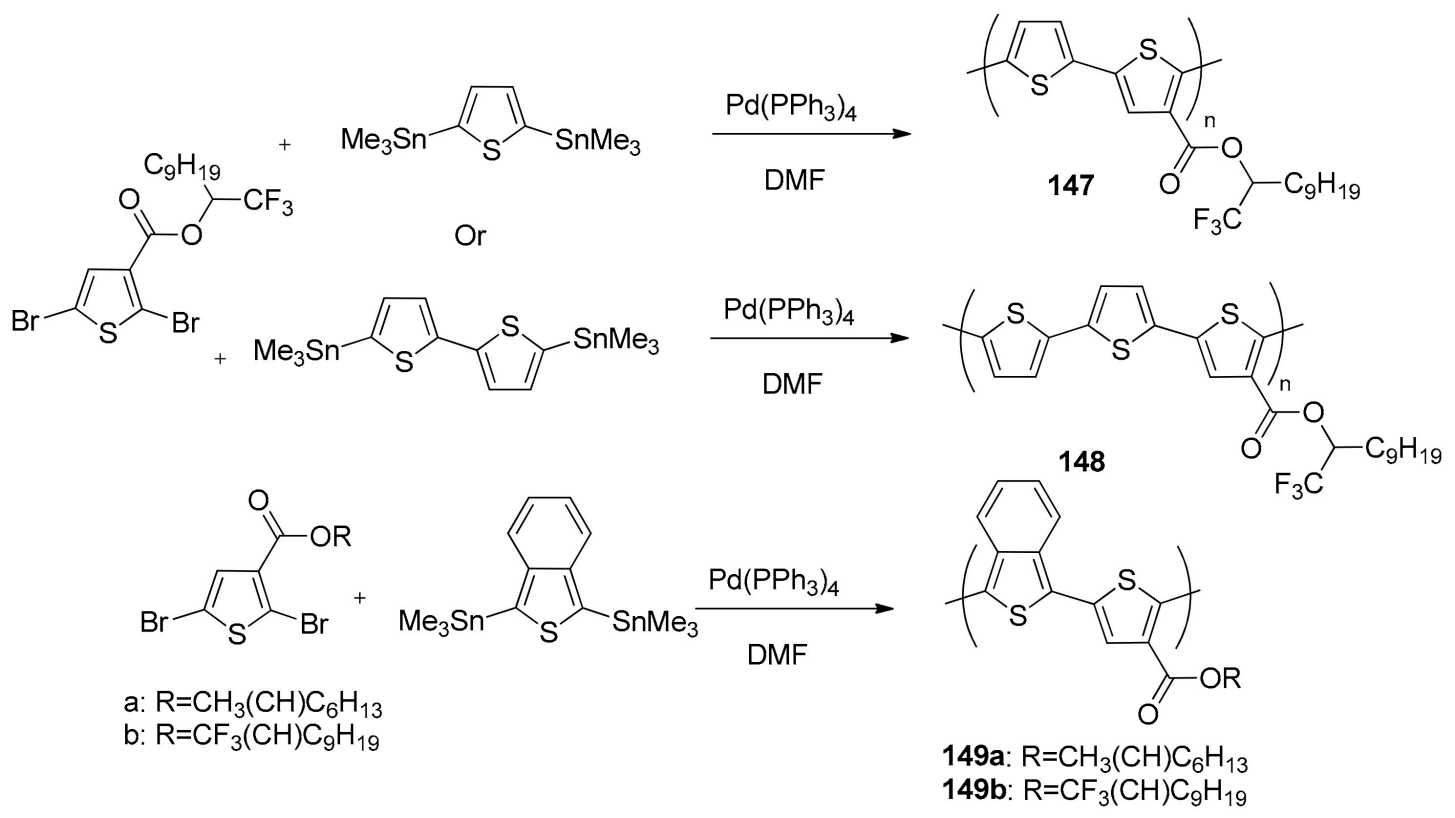
Migita-Kosugi-Stille type coupling based copolymerization yielding chiral thiophene-thiophene and isothianaphthene-thiophene copolymers.

Through-bond chirality transfer was subsequently analyzed in chiral thiophene–thiophene (**147**, **148**) and isothianaphthene–thiophene (**149a**,**b**) copolymers [95] synthesized via Migita–Kosugi–Stille cross-coupling (Scheme 47).

The polymer **147** showed a distinct, split-type Cotton effect in in its cast-film CD spectrum with a zero-crossing point at 447 nm matching its absorption maximum, indicating a 3D, single-handed helical π-stacked network. Other polymers in this series showed negligible chiroptical activity.

**Scheme 48:**
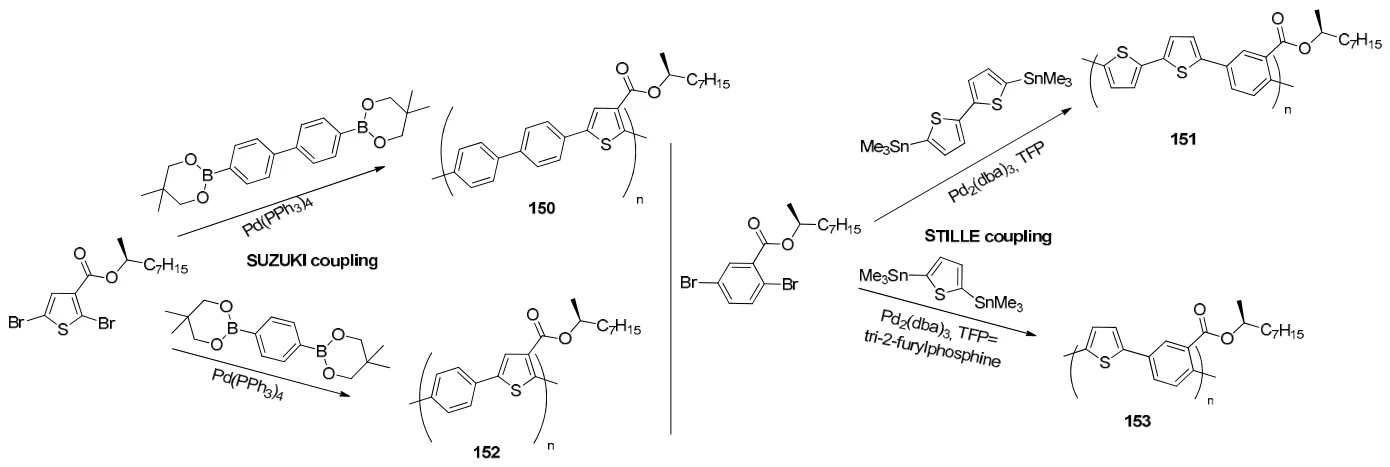
Synthesis of copolymers via Suzuki and Stille coupling.

Thiophene-based copolymers containing biphenyl and phenyl units decorated with chiral nonyloxycarbonyl side chains were synthesized via Suzuki or Stille cross-coupling (Scheme 48) [96].

Copolymers that have three aromatic rings in their repeat units: **150** and **151** exhibited bisignate Cotton effects in the CD spectra of both solutions and films suggesting the formation of tightly packed, interchain helical π-stacks. According to the exciton coupling theory, the copolymer (*R*)-**151** with a negative bisignate Cotton effect in the CD spectrum has a left-handed helical sense (M-helicity), whereas (*R*)-**150,** with a positive bisignate Cotton effect, adopts a right-handed helical sense (P-helicity). The authors proposed that chiral substituent position (phenylene vs. thiophene attachment) dictates the helical sense of π-stacked architecture, regardless of identical chiral configurations. Furthermore, these polymers exhibited RGB-colored fluorescence with quantum yields from 0.04–0.26 and showed circular polarization in their fluorescence bands. both in solution and solid-state. Polymer **152** consisting of two aromatic rings in the repeat unit, exhibited monosignate Cotton effect whereas **153** showed a slightly split Cotton effect, however, the CD intensities of the Cotton effects in both cases were very weak. The monosignate Cotton effect reflected through-bond chirality transfer without supramolecular helical π-stacking due to high solubility in chloroform. 

**Scheme 49:**
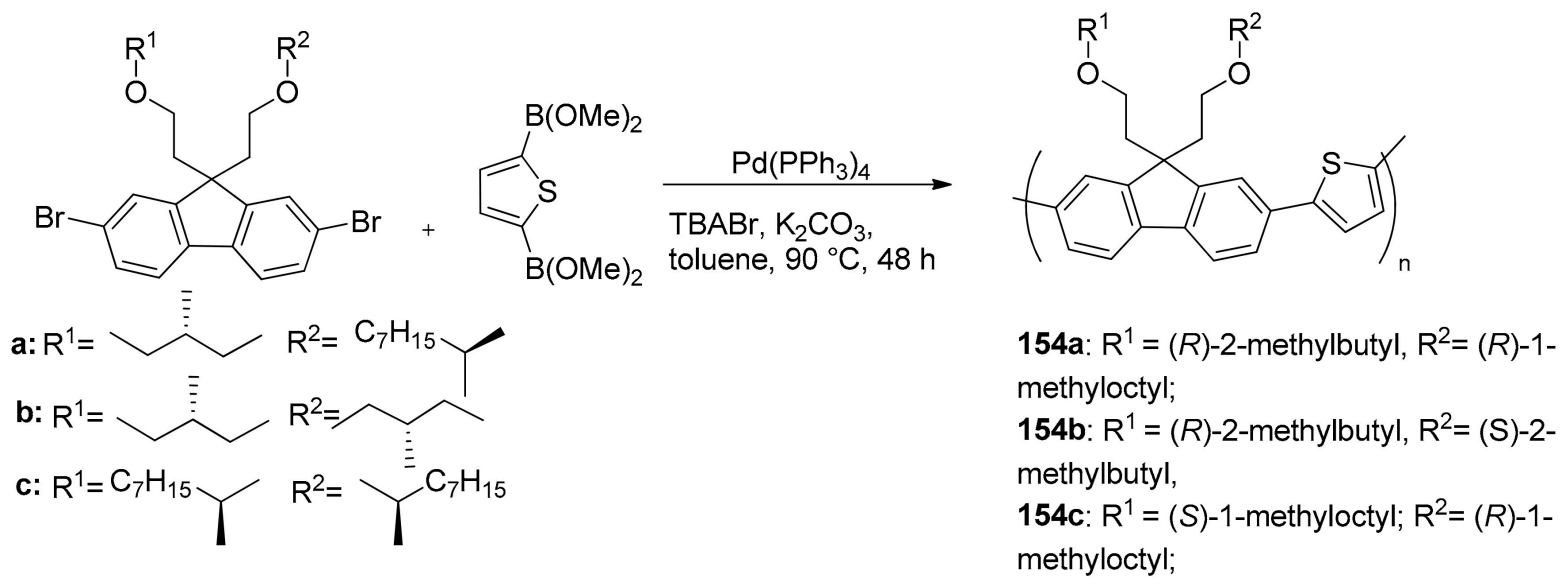
The approach to chiral poly(fluorene–thiophene) copolymers by Suzuki cross-coupling based polymerization.

A series of chiral polyfluorene–thiophene derivatives (**154a**, **154b** and **154c**) incorporating chiral side chains on the fluorene units was synthesized via Suzuki coupling reaction. The cross-coupling of chiral dibromofluorene monomers and thiophene-2,5-diboronic acid dimethyl ester successfully afforded the corresponding copolymers in 50–70% yields (Scheme 49) [97]. 

It was found that the new copolymers showed highly efficient CD and CPL signals in the green spectral region. Spin-coated films of **154b** and **154c** showed a relatively small Cotton effect in the π → π* transition region. Notably, the chiral substituent type controlled higher-order twisting direction: spin-coated films of **154b** self-assembled in an anticlockwise (M-type) configuration, whereas **154c** and **154a** assembled into clockwise (P-type) helical architectures. 

**Scheme 50:**
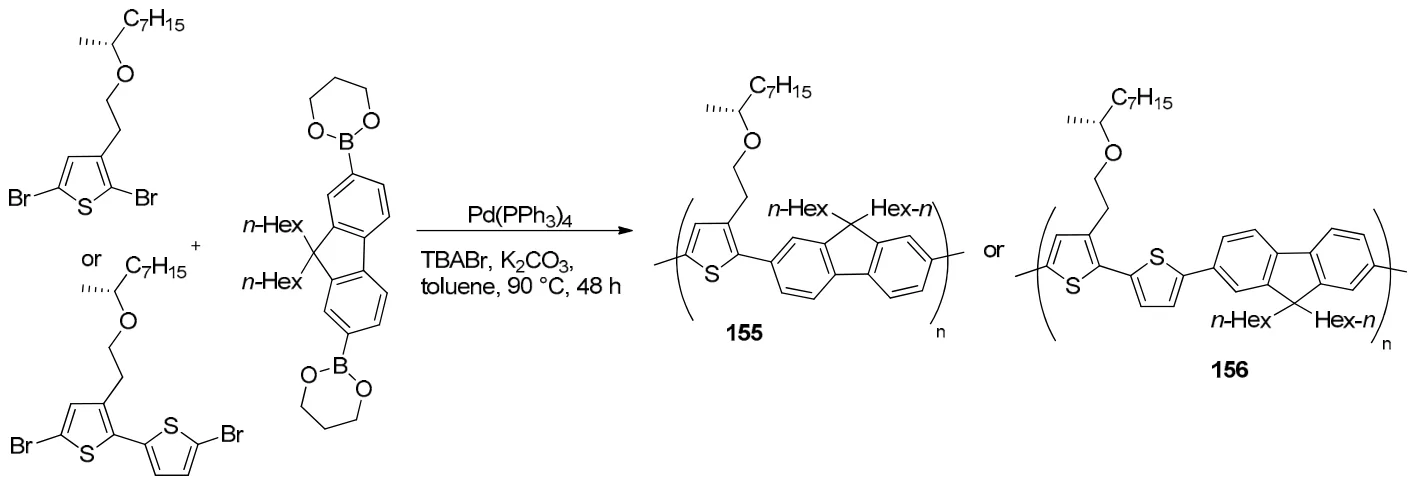
Synthesis of fluorene-based copolymers by the Suzuki coupling method.

Similarly, two fluorene based copolymers linked with thiophene or bithiophene bearing chiral side chains, **155** and **156**, were obtained by Suzuki coupling (Scheme 50) [98]. 

Circular dichroism measurements were conducted in methanol/chloroform mixed solutions and **155** and **156** exhibited strong bisignate Cotton effects. Characterization by X-ray diffraction and CD confirmed that variations in the structural spacer length significantly alter the orientation and configuration of the main chains.

**Scheme 51:**
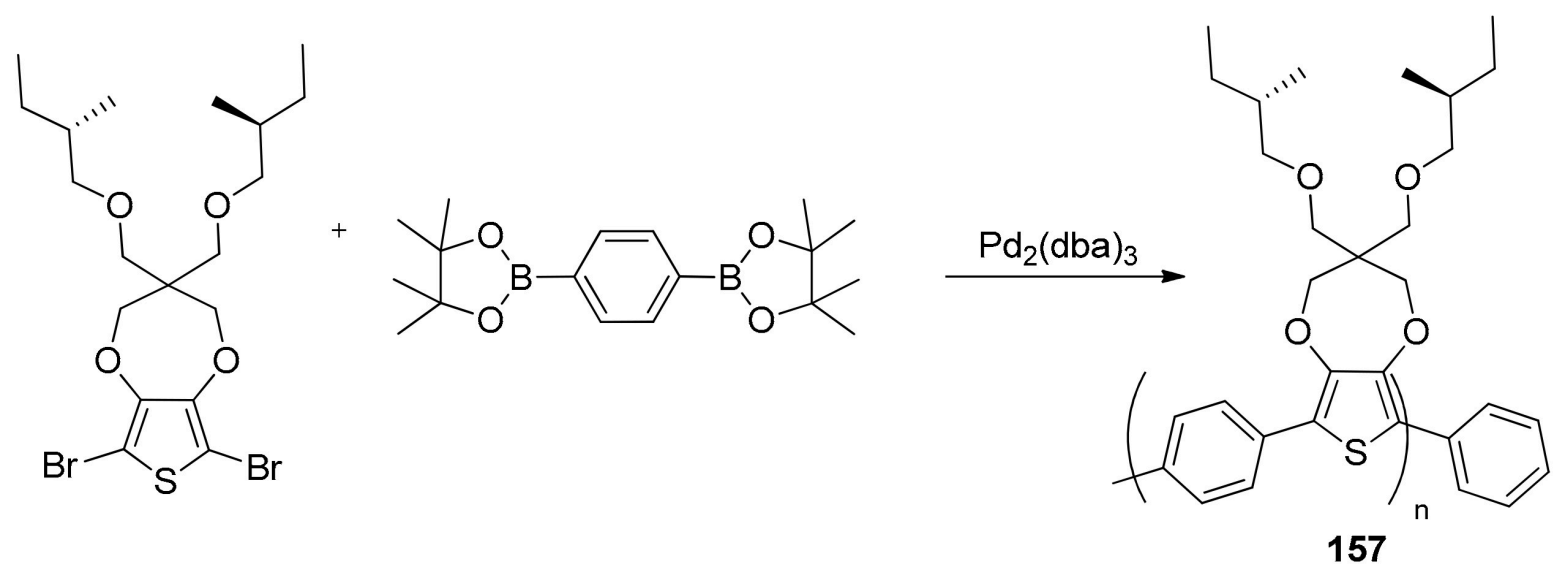
Synthesis of copolymer **157**.

Propylenedioxythiophene-phenylene (ProDOT-Ph) copolymer **157** bearing a chiral alkyl substituent directly on its propylene bridge was obtained via Suzuki cross-coupling (Scheme 51) [99].

The thin film of compound **157** was coated by annealing at 120 °C to optimize the chiral assembly and demonstrate its electrochromic, electrofluorochromic, and chiroptical switching. The films displayed high coloration efficiency, transitioning reversibly between yellow (neutral) and blue (oxidized) states with concomitant fluorescence switching (quantum yield shifting from 3.8% to 0.21%). In solution, chiroptical characterization by CD spectroscopy showed a strong negative bisignate Cotton effect in the absorption for the π → π* transition of the polymer, while thin-film optical performance depended strongly on thickness, with annealed films outperforming pristine films.

**Scheme 52:**
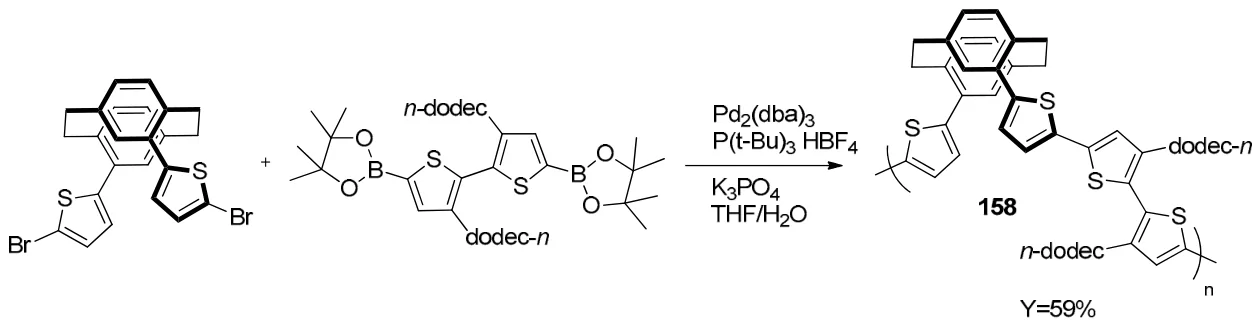
Synthesis of copolymer **158**.

An optically active through-space polymer **158** consisting of planar chiral [2.2]paracyclophane and quaterthiophene units was synthesized via Suzuki cross-coupling method (Scheme 52) [20]. Pure (*R*_P_)-(–)-enantiomer was isolated by chiral HPLC. 

The UV-Vis spectra of (*R*_p_)- and (*S*_p_)-**158** displayed an absorption band with a maximum at 383 nm. The CD spectra of the resulting polymers, (*R*_p_)- and (*S*_p_)-**158** exhibited mirror-image bisignate Cotton effects, with maximum absorption dissymmetry factors *g*_abs_ of (*R*_P_) and (*S*_P_)-polymers **156** at ~410 nm equal to 8 × 10^−4^ and −9 × 10^−4^, respectively. This indicates that in solution, ground-state chirality is strongly induced in the stacked quaterthiophene moieties by the planar chiral [2.2]paracyclophane core. Both (*R*_P_)- and (*S*_P_)-**158** emitted green fluorescence in CHCl_3_ solution with an absolute quantum yield of 7%. While CPL analysis alone makes it challenging to pinpoint the precise excited-state macromolecular conformation (such as zigzag, helical, or parallel geometries), these optically active polymers typically assemble into higher-order chiral superstructures.

#### 3.3.3. Electrochemical Copolymerization or Deposition via Cyclic Voltammetry

**Scheme 53:**
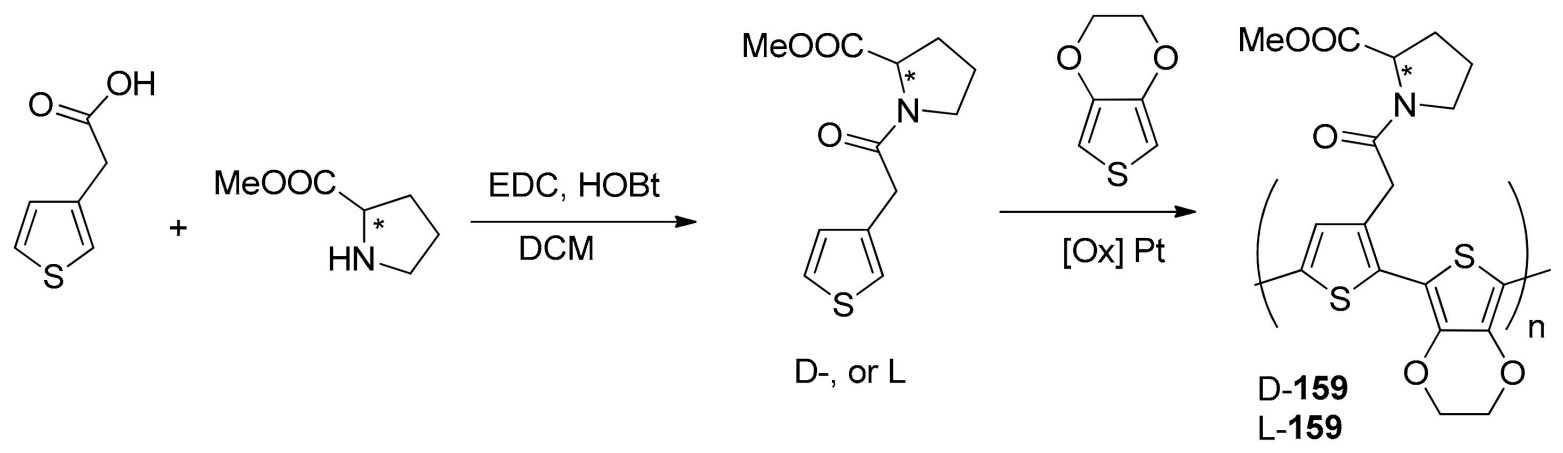
Synthesis of copolymers **159**.

Electrochemical copolymerization using cyclic voltammetry (CV) was employed to synthesize chiral copolymers **159** composed of EDOT and d or l-proline functionalized monothiophene units (Scheme 53) [100]. During the doping/dedoping process, the polymer films underwent reversible electrochromic switching, changing from blue-green in their doped (oxidized) state to blue-brown in their undoped (neutral) state.

**Figure 21:**
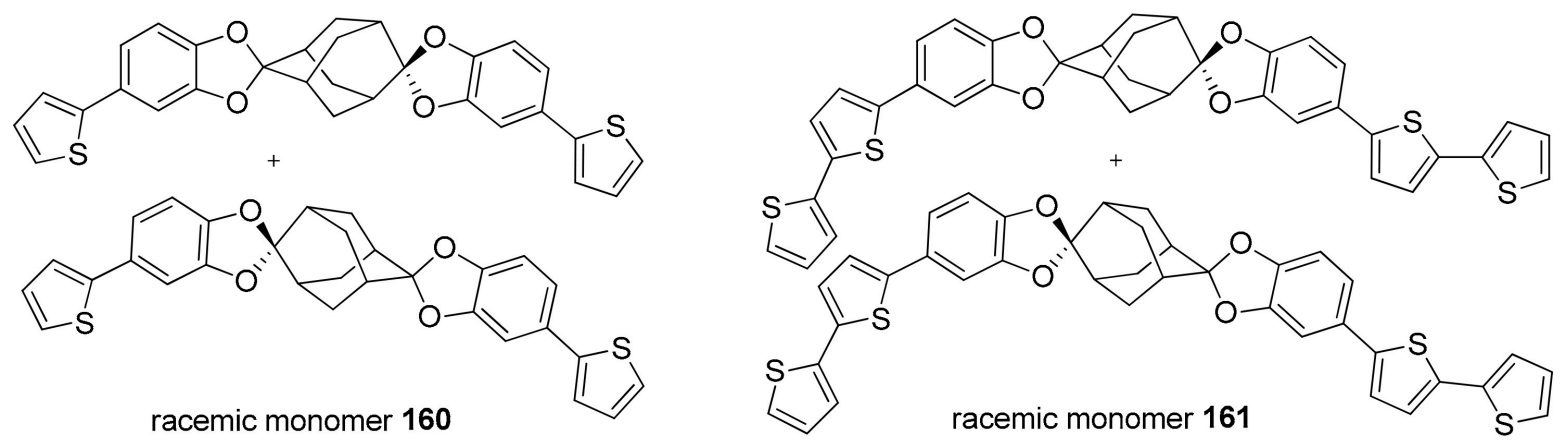
Racemic monomers **160** and **161** for electrochemical polymerization.

Direct electrochemical polymerization via cyclic voltammetry (CV) was used to construct chiral thiophene network from racemic monomers **160** and **161**, which feature an axially chiral adamantane-based linker (Figure 21) [101]. Spectroelectrochemical analysis of films deposited on indium tin oxide (ITO)-coated glass demonstrated strong electrochromism: poly-**161** shifted from yellow-green in the neutral state (<0.4 V) to navy blue when oxidized (1.0 V), whereas poly-**160** transitioned from light yellow to deep purple. Charge carriers (polarons/bipolarons) are localized between adamantyl segments, restricting charge delocalization and favoring an interchain hopping transport mechanism.

### 3.4. Alternative Routes to Chiral Polythiophenes: Chirality Transfer Strategies

Chirality transfer occurs through distinct pathways. One of the alternative strategies operates *in situ* during polymerization, where external chiral components such as chiral solvents [102], chiral polysilanes [103], or metal–organic frameworks (MOFs) [104] serve as an asymmetric templates to enforce specific chiral conformations in the growing polymer. Goto and coworkers have extensively studied in situ chirality transfer, using chiral ionic liquids and liquid-crystalline media [105,106] to induce helical architectures in polythiophenes (PThs); (see Section 3.2.2). Additionally, cellulose nanocrystals have been utilized as liquid-crystalline templates to facilitate chirality transfer in aqueous media [107]. Alternatively, chirality transfer takes place through post-polymerization processes as blending pre-synthesized achiral polythiophenes with chiral templates or complexing them into host –guest/inclusion compounds. Non-covalent intermolecular interactions, including electrostatic binding with biomacromolecules, further drive these post-synthetic transfers. 

A representative example involves the transcription of chirality from a MOF matrix to an achiral polymer backbone [104]. In this approach, chiral transcription from the host framework [La(BTB)]n (**162**) (where BTB = 1,3,5-benzenetrisbenzoate) to unsubstituted polythiophene (PTh) took place inside the MOF nanochannels, and the induced chirality was retained even after liberating the polymer from the host. However, this retention depended on polymer loading: in low-loading composites (**162**:PTh = 1:0.03), host removal resulted in an almost complete loss of optical activity, whereas high-loading (1:0.30) preserved the rod-like morphology of the original channels.

**Figure 22:**
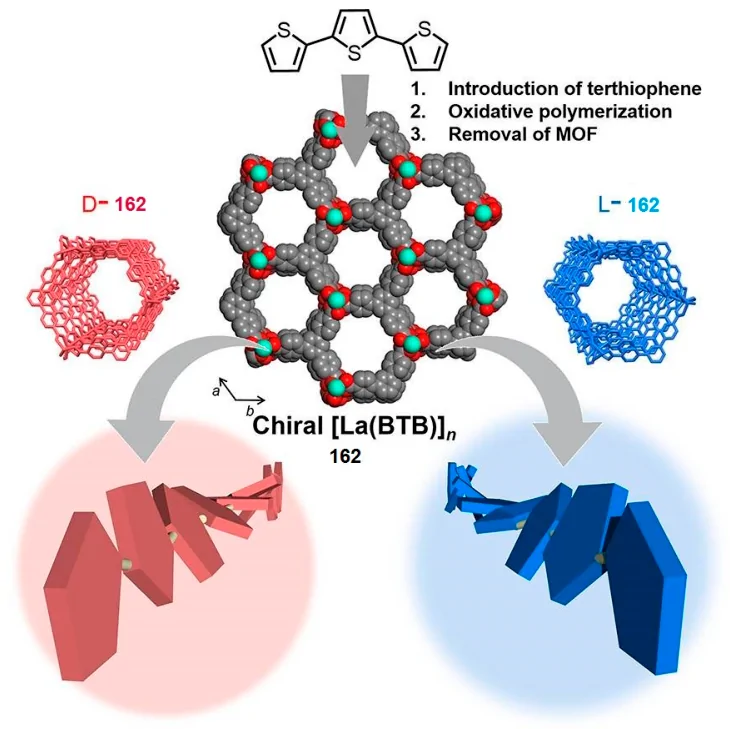
Schematic illustration of *in situ* monomer encapsulation, oxidative polymerization, and host liberation to form chiral polythiophene (PTh) using a chiral MOF matrix. Adopted with permission from Takashi Kitao, Yujiro Nagasaka, Masanobu Karasawa, Toshiki Eguchi, Nobuo Kimizuka, Kazuyuki Ishii, Teppei Yamada, Takashi Uemura; Transcription of Chirality from Metal–Organic Framework to Polythiophene. *Journal American Chemical Society* 2019; 141 (50): 19565–19569. https://doi.org/10.1021/jacs.9b10880 [104]. Copyright © 2019 American Chemical Society.

Synthetically, the process begins by preparing nanochannels of opposite-handed lanthanum-BTB complexes (a (Λ)- or (Δ)-isomers forming three-bladed propeller geometries with left- or right-handed ligand twists). Terthiophene (TTh) monomer was then encapsulated into the pores of host (**162**) via sublimation, followed by *in situ* oxidative polymerization within the channels. The resulting composite displayed strong UV–Vis absorption corresponding to the π → π* transition of the conjugated PTh backbone. Inside the chiral host (**162**), PTh exhibited a Davydov split-type CD signal with vibronic features characteristic of exciton coupling between neighboring chromophores, confirming the helical packing of polymer chains in the pores. In summary, this host–guest transcription strategy offers a convenient route to impart retained chirality to optically inactive polymers (Figure 22).

Chiral poly(3-hexylthiophene) nanowires (chiral nw-P3HT) were developed as organic chiral charge-transfer magnets by inducing supramolecular chirality through a ternary solvent mixture containing a good solvent, a poor solvent, and chiral co-solvent (*R*)-(+)-limonene [89]. The resulting twisted chiral nanowires (nw-P3HTs) exhibited optomagnetic coupling, demonstrated by tuning the polarization of transmitted light using an external magnetic field. Thin films were fabricated by coating the nw-P3HT solution onto quartz substrates, drying under vacuum, and annealing at 100 °C to remove residual (*R*)-(+)-limonene. 

**Figure 23:**
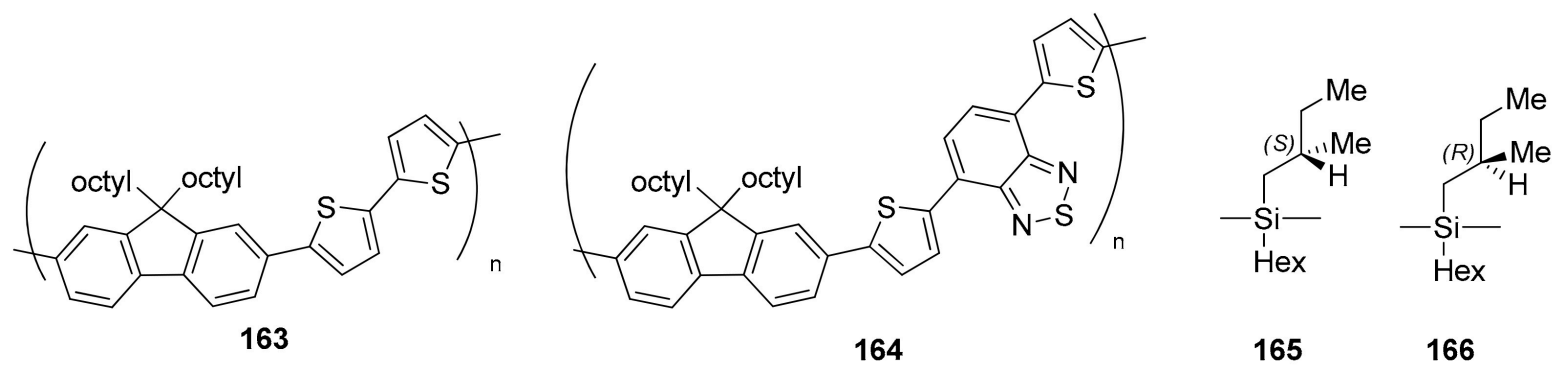
Structure of copolymers: **163**, **164** and chiral templating silanes **165**, **166**.

Similarly, chirality transfer mediated by (*S*)- and (*R*)-limonene enabled the preparation of optically active polymer particles of poly[(9,9-di-*n*-octylfluorenyl-2,7-diyl)-*alt*-bithiophene] from the corresponding achiral copolymer precursor **163** (Figure 23) [102].

Optimal chirality induction was achieved using a ternary solvent mixture of (*S*) *or* (*R*) limonene–chloroform–methanol in a ratio of 2:0.3:0.7 (*v*/*v*/*v*). Polymer particles of **163** exhibited the highest CD and CPL signal intensities. Both the choice of alcohol (acting as the poor solvent) and limonene enantiopurity strongly influenced the magnitude and sign of the CD and CPL signals, for instance, replacing methanol with ethanol inverted the sign of the CD spectrum, signaling a reversal in helical handedness. However, this limonene-mediated chirality transfer approach was restricted to poly(fluorene-thiophene) particles and cannot be applied to all π-conjugated polymers.

The copolymer poly{[9,9-di-*n*-octyl-fluorene]-*alt*-[4,7-bis(thiophen-2-yl)-2,1,3-benzothiadiazole]} (**164**) was complexed with chiral poly[*n*-hexyl-(*S*)-2-methylbutylsilane] (**165**) or poly[*n*-hexyl-(*R*)-2-methyl-butylsilane] (**166**) (Figure 23) to induce the formation of CD- and CPL-active heteroaggregates under self-assembly conditions [103]. The induced optical activity was fully maintained following photolytic scissoring of the polysilanes via UV irradiation at 313 nm. This provides a clear example of chirality transfer from sacrificial polysilane templates to conjugated copolymer chains that become intrinsically chiral upon aggregation. 

**Scheme 54:**
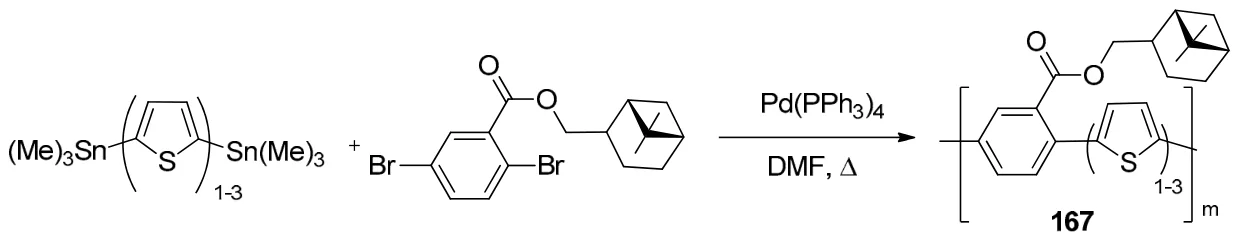
Synthesis of copolymers **167**.

Copolymerizing a non-thiophene monomer bearing a chiral substituent with achiral thiophene units is an effective strategy for imparting chirality to thiophene-based copolymers. For example, Stille cross-coupling between achiral thiophene units (containing 1–3 thiophene rings) and benzene-derived monomers with chiral side chains produced a series of optically active copolymers **167** (Scheme 54) [108]. These point-chiral substituents induce helical supramolecular organization along the conjugated copolymer backbone. 

**Scheme 55:**
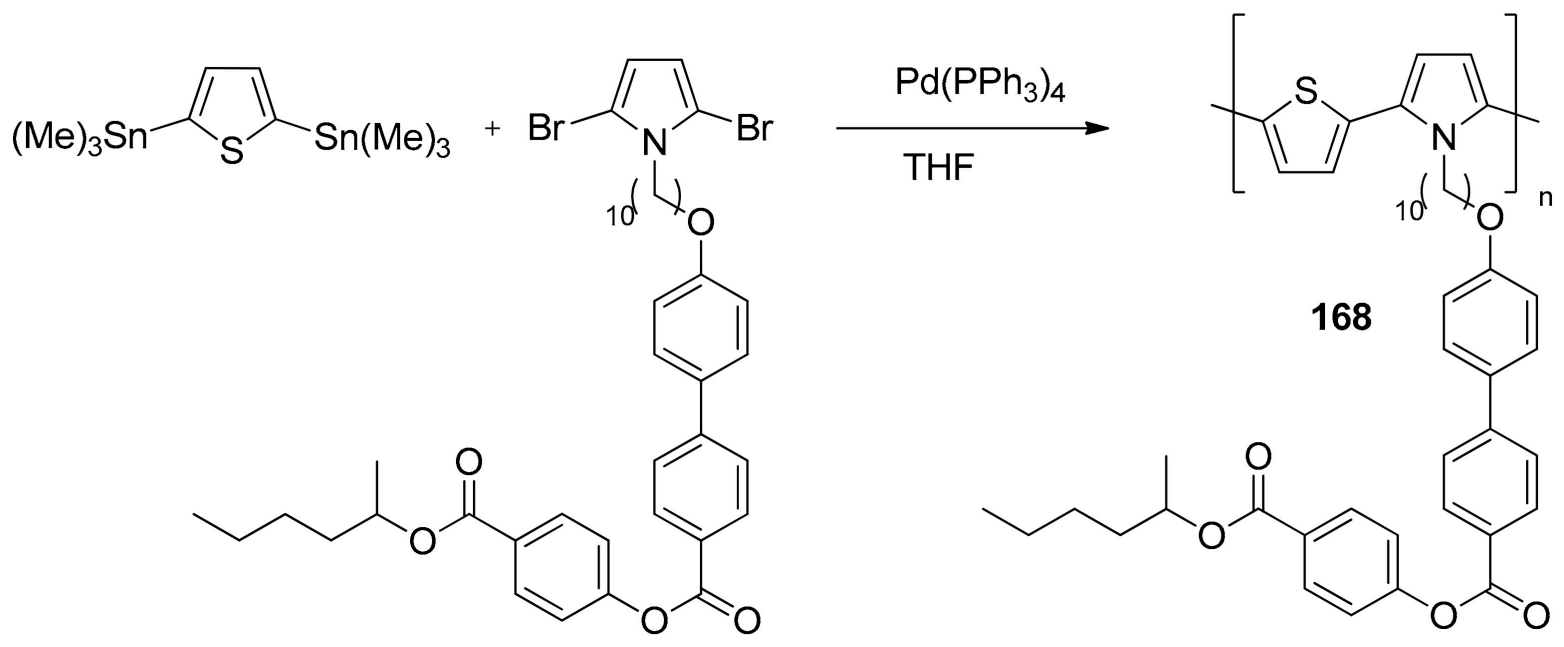
Synthesis of copolymer **168** made up of unsubstituted thiophene units and pyrrole units with mesogenic group in *N* position.

In a follow-up study, the same research group synthesized copolymer **168** comprising unsubstituted thiophene units and *N*-substituted pyrrole units bearing mesogenic groups [109] via Stille polycondensation (Scheme 55). The resulting copolymer exhibited lyotropic liquid-crystalline behavior. Notably, these findings demonstrated that chiral mesogenic side chains can induce helicity along the π-conjugated backbone even across extended distances.

**Scheme 56:**
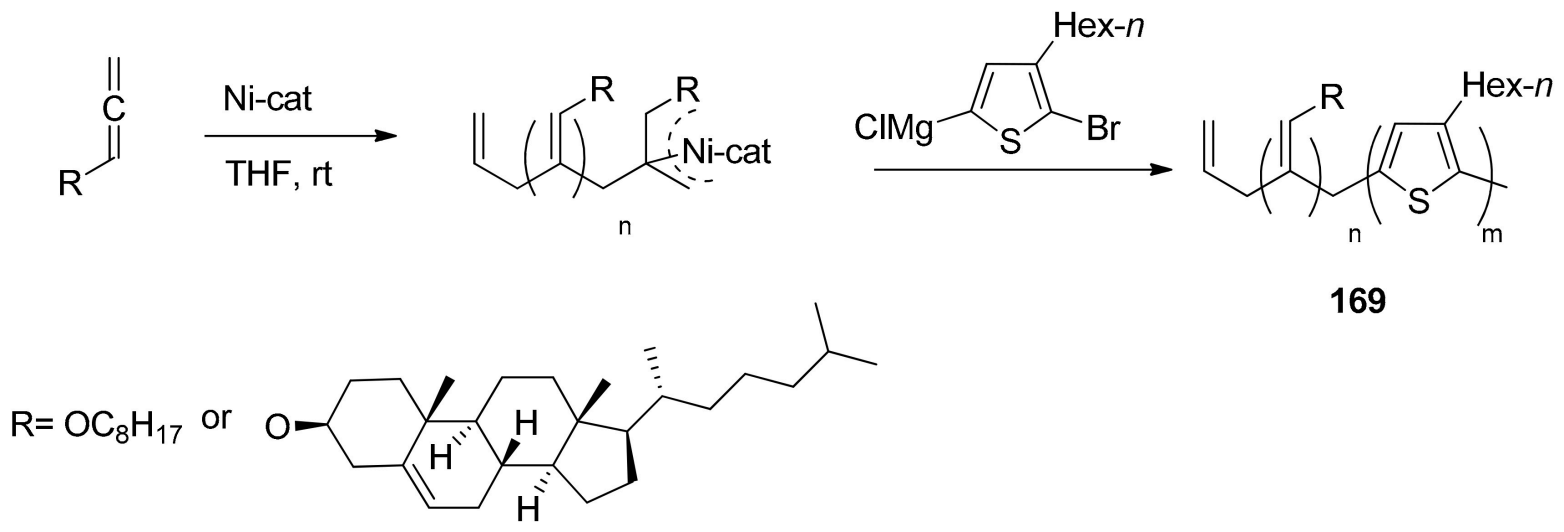
Synthesis of single-handed supramolecular block copolymers **169**.

Recently, the controlled synthesis of single-handed supramolecular block copolymers **169** from achiral poly(3-hexylthiophene) (P3HT) derivatives was achieved via asymmetric living supramolecular polymerization [110]. This supramolecular assembly was initiated using right-handed helical seed micelles. The constituent block copolymers comprising either chiral helical polyallene or achiral polyallene paired with P3HT were synthesized via a Ni^II^ catalyzed, sequential one-pot block copolymerization of the corresponding monomers (Scheme 56). The right-handed supramolecular block copolymers showed white-light emission and circularly polarized luminescence.

## 4. Applications 

### 4.1. Redox Switching in Chiral Polythiophenes

Supramolecular or macromolecular chirality of the investigated polymers can be switched (‘on’ and ‘off’) via electron transfer: oxidation of the polythiophene main chain achieved using chemical oxidants (e.g., copper(II) salts) or an anodic electrochemical potential and can be reversed through reduction using chemical reducing agents (such as amines or hydrazine) or electrochemical dedoping.

Redox-switchable supramolecular chirality was first demonstrated in HT-regioregular poly[3-{4-((*R*)-4-ethyl-2-oxazolin-2-yl)phenyl}thiophene] [111]. Reversible control over its chiral assemblies was achieved via main-chain electron transfer: oxidative doping using copper(II) trifluoromethanesulfonate [Cu(OTf)_2_] altered the assembly, while dedoping with triethylenetetramine restored the induced circular dichroism (CD) signals. Since this initial study, numerous examples of reversible chirality switching in supramolecular aggregates via electron transfer have been reported. 

**Figure 24:**
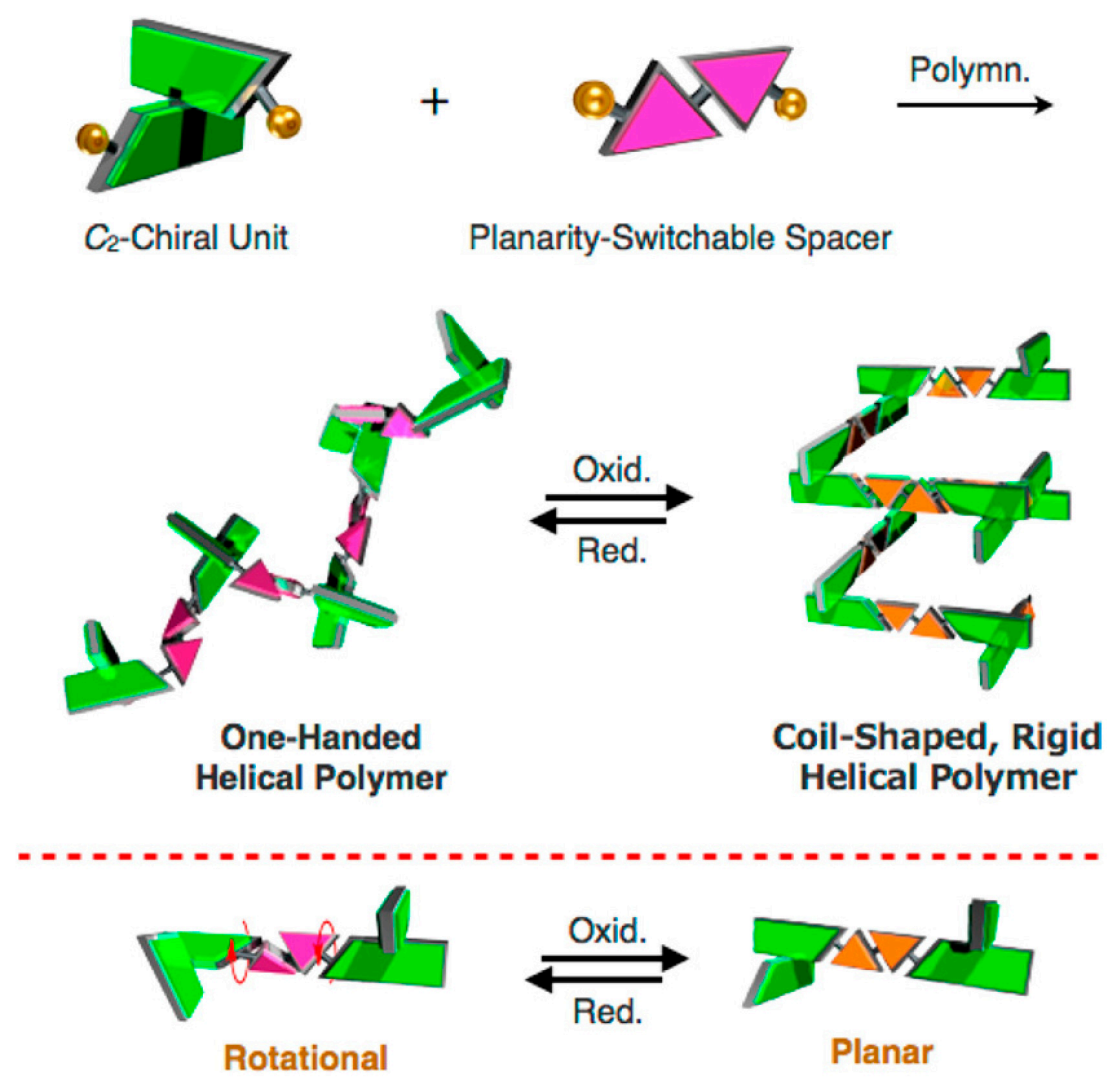
Schematic illustration of the folding system with the planarity switchable spacer. Reprinted with permission from: Hitoshi Okuda, Yasuhito Koyama, Satoshi Uchida, Tsuyoshi Michinobu, Hiromitsu Sogawa, Toshikazu Takata; Reversible Transformation of a One-Handed Helical Foldamer Utilizing a Planarity-Switchable Spacer and *C*_2_-Chiral Spirobifluorene Units. *ACS Macro Lett.* 2015; 4 (4): 462–466. https://doi.org/10.1021/acsmacrolett.5b00176 [94]. Copyright © American Chemical Society.

Redox-responsive, one-handed helical foldamers (Figure 24) consisting of both quaterthiophene and *C*_2_-chiral spirobifluorene skeletons provide a platform for chiral materials with dynamically controllable higher-order structures [94]. The concept of a redox-driven folding system is based on changes in main-chain rigidity. Takata et al. proposed that these materials have potential applications as hosts, catalysts, or highly selective sensors. 

### 4.2. Chiral Sensors

Chiral polymer-based electrodes are essential in biochemistry and pharmacology [7]. Their ability to differentiate biologically active enantiomers arises from the presence of stereocenters incorporated into the side chains of polymers depositing electrodes. By employing electrochemical methods i.e., CV (cyclic voltammetry) or DPV (differential pulse voltammetry), it is possible to differentiate for instance amino acid enantiomers [112]. In addition to polythiophene films bearing intrinsic stereocenters within their side-chain architectures, chiral electroactive surfaces on polymer-modified electrodes can be generated by doping achiral polythiophenes with chiral counteranions.

**Figure 25:**
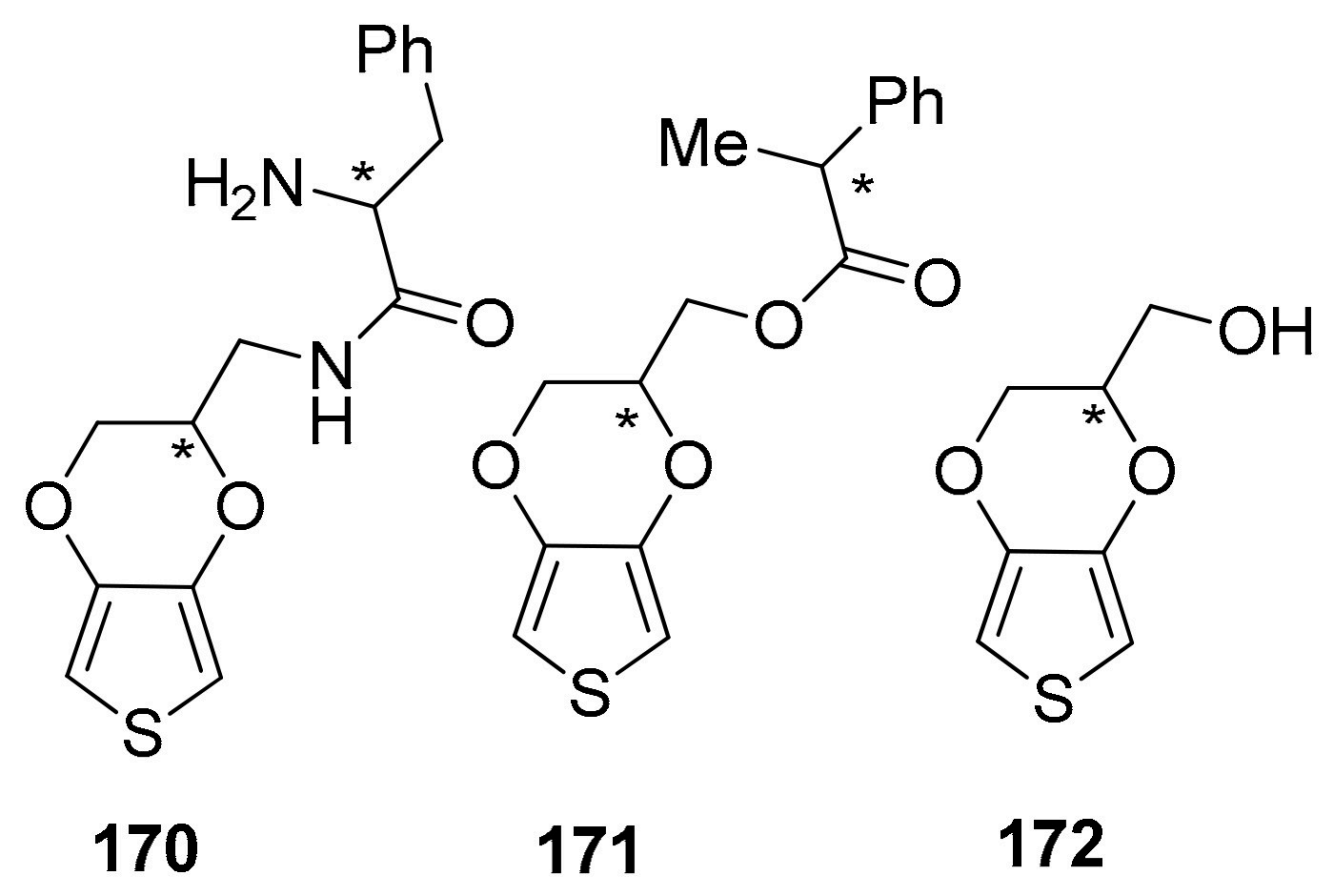
Structure of **170**–**172**.

Electropolymerization was employed to prepare chiral polythiophene-modified electrodes from i.e., terthiophenes functionalized with d-alanine or d-leucine [66,67]. l-Leu-functionalized poly(EDOT) was electrodeposited onto electrodes in a manner similar to the coating of platinum (Pt) electrodes with d-leucine-substituted polyterthiophenes [113]. The polymer main chains were found to adopt helically twisted structures upon the introduction of chiral amino acid side chains, with dedoping serving to enhance the overall chirality. Conversely, PF_6_^−^ dopant intercalation between the polymer chains expanded interchain spacing, relaxing helical twisting and weakening chirality. Chiral voltammetric biosensors based on functionalized poly(EDOT)s were fabricated for the detection of phenylalanine and 3,4-dihydroxyphenylalanine (DOPA) by electrodepositing polymers derived from monomers **170** and **171** onto a glassy carbon electrode (Figure 25).

Enantiodifferentation of chiral compounds was successfully demonstrated using a glassy carbon electrode (GCE) coated with electropolymerized poly(EDOT) derived from monomer **172**. This modified electrode registered electrochemical responses capable of distinguishing between the enantiomers d-/L-DOPA, d-/L-tryptophan, and (*R*)-/(*S*)-propranolol [114]. The pathway for enantiomeric differentiation involves the formation of two intermolecular hydrogen bonds between DOPA and hydroxymethylated polyEDOT molecules. 

**Figure 26:**
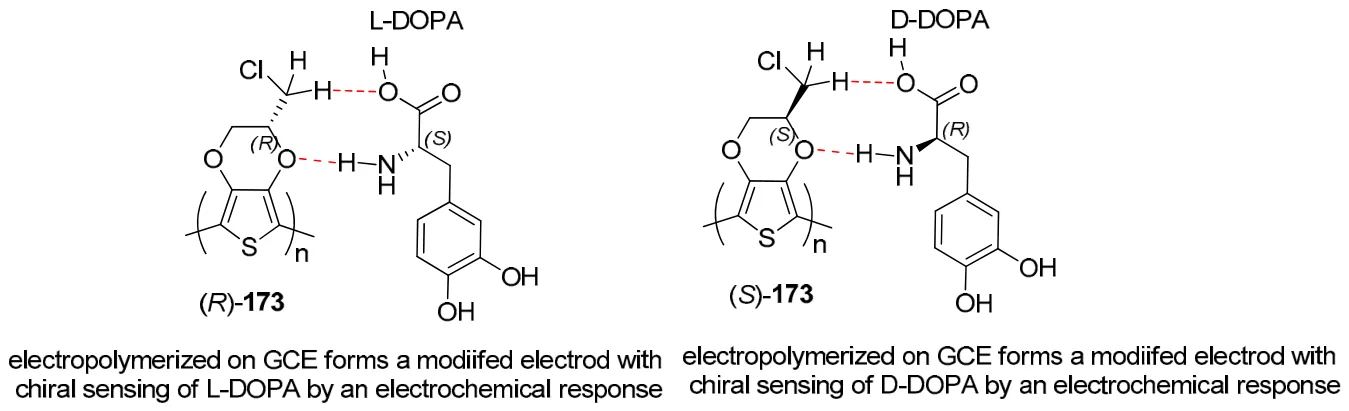
The interaction between DOPA enantiomers and chiral poly((*S*)-, (*R*)-2-(chloromethyl)-2,3-dihydrothieno [3,4-*b*][1,4]dioxine) [(*S*)-, (*R*)-**173**].

When DOPA enantiomers interact with the surface of the modified electrode, l-DOPA more easily binds to the right-handed derivative (*R*)-**173** on a glassy carbon electrode, whereas the binding of d-DOPA is unfavorable. This selective interaction stems from the spatial conformation of L-DOPA which forms a thermodynamically favorable complex with the (*R*)-**173** polymer film, whereas d-DOPA shows a preference for the (*S*)-**173** enantiomer (Figure 26) [115].

Polythiophene acetate functionalized with an enzyme rich with histidine, serine and glutamic acid units was deposited on a glassy carbon electrode (GCE) to obtain a chiral biosensor able to detect the organophosphorus pesticides. Catalytic hydrolysis of the organophosphorus pesticides led to *p*-nitrophenol, which was further oxidized to form nitroquinone. The electron and proton transfers in the conversion of hydroquinone to quinone appearing at modified electrode surface were recorded in the square wave voltammetry (SWV) [116].

**Figure 27:**
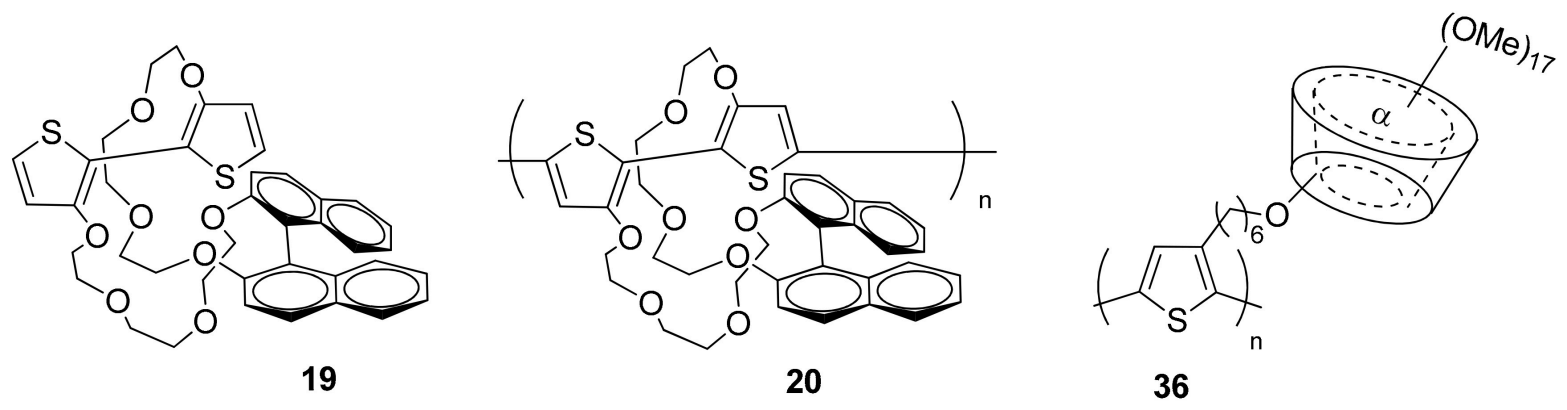
Representative chiral and achiral (poly)thiophene derivatives in host-guest type interactions.

The chiral discrimination capability of the binaphthocrown ether functionalized polythiophene **20** (Figure 27) was evaluated against the enantiomeric methyl esters of alanine (Ala-OMe⋅HCl), valine (Val-OMe⋅HCl), leucine (Leu-OMe⋅HCl), proline (Pro-OMe⋅HCl), and phenylalanine (Phe-OMe⋅HCl), which served as cationic chiral guests [40]. Complexation of the monomeric unit **19** with either l- or d-Ala-OMe⋅HCl induced planarization of the binaphthyl moiety upon guest inclusion, demonstrating that the monomer itself functions as an effective chirality-sensing host. In contrast, adding d-Ala-OMe⋅HCl to the polythiophene solution yielded distinct spectral modifications. The chiroptical changes (a hyperchromic effect in the UV-VIS spectra and a hypsochromic shift of the circular dichroism maximum) indicated that the π-conjugated polymer backbone underwent a macrostructural twisting upon guest inclusion.

Peptide chirality sensing by polythiophene containing a cyclodextrin pendant **36** (Figure 27) was evaluated in 50% aqueous methanol toward a series of amino acid derivatives, including phenylalanine methyl ester (Phe-OMe), tryptophan methyl ester (Trp-OMe), histidine (His), tyrosine (Tyr), and 4-nitrophenylalanine (4-NO_2_-Phe) as well as the dipeptides Phe-Phe, Phe-Trp, Gly-Tyr, and Tyr-Tyr. The mechanism underlying this chiroptical sensing relies on the enantiodifferentiating complexation of the guests by the pendant cyclodextrin moieties, which directly modulates the conformation of the polythiophene backbone and induces distinct UV–Vis spectral shifts [17].

**Figure 28:**
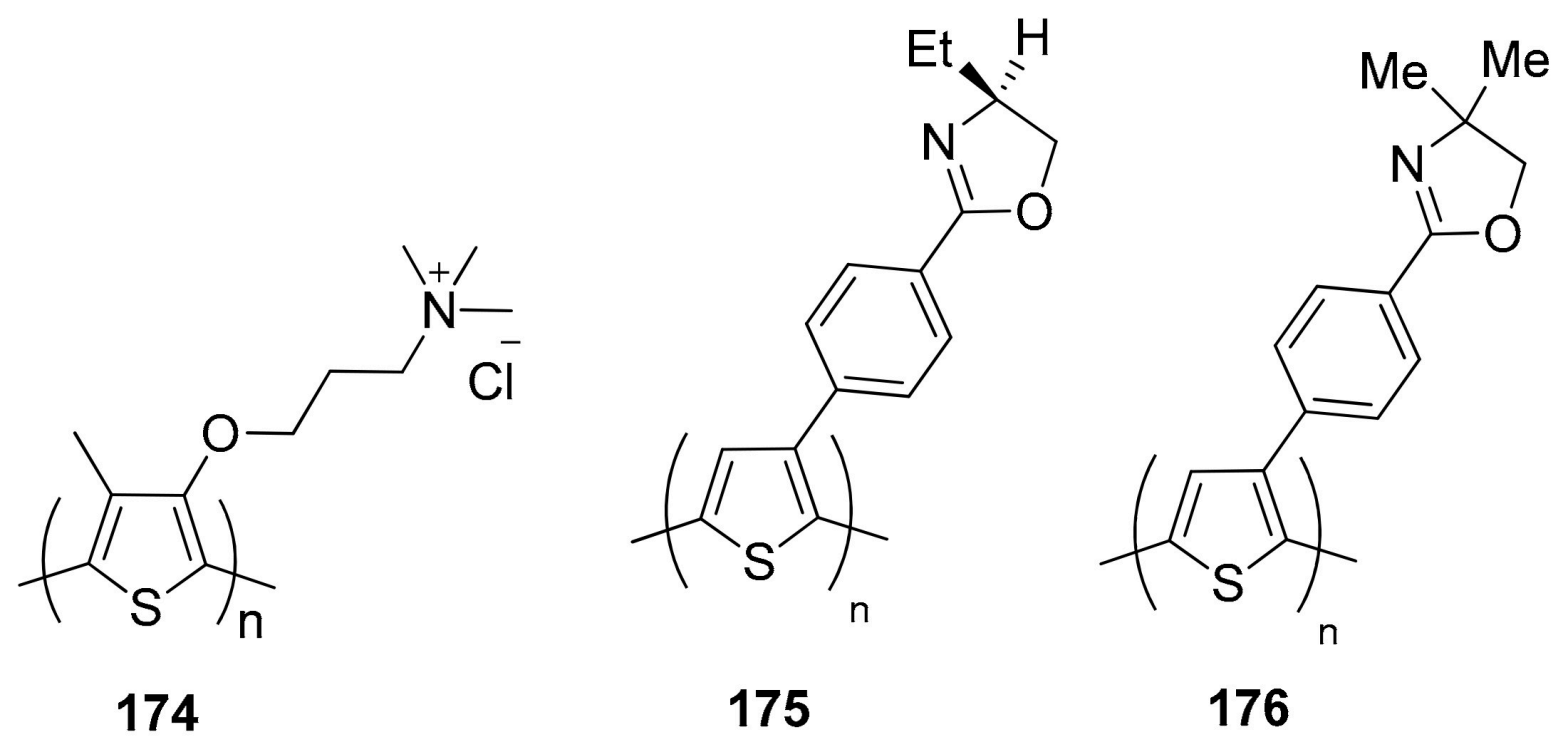
Representative chiral and achiral polythiophene derivatives in molecular recognition.

Aggregates of achiral polythiophene **174** (Figure 28) showed induced CD (ICD) intensities when exposed to galactose, glucose, and fructose enantiomers in chloroform-rich media [117]. The behavior of molecular recognition (arising from non-covalent interactions) shows the potential of such supramolecular systems for detecting sugar structures.

The chiral, regioregular (head-to-tail) (poly [3-{4-((*R*)-4-ethyl-2-oxazolin-2-yl)phenyl}thiophene]) (**175**; Figure 28) self-assembled into supramolecular chiral aggregates in poor solvents^9^ or in the presence of metal salts capable of coordinating to the oxazoline residues even in CHCl_3_. The CD studies showed a characteristic ICD in the π → π* transition region which confirmed the formation of a supramolecular chirality of the aggregates of **175**. Furthermore, this chiroptical response could be reversibly controlled by the addition or removal of electrons from the polymer main chain (via redox reversible switching) [34].

The achiral polythiophene with an oxazoline residue, (poly [3-{4-(4,4-dimethyl-2-oxazolin-2-yl)phenyl}thiophene]) **176** (Figure 28) aggregated in the presence of metal salts, such as AgOTf. This process induces unique supramolecular chirality when complexed with chiral amines or in the presence of **175**, resulting in observable induced circular dichroism. A plausible four-step mechanism for the chiral supramolecular aggregation of **176** in the presence of Ag(I) and chiral amines begins with the molecule adopting a helically twisted conformation, either s-*cis* or s-*trans*, from an initially random-coil state in a good solvent. The next step involves complexation with Ag(I) ions, resulting in an equal mixture of right- and left-handed helical aggregates. Further addition of chiral amines promotes the formation of predominately right- or left-handed helical aggregates.

Furthermore, supramolecular chirality is also transferred from chiral **175** to achiral **176** chains when metal salts coordinate to the oxazoline function. These supramolecular assemblies could serve as chiral materials for chiral recognition or may be employed as supramolecular chiral catalysts for asymmetric synthesis [34].

**Figure 29:**
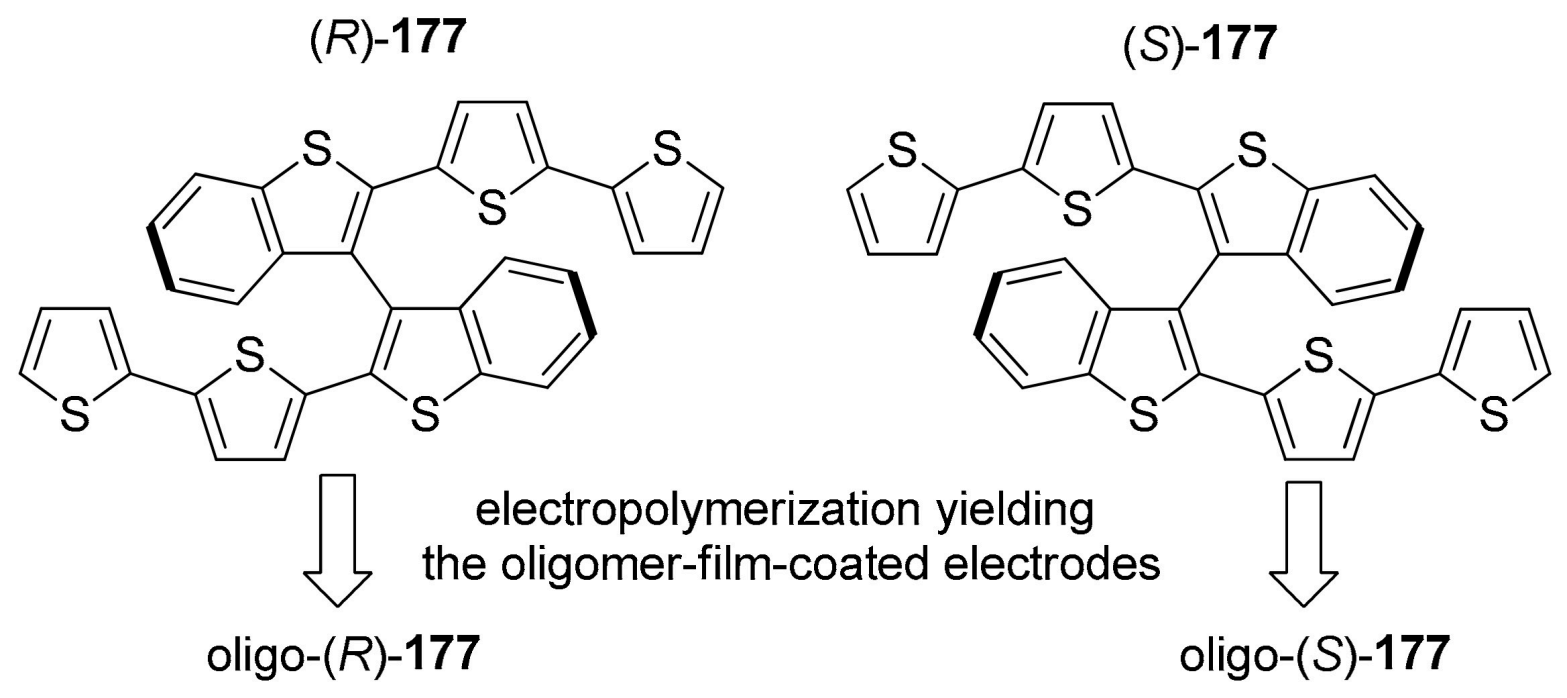
Chemical structures of (*R*)-2,2-bis-(2,2-bithiophene-5-yl)-3,3-bithianaphthene [(*R*)-**177**] (**left**) and (*S*)-2,2-bis-(2,2-bithiophene-5-yl)-3,3-bithianaphthene [(*S*)-**177**] (**right**) as monomers for electrodeposited heterocycles.

Sannicolò et al. [118] reported inherently chiral electroactive conjugated thiophene heterocycles (oligo-**177**) composed of 12 and 18 fully conjugated thiophene units, derived from the enantiopure building blocks (*S*)- and (*R*)-**177** (Figure 29). These thiophene-based materials combine reversible potential-controlled chiroptical switching with electrochemical enantiodiscrimination. 

**Figure 30:**
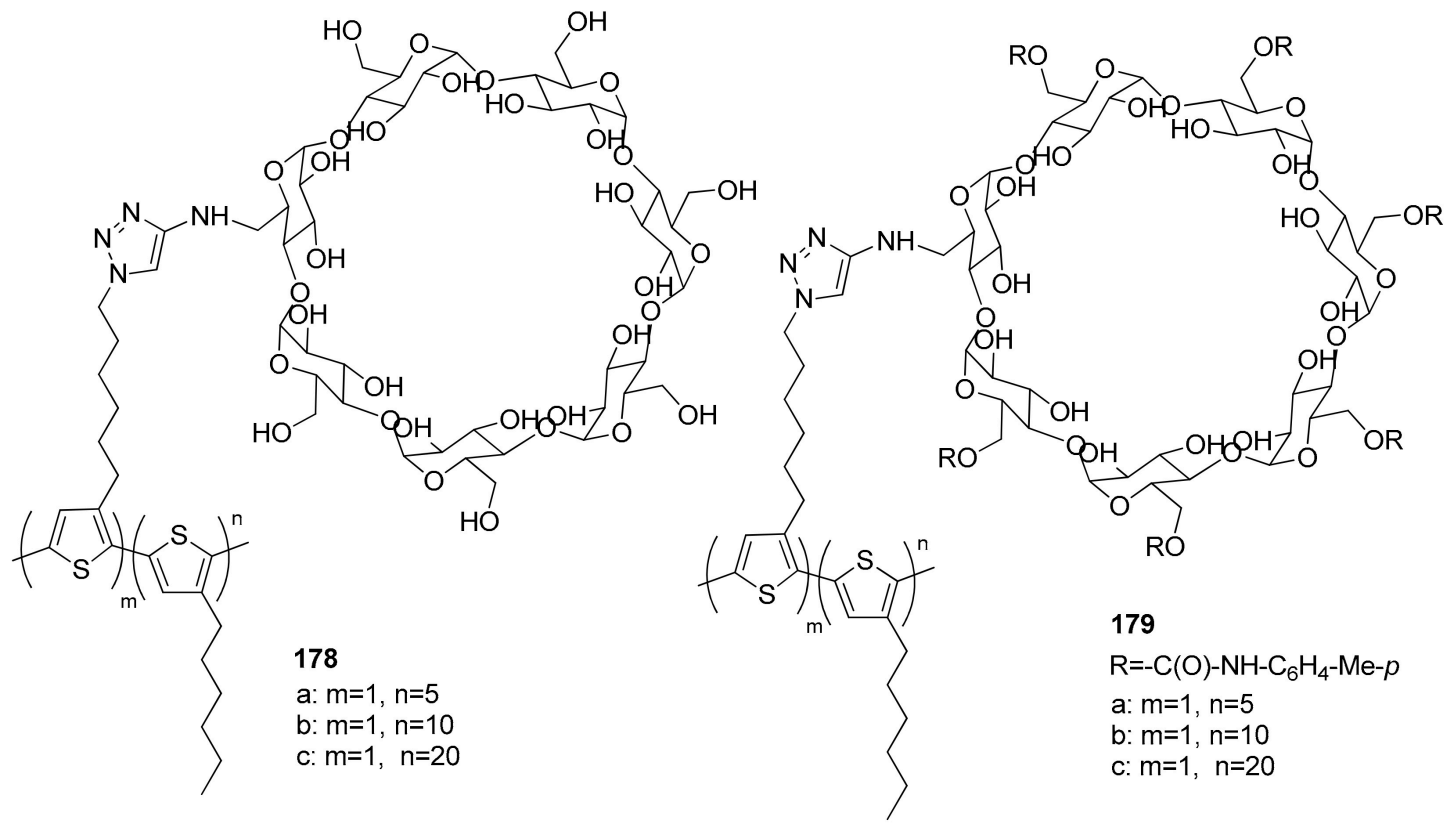
Thiophene-based copolymers **178** and **179**.

Thiophene-based copolymers containing a cyclodextrin pendant were found to serve as electrochemical sensors for the enantiodifferentiation of amino acid enantiomers [119]. In the design of block copolymers **178** and **179** (Figure 30), high conductivity was achieved through the polythiophene backbone, while stereoselective interactions with enantiomers (the chiral analytes) were enabled by the cyclodextrin pendant groups. Differential pulse voltammetry (DPV) was applied to study electrochemical sensing performance of the copolymer/GCE electrode systems.

**Figure 31:**
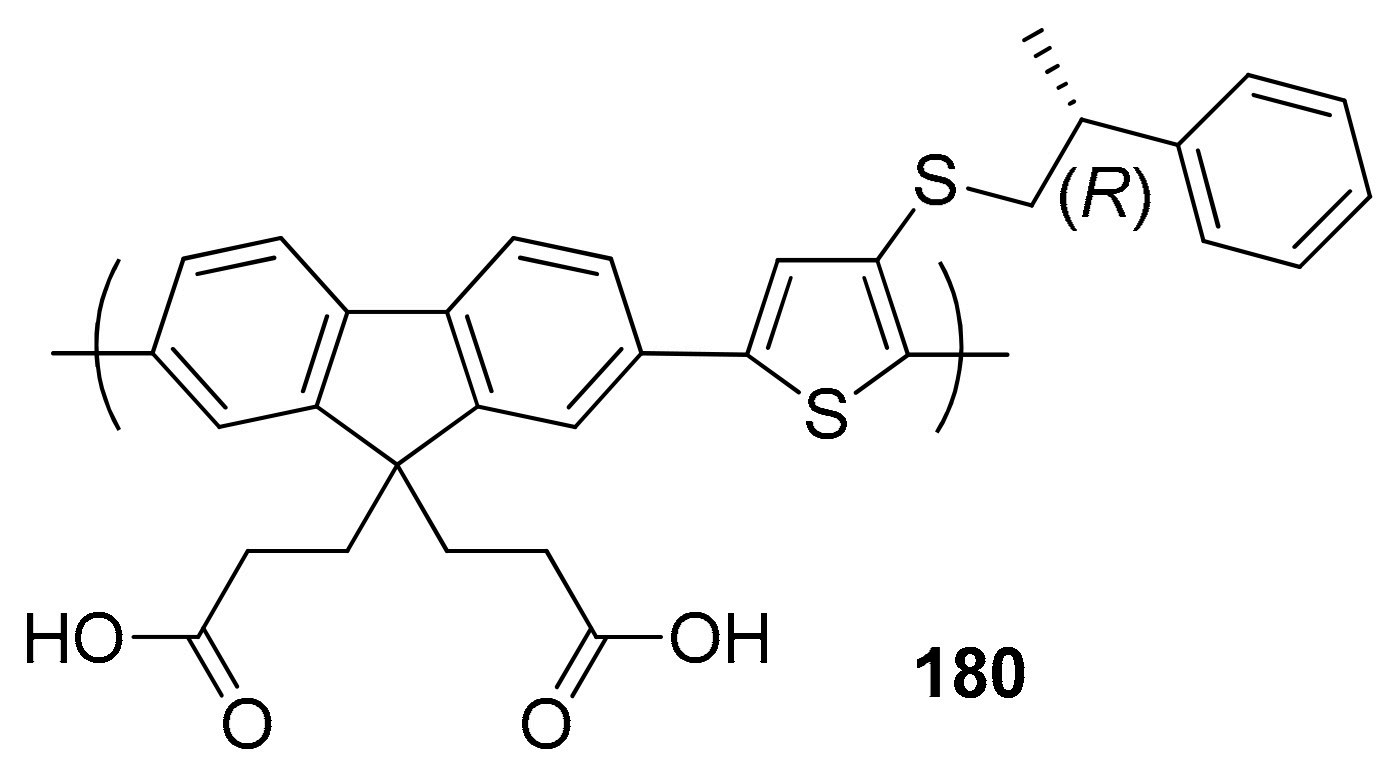
The polymers applied in the electrode construction for hydrogen production.

Chiral poly(fluorene-*co*-thiophene) **180** (Figure 31) was employed as a conductive coating for TiO_2_ photoanodes in photoelectrochemical water splitting [120]. Compared with the corresponding achiral analogue, the chiral polymer reduced the onset potential by approximately 0.2 V while increasing both the photocurrent density and the hydrogen evolution rate by about fourfold. The photoanode of integrated polymer **180** with CdSe quantum dots initiated hydrogen evolution at 0.1 V, whereas the corresponding achiral system required 0.4 V. These results demonstrate the potential of chiral polythiophene-based conductive polymers as functional materials for photoelectrochemical hydrogen production. 

Doping nonchiral poly(3-hexylthiophene) with different triflate salts creates chemical-specific conductimetric responses to target gases [121]. The interaction between the analyte gas and the triflate acceptor produces distinct conductance modulation patterns, allowing the same polymer, when doped with at least two different triflates, to discriminate between water, acetone, and ethanol under ambient conditions. 

### 4.3. Chiral Catalysis

**Figure 32:**
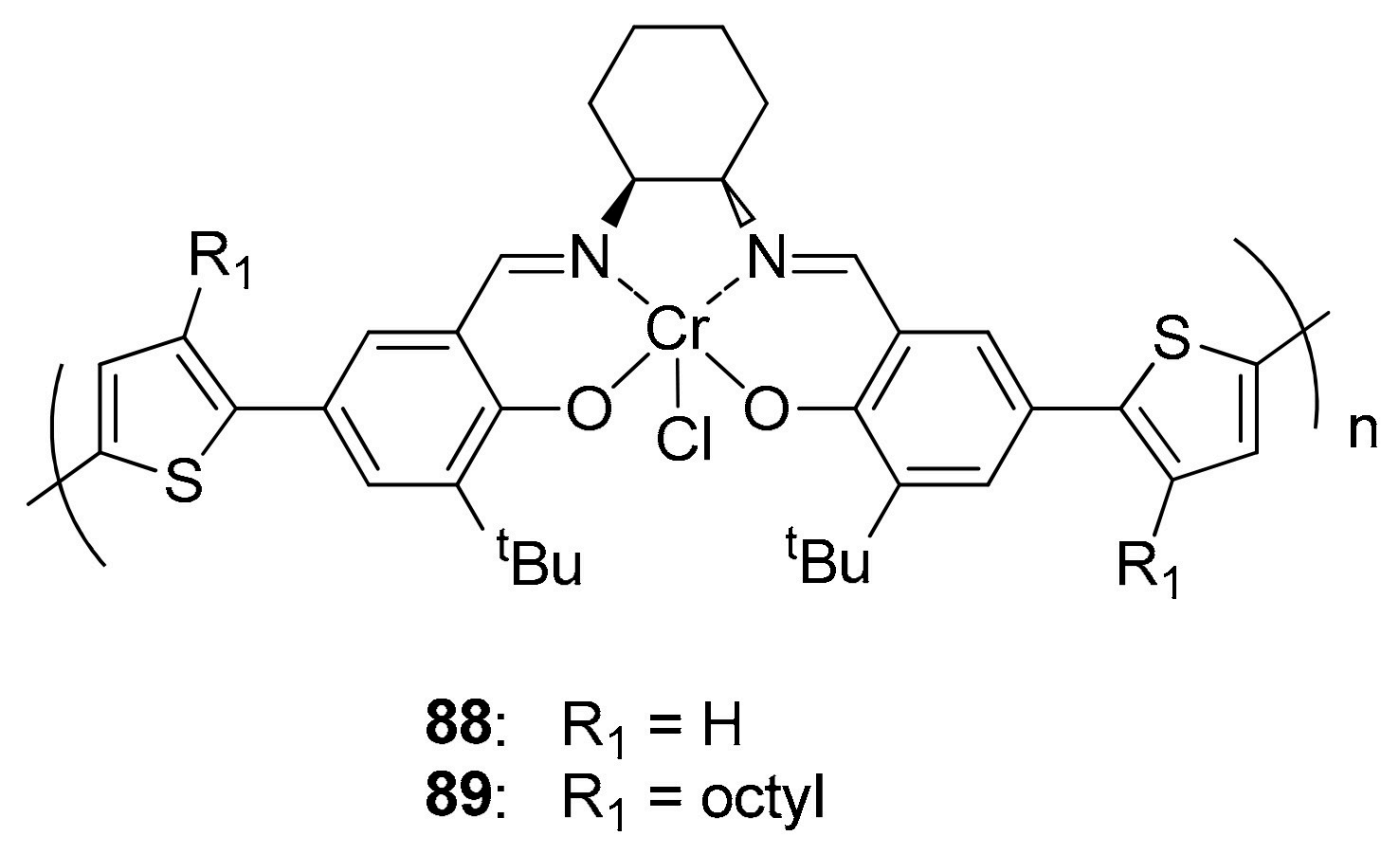
Structure of chromium salen polymers.

Electropolymerized chromium salen polymers serve as stable, recyclable heterogeneous catalysts for asymmetric transformations, including hetero-Diels–Alder and Henry reactions [68] (already mentioned in Section 3.2.1 (Figure 32). Polymer **88** is the first active heterogenized chiral salen catalyst reported for asymmetric Henry reactions, with both polymer variants **88** and **89** offering excellent catalytic stability upon reuse.

### 4.4. Magneto-Optical (MO), Electromagnetic Materials and Layers

**Figure 33:**
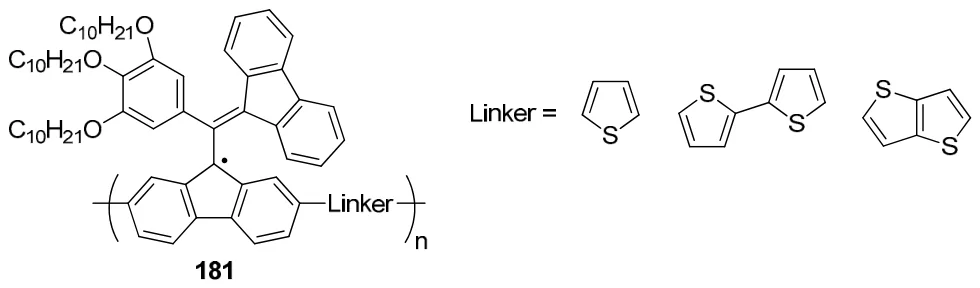
Structure of radical polythiophenes **181**.

Polythiophene-based materials have also attracted significant attention for magneto-optical and electromagnetic applications. Specifically, conjugated radical polythiophenes bearing 1,3-bisdiphenylene-2-phenylallyl (BDPA) **181** (Figure 33) units exhibit pronounced magneto-optical activity, with absolute Verdet constants reaching up to (2.80 ± 0.84) × 10^4^ deg T^−1^ m^−1^ at 532 nm, a performance comparable to commercial inorganic materials. Moreover, the incorporation of radical moieties enables modulation of the Faraday rotation sign through the polymer’s spin state, highlighting their potency for magnetic and photonic devices [122].

**Figure 34:**
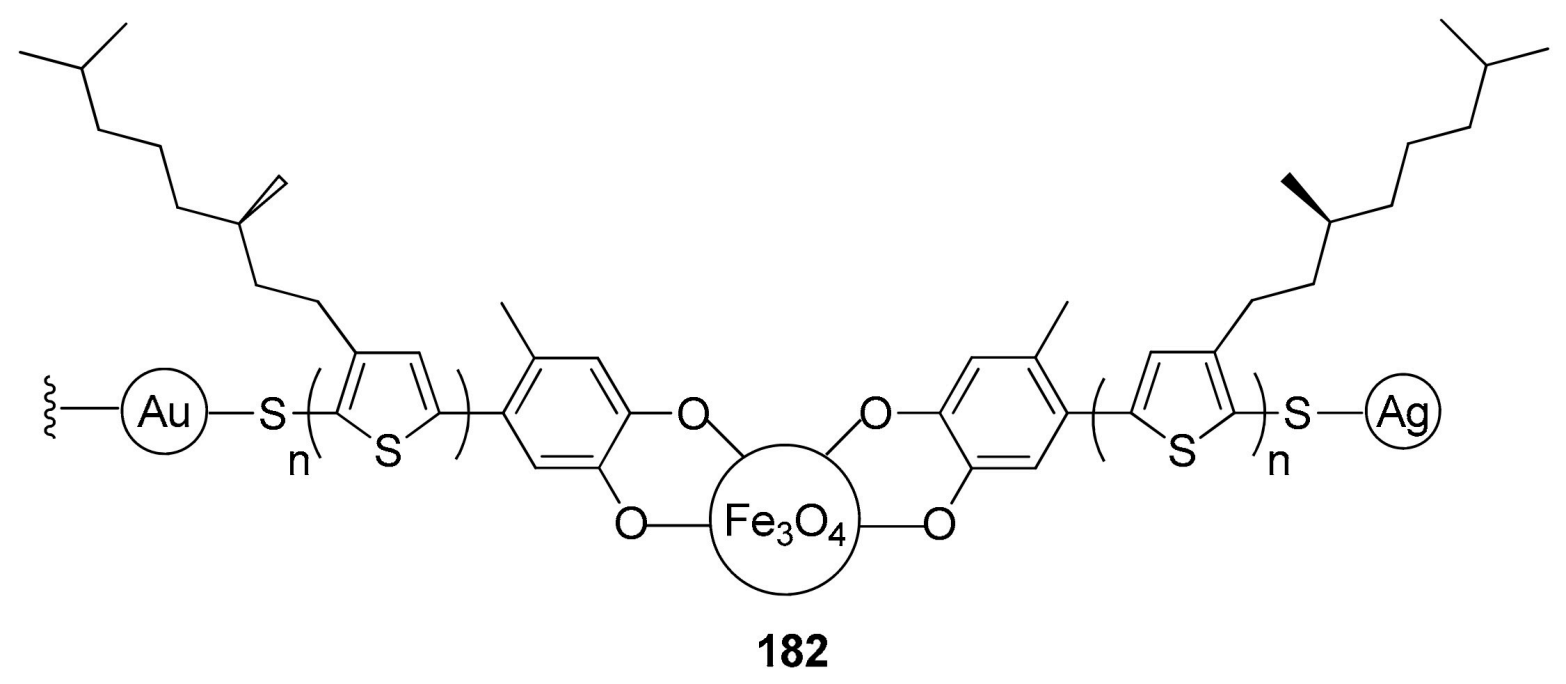
Nanoparticle assembly featuring gold, silver, and magnetite nanoparticles connected via α,ω-bifunctionalized poly(3-((*S*)-3,7-dimethyloctyl)thiophene) bearing catechol and thiol end groups.

In a subsequent study, α,ω-bifunctionalized chiral polythiophenes were used as linkers to fabricate conjugated polymer/nanoparticle hybrid assemblies with alternating magnetite and metallic layers **182** (Figure 34). The failure to detect a strong nonreciprocal optical response was ascribed by Koeckelberghs et al. to high polymer polydispersity and poor intramolecular stacking, which prevented the enhancement of electric quadrupole interactions [86].

### 4.5. Bioactivity Evaluation

**Figure 35:**
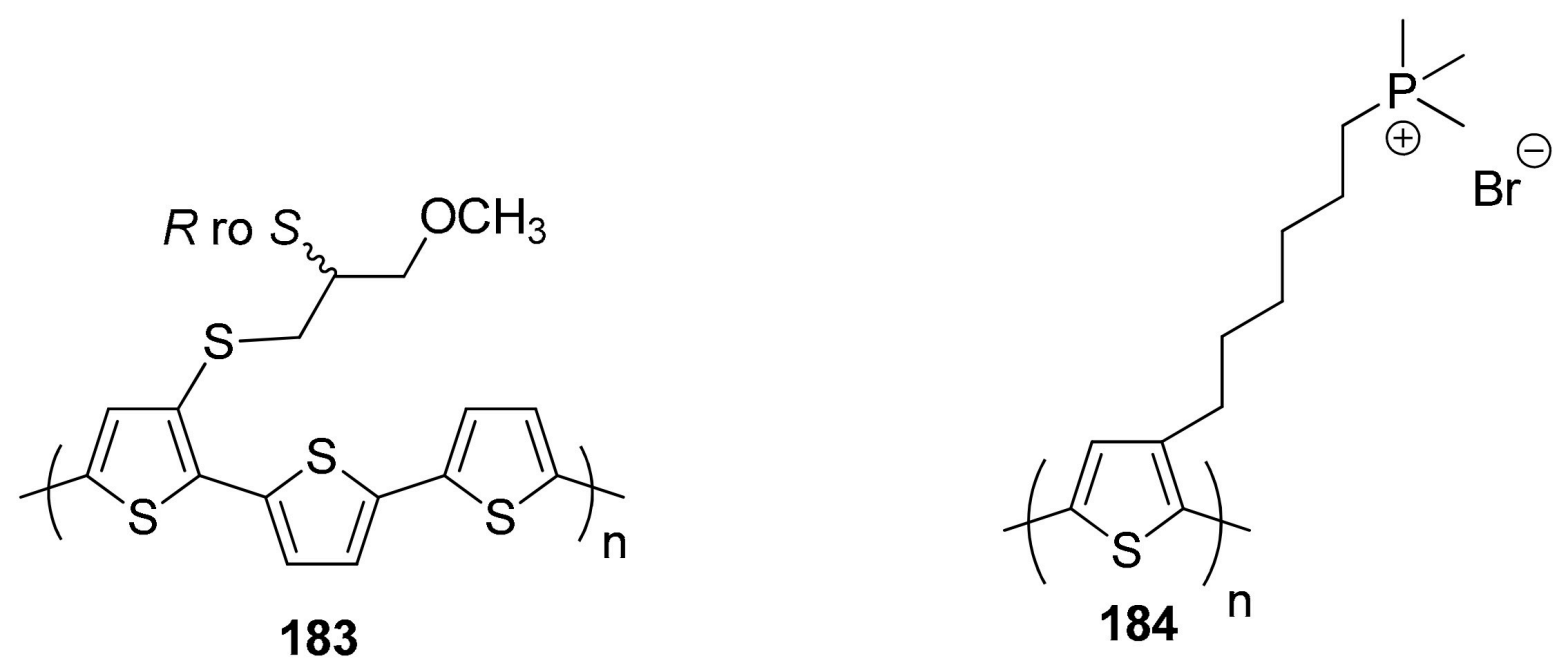
Enantiopure *R*- and *S*-polythiophene conjugate **183** and cationic 3-substituted polythiophene derivative **184**.

Nanoparticles derived from enantiopure (*R*)- and (*S*)-polythiophene **183** [(*R*)*-,* (*S*)-NPs] (Figure 35) showed excellent cytocompatibility toward mouse NIH-3T3 fibroblasts, maintaining cell viability above 70% after 24–72 h alongside distinct enantioselective behavior. Chirality-dependent responses were also observed in antibacterial assays [48]. (*R*)-NPs adsorbed larger amounts of serum proteins and showed greater activity against Gram-negative *Escherichia coli*. In contrast, (*S*)-NPs displayed 3–10% higher cellular uptake, stronger organelle co-localization, and higher efficacy against Gram-positive *Staphylococcus aureus*. Overall, the intrinsic chirality of polythiophene nanoparticles governs protein adsorption, intracellular localization, and antimicrobial activity, highlighting the potential of chiral conjugated polymeric nanoparticles as tunable platforms for bioimaging, antimicrobial materials, and targeted drug delivery.

The cationic polythiophene poly [3-(6′-(trimethylphosphonium)hexyl)thiophene-2,5-diyl] (**184**) (Figure 35) enabled continuous monitoring of *Hpa*I endonuclease activity through induced circular dichroism (ICD), without requiring covalent labeling of the DNA substrate [123]. Enzymatic cleavage was detected as a progressive decrease and blue shift of the ICD signal, with the major spectral changes occurring within 70–100 min, in good agreement with PAGE analysis showing ~30% DNA cleavage after ~90 min. Importantly, the polymer did not diminish enzymatic activity but only moderately delayed the reaction, extending the time required to reach maximal cleavage from ~60 to ~90 min. Molecular dynamics simulations combined with CD calculations demonstrated that DNA binding induces a stable left-handed helical organization of the polymer. In contrast, DNA shortening following cleavage promotes conformational reorganization responsible for the observed chiroptical response. 

## 5. Conclusions

Polythiophenes belong to a class of π-conjugated polymers that feature an extended system of delocalized π-electrons, which give rise to many useful electrical and optical properties. They can serve as powerful semiconducting and fluorescent materials in optoelectronic devices. The chiral π-conjugated systems effectively translate side-chain stereocenters or dynamic external templates (such as chiral liquid crystals) into higher-order helical aggregates that exhibit distinct circular dichroism (CD) and circularly polarized luminescence (CPL). The ability to tune their supramolecular organization and optical activity makes chiral polythiophenes versatile candidates for optoelectronics and asymmetric catalysis while due to their capacity for stereospecific binding, selective guest-molecule recognition, and conformational reorganization (including helical assembly), they are envisioned as chiral sensors for bioorganic and pharmaceutical applications. 

Recent developments in the preparation of chiral polythiophenes have established targeted synthetic methodologies such as Pd-catalyzed cross-coupling polymerizations including Stille and Suzuki polycondensations, the RuPhos protocol (a modified Negishi coupling reaction), direct C–H coupling polymerization, along with Ni-catalyzed cross-coupling polymerizations exemplified by Kumada catalyst-transfer polycondensation (KCTP), dehydrobrominative polycondensation using a Knochel–Hauser base, and then FeCl_3_-mediated oxidative coupling procedure, or asymmetric electropolymerization. While FeCl_3_ oxidative coupling is widely used for scalable synthesis, its step-growth/free-radical-like mechanism typically yields broader dispersity (PDI > 2) and lower regioregularity; controlled chain-growth methods like KCTP or Pd-RuPhos Negishi coupling, in turn, provide precise control of the backbone regioregularity and molecular weight of polymers. Nevertheless, industrially viable methods for large-scale production must feature high regioregularity, batch-to-batch reproducibility, and low cost. Two strategies meet most of these requirements: Grignard metathesis/Kumada catalyst-transfer polycondensation and electrochemical polymerization. KCTP polymerization delivers regioregular (head-to-tail coupling) polymers with narrow dispersity and precise molecular weights. It represents a robust, industrially viable platform for the large-scale production of regioregular poly(3-hexylthiophene) (rr-P3HT). Beyond its primary role as a widely studied p-type semiconductor in organic solar cells and transistors, poly(3-hexylthiophene) has rapidly become a key functional polymer for light-actuated bioelectronic interfaces. P3HT-based functional materials offer many advantages, including tunable mechanical (stretchable, thermally stable), optical (visible, fluorescent), and electrical (good conductivity) properties, as well as biocompatibility; for this reason, they are useful as well in bioelectronic devices. Therefore, material engineering has focused on screening different morphologies, such as nanoparticles and thin films, for better integration polymers with biological components [124]. KCTP allows scalable, reproducible production of the ether side-chained polythiophenes, predominantly used for glycolated polythiophenes (e.g., oligoethylene glycol-substituted P3HT analogues used in organic electrochemical transistors). In turn, standard PEDOT is primarily manufactured on an industrial scale via oxidative polymerization (e.g., Fe(III) tosylate or persulfate) to yield PEDOT:PSS (poly(styrenesulfonate)), direct arylation polymerizations or via electropolymerization [125,126]. On the other hand, electrochemical polymerization leads to a highly crystalline material formed directly on a conductive electrode. This strategy bypasses bulk polymer synthesis, isolation, and issues with insoluble materials. Direct deposition of in situ electrooxidized polymeric layers onto transparent conductive oxides (ITO) or microelectrodes as thin, uniform films makes this one-step methodology a cost- and time-saving protocol that is directly scalable for manufacturing integrated, enantioselective (bio)sensors. Regarding future perspectives in the commercialization of chiral polythiophenes, scalable synthetic platforms, such as controlled chain-growth KCTP for processable polymers or direct electropolymerization for thin films, are expected to combine tunable chiroptical features with cost-effective manufacturing.

## Figures and Tables

**Table 1:** Polymers composition and molar masses.

Product	Percentage of 63 Used	% HT Coupling	% HH Coupling	% TT Coupling	M*_n_* [kg/mol]	PDI
**60**	100	100	0	0	8.5	1.45
**61**	100	100	0	0	8.8	1.25
**62**	99	95	3	2	9.1	1.45
**63**	97	92	4	4	8.1	1.50
**64**	95	89	5	6	8.0	1.35
**65**	90	83	8	9	7.9	1.40
**66**	50	56	21	23	8.4	1.40

**Table 2:** Polymer Optical and Chiroptical Properties Summary.

Polymer Name	Absorption Max (λ_max_)	CD/Cotton Effect Profile (as Wavelength Decreases)	Final Conformation. Helical Assembly
**Poly(98)**	470 nm	Bisignate [1st (−), 2nd (+)]	Left-handed helical aggregation
**Poly(99)**	490 nm	Bisignate [1st (−), 2nd (+)]	Left-handed helical aggregation
**Poly(100)**	~550 nm	Bisignate [1st (+), 2nd (−)]	Right-handed helical aggregation
**Poly(101)**	~430 nm	Weak bisignate [1st (−), 2nd (+)]	One-handed twisted conformation with helical aggregates (high density of structural *syn* and α–β defects)
**Poly(102)**	~410 nm	Bisignate [1st (−), 2nd (+)] Non-zero-crossing	One-handed twisted conformation with helical aggregates (fewer defects than poly(**101**))
**Poly(103)**	346 nm	Bisignate [1st (−), 2nd (+)]	Highly twisted single chains (caused by steric hindrance of methyl groups) and helical aggregation
**Poly(105) and Poly(106)**	~450–490 nm	Bisignate [1st (−), 2nd (+)]	Helical aggregation
**Poly(EDOT)**	~550 nm	No detectable Cotton effect	Achiral/No helical aggregation (despite identical UV–vis/IR to poly(bis-EDOT))
**Poly(104)**	~550 nm	Distinct bisignate [1st (−), 2nd (+)]	Helical aggregation
**Poly(108)**	~430 nm	Intense bisignate [1st (+), 2nd (−)]	Right-handed helical aggregation
**Poly(109)**	Shorter wavelength ~350 nm	Bisignate [1st (−), 2nd (+)]	Helical aggregation (blue-shifted due to non-planar, unbridged biphenyl core)
**Poly(110)**	~430 nm	No clear Cotton effect	Achiral/No helical aggregation
**Poly(111)**	~560 nm (ICT) 1 band at 300–400 nm	2 or 3 bisignate peaks	Helical aggregation
**Poly(112)**	~580 nm (ICT)	2 or 3 bisignate peaks	Strong helical aggregation [bis(thienothiophene) acts as a stronger donor]

## Data Availability

No new data were created or analyzed in this study. Data sharing is not applicable to this article.
